# Stable Europium(III) Complexes with Short Linkers for Site‐Specific Labeling of Biomolecules

**DOI:** 10.1002/open.201700122

**Published:** 2017-09-04

**Authors:** Felix Faschinger, Martin Ertl, Mirjam Zimmermann, Andreas Horner, Markus Himmelsbach, Wolfgang Schöfberger, Günther Knör, Hermann J. Gruber

**Affiliations:** ^1^ Institute of Biophysics Johannes Kepler University Linz Gruber Straße 40 4040 Linz Austria; ^2^ Institute of Inorganic Chemistry Johannes Kepler University Linz Altenberger Straße 69 4040 Linz Austria; ^3^ Institute for Analytical Chemistry Johannes Kepler University Linz Altenberger Straße 69 4040 Linz Austria; ^4^ Institute of Organic Chemistry Johannes Kepler University Linz Altenberger Straße 69 4040 Linz Austria

**Keywords:** energy transfer, fluorescent probes, lanthanides, luminescence, macrocyclic ligands

## Abstract

In this study, two new terpyridine‐based Eu^III^ complexes were synthesized, the structures of which were optimized for luminescence resonance energy‐transfer (LRET) experiments. The complexes showed high quantum yields (32 %); a single long lifetime (1.25 ms), which was not influenced by coupling to protein; very high stability in the presence of chelators such as ethylenediamine‐*N*,*N*,*N*′,*N*′‐tetraacetate and ethylene glycol‐bis(2‐aminoethylether)‐*N*,*N*,*N*′,*N*′‐tetraacetic acid; and no interaction with cofactors such as adenosine triphosphate and guanosine triphosphate. A special feature is the short length of the linker between the Eu^III^ ion and the maleimide or hydrazide function, which allows for site‐specific coupling of cysteine mutants or unnatural keto amino acids. As a consequence, the new complexes appear particularly suited for accurate distance measurements in biomolecules by LRET.

## 1 Introduction

Lanthanide complexes have been successfully used in several fields of bioanalytics. Examples are homogeneous time‐resolved fluorescence (HTRF) assays;^1^ dissociation‐enhanced lanthanide fluorescent immunoassays (DELFIAs);^2^ cell imaging; and luminescence probes for oxygen, singlet oxygen, pH, or other analytes.^3^, ^4^, ^5^

A specialized biophysical application of lanthanide complexes is the measurement of intra‐ or intermolecular distances by luminescence resonance energy transfer (LRET),^6^, ^7^, ^8^ which may serve as an alternative to X‐ray and NMR spectroscopy. The latter methods can resolve very fine structural details, but their material and time demands are very high. Very little sample is required for fluorescence resonance energy transfer (FRET), but it gives only crude distance estimations, because the Förster distance can rarely be determined. For this reason, FRET is mainly used to screen the influence of mutations or buffer components upon conformational changes or interactions of biomolecules.^9^

For a number of reasons, LRET is better suited than FRET for explicit distance determination.^7^, ^8^ 1) The origin of error from the relative orientations of the donor and acceptor dyes is minimized because of the isotropic nature of the donor.^10^ 2) A wider range of distances can be analyzed if suitable donor–acceptor pairs are selected.^7^ 3) The narrow emission lines of the lanthanide donor allow selective measurement of the emission lifetime of the acceptor, as used in the “sensitized emission” method.^7^ 4) In LRET, the energy‐transfer efficiency (*E=*1−*τ*_DA_/*τ*_Donor_) can be measured more accurately by comparing the luminescence lifetime of the donor in the absence of the acceptor (*τ*_Donor_) versus in the presence of the acceptor (*τ*_DA_). Such a comparison of the lifetimes is much less susceptible to systematic errors than the comparison of emission intensities used in FRET. FRET relies on organic dyes or fluorescent proteins, which require setups resolving lifetimes in the nanosecond range, whereas Eu/Tb complexes used in LRET exhibit lifetimes in the range of 1 to 2.5 ms.

An essential requirement for energy‐transfer measurements by using lifetimes is the existence of a single lifetime of the lanthanide donor in the absence of an acceptor, both before and after coupling to the biomolecule of interest. The latter criterion is fulfilled by some but not all lanthanide complexes found in the literature.^5^, ^11^, ^12^ Another requirement is high thermodynamic and kinetic stability to prevent loss of the lanthanide ion in the presence of chelators such as ethylene glycol‐bis(2‐aminoethylether)‐*N*,*N*,*N*′,*N*′‐tetraacetic acid (EGTA).

**Figure 1:**
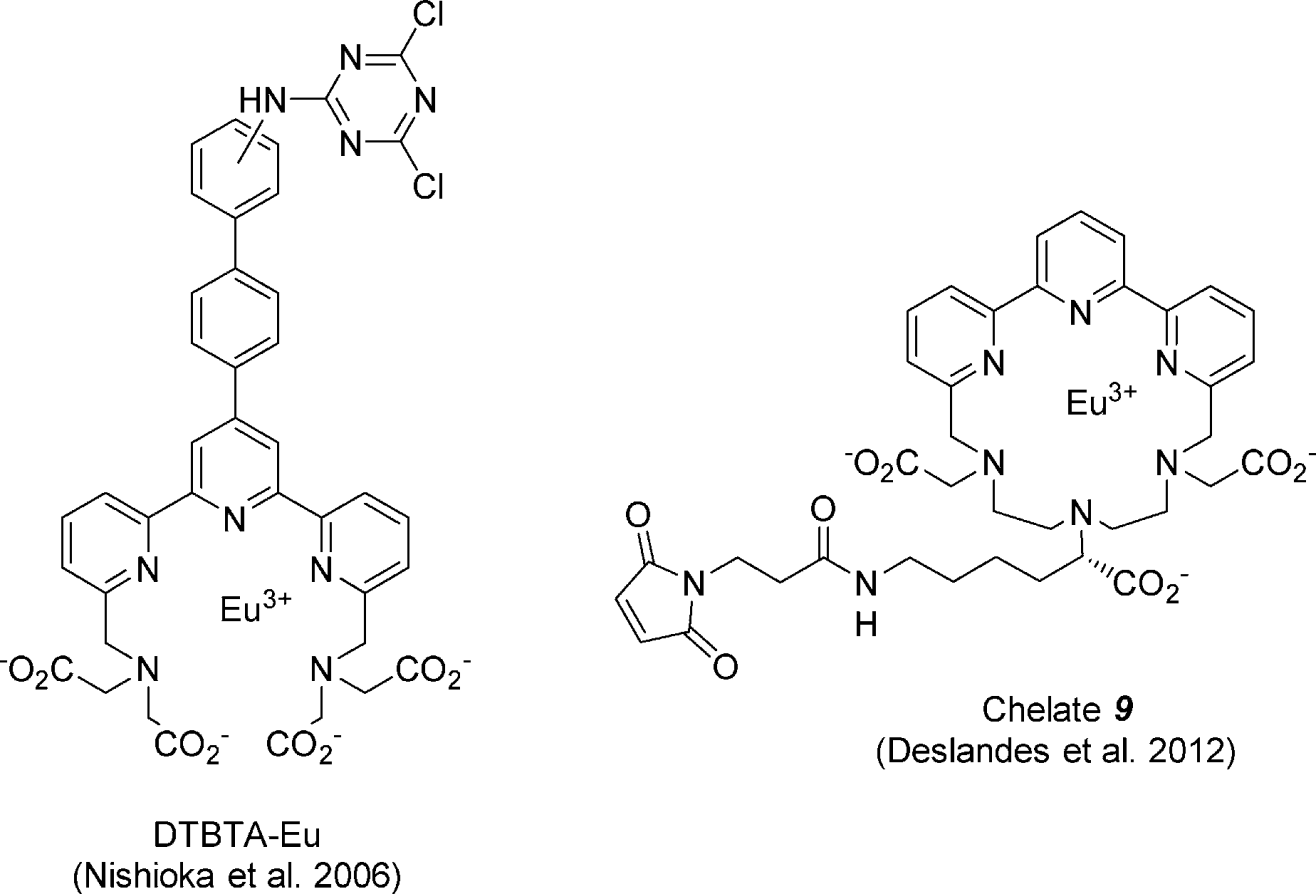
Example of known Eu complexes with linkers for protein coupling.

Several Eu/Tb complexes described in the literature fulfill these criteria but still cannot be used for accurate distance measurements, because they have unspecific coupling functions such as *N*‐hydroxysuccinimide esters or dichlorotriazinyl groups that lead to statistical labeling of lysine residues (exemplified by DTBTA–Eu in Figure 1).^5^, ^13^ Other complexes actually allow for site‐specific labeling of a cysteine mutant by their maleimide function; nevertheless, their use for distance measurements is compromised because they have a long, flexible linker between the lanthanide ion and the maleimide function (see chelate **9** in Figure 1).^12^

The goal of our study was to design a lanthanide complex that fulfills all requirements necessary for distance measurements by LRET, including site‐specific coupling and minimal linker length. For the parent structure, we chose terpyridine‐based chelators that show high thermodynamic and kinetic stability, due to the relatively rigid backbone and the high coordination numbers.^4^, ^5^, ^14^, ^15^, ^16^ In particular, we selected chelate **9** (Figure 1),^12^ in which the terpyridine is cyclized with a diethylene triamine skeleton. In this diethylene triamine unit, two nitrogen atoms are part of a glycine element and one nitrogen atom is part of a lysine residue. Herein, we introduce a much shorter linker between the complex and the maleimide function, and besides the maleimide moiety, we offer a hydrazide for site‐specific labeling of carbonyl functions.

## 2 Results and Discussion

**Scheme 1:**
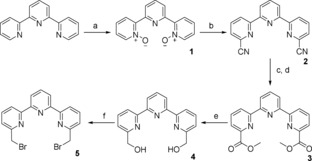
Synthesis of the dibromo terpyridine. Reagents and conditions: a) *m*CPBA (5.8 equiv), CH_2_Cl_2_, RT, 24 h, 47 %; b) (CH_3_)_3_SiCN (10 equiv), CH_2_Cl_2_, RT, 20 min, PhCOCl (4 equiv), RT, 12 h, 70 %; c) H_2_O/HOAc/concd H_2_SO_4_ (4:4:1), 90–100 °C, 24 h, 100 %; d) SOCl_2_ (10 equiv), MeOH, reflux, 18 h, 84 %; e) NaBH_4_ (5.7 equiv), EtOH, RT, 3 h, reflux, 1 h, 86 %; f) LiBr (1.1 equiv), PBr_3_ (4.9 equiv), DMF, RT, 4 h, 86 %.

**Scheme 2:**
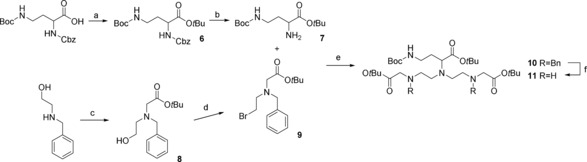
Synthesis of the 2,4‐diamonobutyric acid derivative of DTTA. Reagents and conditions: a) TBTA (2 equiv), BF_3_ **⋅**OEt_2_ (0.18 equiv), CH_2_Cl_2_, cyclohexane, RT, 12 h, 93 %; b) NH_4_HCO_2_ (10 equiv), Pd/C (0.08 equiv), 1‐propanol/MeOH (1:1), RT, 18 h, 96 %; c) *tert*‐butyl bromoacetate (1 equiv), DIPEA (1 equiv), DMF, 0–25 °C, 12 h, 100 %; d) PPh_3_ (1.18 equiv), NBS (1.17 equiv), CH_2_Cl_2_, 0–25 °C, 4 h, 45 %; e) **7** (1 equiv), **9** (2 equiv), K_2_CO_3_ (10 equiv), CH_3_CN, reflux, 18 h, 71 %; f) NH_4_HCO_2_ (42 equiv), Pd/C (0.25, equiv), 1‐propanol/MeOH (1:1), RT, 18 h, 84 %.

**Scheme 3:**
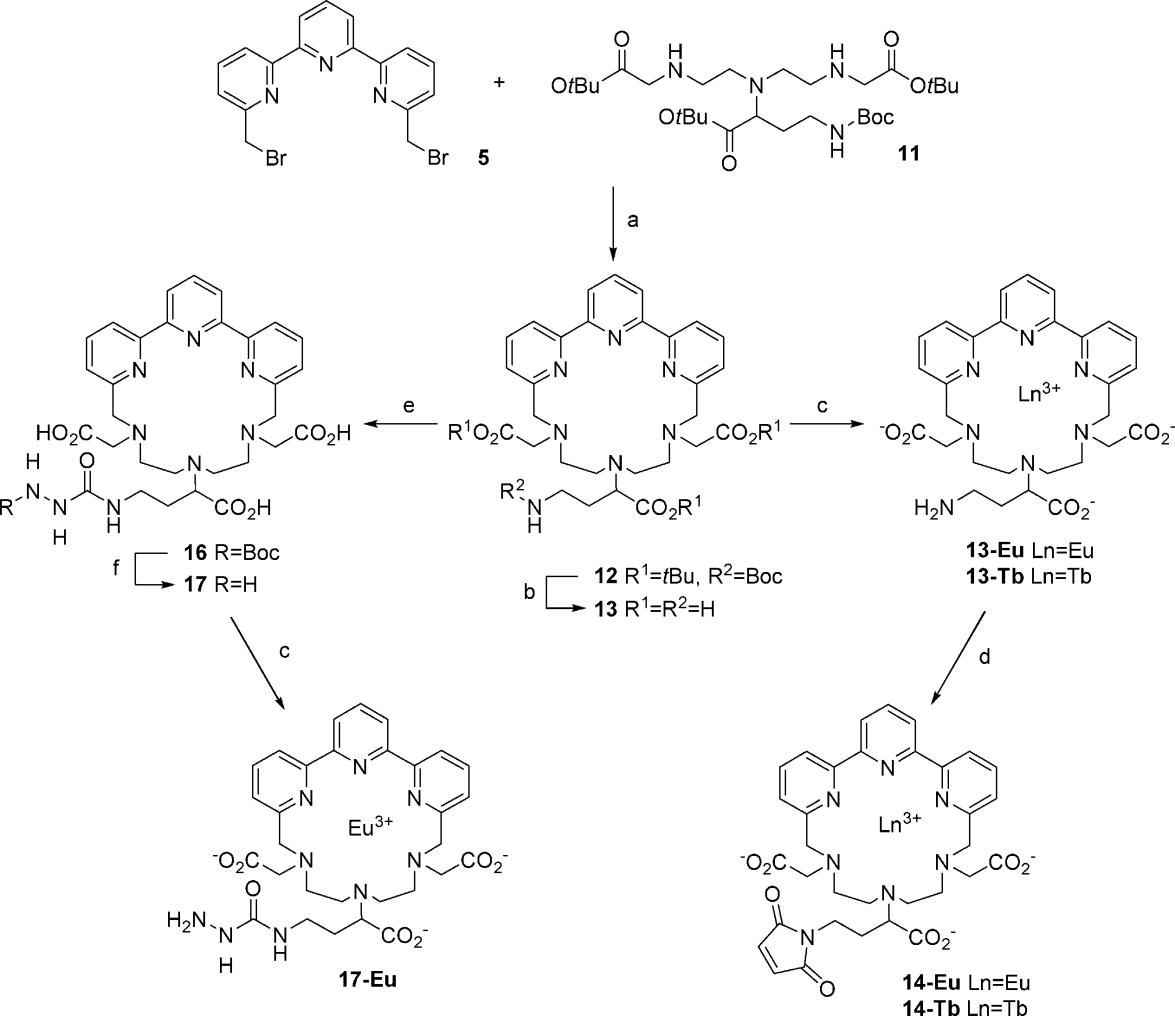
Synthesis of lanthanide complexes for site‐specific labeling. Reagents and conditions: a) **11** (1 equiv), **5** (1 equiv), Na_2_CO_3_ (10 equiv), CH_3_CN, 12 h, 50 %; b) TFA/CH_2_Cl_2_ (1:1), RT, 12 h, 96 %; c) 2 mm **13**/**17** in H_2_O, LnCl_3_ (1 equiv), pH 6.5, RT, 12 h, 100 %; d) *N*‐methoxycarbonylmaleimide (54 equiv), H_2_O/sat. NaHCO_3_ (1:11), 0 °C, 15 min, RT, 30 min, 60–89 %; e) **15** (2.0 equiv), DIPEA (16 equiv), DMF, RT, 12 h, 93 %; f) TFA/CH_2_Cl_2_ (1:1), RT, 12 h, 100 %.

The goal of this study was to synthesize analogues of chelate **9** (Figure 1) that carry either a maleimide or a hydrazide function on a much shorter linker than that found in chelate **9**. The general synthesis pathway was similar to that of chelate **9** (Figure 1):^12^ thus, compound **5** (Scheme 1), which is a bis‐bromomethyl derivative of terpyridine, and diethylenetriamine triacetate (DTTA) derivative **11** (Scheme 2) were assembled into cyclic chelate **12** (Scheme 3), and cyclic chelate **12** was further modified to yield the final lanthanide complexes.

One major difference from the published synthesis route of chelate **9** was a different method for the preparation of bis‐bromomethyl terpyridine **5**. Here, we used the methods reported for the preparation of DTBTA–Eu^III^ (Figure 1),^5^ depicted in Scheme 1.

The second deviation from the published synthesis of chelate **9** was the use of protected diaminobutyric acid rather than the longer lysine derivative as the starting material for DTTA derivative **11**. Except for this difference in the starting material, which implied several additional synthesis steps, the steps in Scheme 2 were analogous to those in the synthesis of chelate **9**.

The coupling of the bis‐bromomethyl terpyridine **5** with DTTA derivative **11** (Scheme 3, step a), removal of the protecting group (Scheme 3, step b), and lanthanide insertion (Scheme 3, step c) were performed as described for chelate **9**.^12^ However, the primary amino group was then directly converted into a maleimide group (Scheme 3, **14**‐Eu and **14**‐Tb),^17^, ^18^ which thereby avoided the additional increase in the length of the linker associated with attachment of the maleimidopropionyl group in chelate **9** (Figure 1).

The synthesis of carbazide derivatives **16**, **17**, and **17**‐Eu was essentially new. The hydrazide function in **17** is known for its chemoselective reaction with aldehydes and ketones in aqueous media, as used for site‐specific labeling of N‐terminal serine/threonine or oligosaccharide side chains after periodate treatment^19^, ^20^ or for coupling to site‐specifically incorporated unnatural amino acids containing a ketone function.^21^

### 2.1 Synthesis of 6,6′′‐Dibromomethyl‐2,2′:6′,2′′‐terpyridine 5

The synthesis of 6,6′′‐dibromomethyl‐2,2′:6′,2′′‐terpyridine **5** was performed in a manner similar to the strategy of Nishioka et al., starting with the terpyridine because the biphenyl side chain with the aromatic amine function was not required here.^5^, ^13^ In the first step, bis‐*N*‐oxide **1** was synthesized according to Nishioka et al.^5^ by using a 5.8‐fold excess amount of *meta*‐chloroperoxybenzoic acid (*m*CPBA). The yield of 47 % was moderate and can probably be improved by using a smaller amount of mCPBA and by washing during the workup with acetone rather than acetonitrile.^22^ The introduction of carbonitrile groups in the 6‐ and 6′′‐positions was conducted by using (CH_3_)_3_SiCN and benzoyl chloride in a modified Reissert–Henze reaction.^23^ The 70 % yield in the reaction for **2** is rather high for the regioselective reaction. The carbonitrile groups were hydrolyzed in acetic acid and concentrated sulfuric acid at 90–100 °C, and subsequently, the methyl ester was obtained by heating at reflux with thionyl chloride in methanol. After recrystallization from toluene, pure **3** was obtained in 84 % yield. The methyl ester was reduced by using sodium borohydride in ethanol, and important was the careful workup with saturated NaHCO_3_ to obtain pure **4** in 86 % yield. Bromide substitution of the hydroxy group was achieved with an approximately 4.9‐fold excess amount of PBr_3_ and LiBr in dry DMF. The dropwise addition of PBr_3_ at 0 °C was crucial, and the solution was red as long as active PBr_3_ was present in the solution. Due to the high excess amount of PBr_3_, the solution had to remain colored as long as unreacted alcohol **4** was present, for which reaction control was performed by TLC. After solvent removal and basic extraction, pure **5** was isolated in 86 % yield.

### 2.2 Synthesis of Diethylenetriamine Triacetate Derivative 11

The synthesis of DTTA derivative **11** was based on the strategies reported by Deslandes et al. and Bechara et al.^12^, ^14^, ^15^ The side‐chain amino group of 2,4‐diaminobutyric acid (Dab) was designed to carry the coupling function (e.g., maleimide) for site‐specific labeling. Commercially available Z‐Dab(Boc)‐OH**⋅**DCHA (Z=Cbz=benzyloxycarbonyl, Boc=tert‐butoxycarbonyl, DCHA=dicyclohexylammonium) was first transformed into the free acid by suspending it in ethyl acetate and adding 1 m phosphoric acid, whereupon everything dissolved in the two‐phase system. The *tert*‐butyl ester was introduced with *tert*‐butyl 2,2,2‐trichloroacetimidate (TBTA) by using BF_3_ as a catalyst in a mixture of cyclohexane and dichloromethane.^24^ Upon completion of the reaction and removal of the solvent, the residue was suspended in 10 % Na_2_CO_3_ for 2 h; otherwise, trichloroacetimidate derivative contaminants could not be removed (the contaminants were observed as two broad singlets at *δ*=5.8 and 6.6 ppm in the ^1^H NMR spectrum). Esterification attempts with di‐*tert*‐butyl dicarbonate and 4‐(*N*,*N*‐dimethylamino)pyridine (DMAP) as the catalyst were unsuccessful, whereas the method mentioned above gave **6** in 93 % yield. The benzyloxycarbonyl protecting group was removed by using ammonium formate as the hydrogen source with Pd/C as the catalyst.^25^, ^26^ Cleavage of the Cbz group of **6** was achieved in 96 % yield in a mixture of 1‐propanol and MeOH, which ensured complete solvation of **6** and ammonium formate. The primary amino group of *N*‐benzylethanolamine was alkylated in DMF with *tert*‐butyl bromoacetate to give **8** in quantitative yield. In the next step, alcohol **8** was converted into corresponding bromide **9** by an Appel reaction by using *N*‐bromosuccinimide (NBS) and triphenylphosphine. For this purpose, the reactants (except for NBS) were dried carefully by evaporation of dry toluene, and NBS was added in portions at 0 °C. The order of adding the reactants as well as the use of strictly dry CH_2_Cl_2_ were very important to obtain a product with proper quality. During purification by silica‐gel chromatography some product loss was unavoidable because the triphenylphosphine oxide fraction partially overlapped with the product fraction, and this is the reason behind the moderate yield of 45 %. The α‐amino group of **7** was alkylated with 2 equivalents of bromide **9** in dry refluxing acetonitrile with solid potassium carbonate as the base.^12^ Extraction and purification by column chromatography gave pure **10** in 71 % yield. The method for cleavage of the benzyl group was related to the method described above for cleavage of the Cbz group. Thus, diamine **11** was obtained in 84 % yield by using ammonium formate in MeOH/1‐propanol, yet by using a higher amount of the Pd catalyst than that used for cleavage of the Cbz group.^25^, ^26^

### 2.3 Synthesis of a Sulfhydryl‐Reactive Lanthanide Complex

Activated terpyridine **5** was treated with a stoichiometric amount of DTTA derivative **11** in refluxing acetonitrile with sodium carbonate as the base. The crude product was extracted with brine and not with ethylenediamine‐*N*,*N*,*N*′,*N*′‐tetraacetate (EDTA) solution, as described by Deslandes et al.^12^ Pure complex precursor **12** was isolated after column chromatography in a yield comparable (50 %) to that given in their report.^12^ Cleavage of the Boc and *tert*‐butyl ester groups was performed in trifluoroacetic acid (TFA)/CH_2_Cl_2_ (1:1) at a concentration of 12.05 mm **12** to obtain **13** in 96 % yield.

**Scheme 4:**
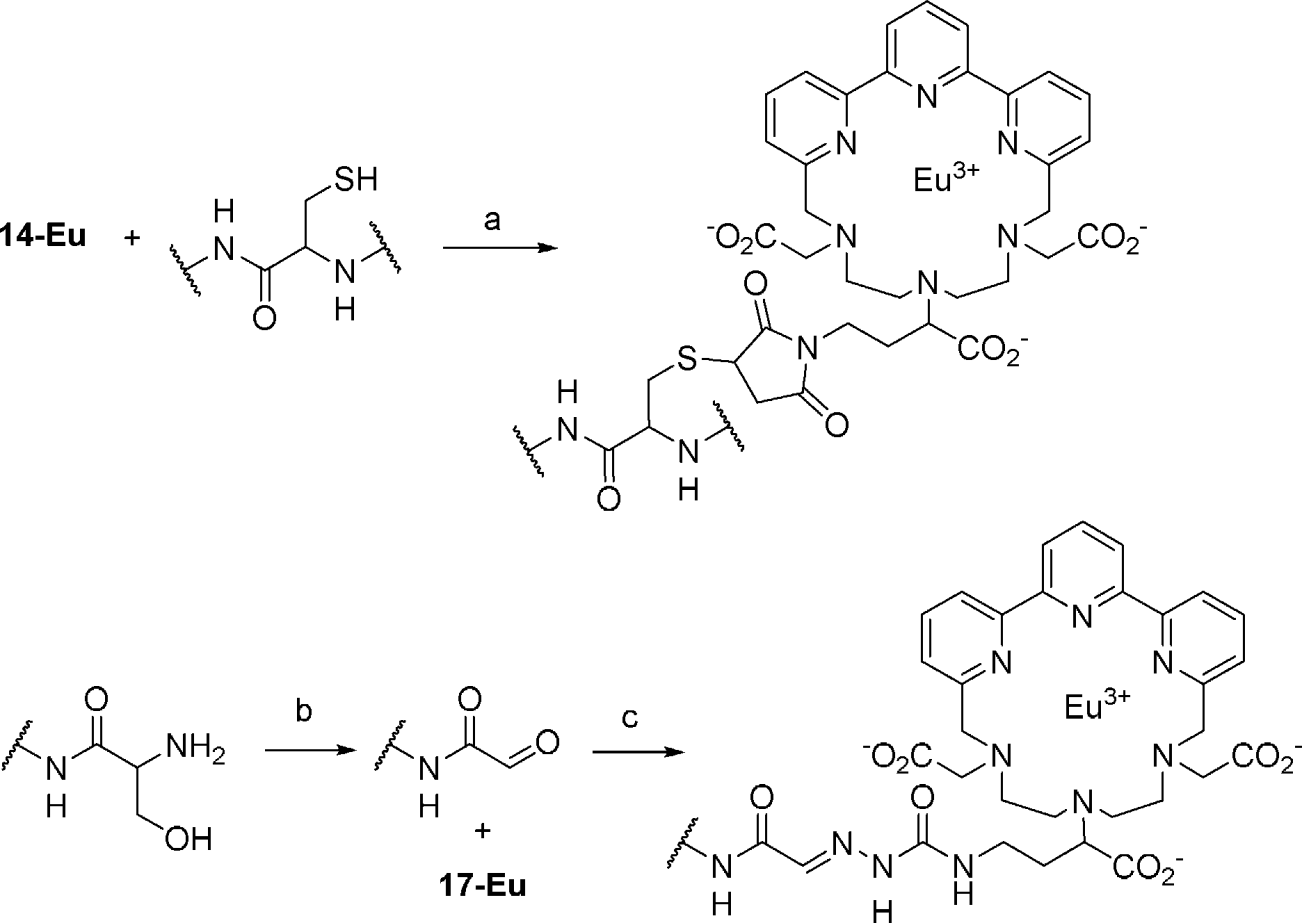
Site‐specific labeling reaction of a cysteine‐containing peptide or protein with **14**‐Eu (top) and of a periodate‐treated N‐terminal serine (or threonine) with **17**‐Eu (bottom). Reagents and conditions: a) Buffer pH 7.5, tris(2‐carboxyethyl)phosphine, 1 mm EDTA;^27^ b) sodium periodate, pH 6.5;^20^ c) acetate buffer pH 4.5.^20^

To synthesize a sulfhydryl‐reactive probe, the lanthanide ion was introduced into chelate **13**. Metalation was achieved by combining aqueous solutions of lanthanide(III) chloride and a stoichiometric amount of **13**, whereas the pH was repeatedly adjusted to 6.5 with dilute NaOH. Finally, the primary amino group of **13**‐Eu/Tb was directly converted into a maleimide group with *N*‐methoxycarbonylmaleimide in saturated NaHCO_3_ by using a method described originally by Keller and Rudinger.^17^ Management of the temperature and time was very important during the reaction, and to quickly stop the reaction, the pH was raised to 6. The appearing solid byproducts were filtered off, and the product‐containing solution was adjusted with water to an appropriate volume for purification of **14**‐Eu/Tb by HPLC. Reverse‐phase HPLC was performed by applying a gradient from water to acetonitrile by using 0.1 % acetic acid as an additive. Purification resulted in 60 % yield of **14**‐Eu and 89 % yield in the case of **14**‐Tb. The site‐specific labeling reaction of **14**‐Eu with a cysteine‐containing peptide or protein is illustrated in Scheme 4 at the top.

### 2.4 Synthesis of a Carbonyl‐Reactive Lanthanide Complex

**Scheme 5:**
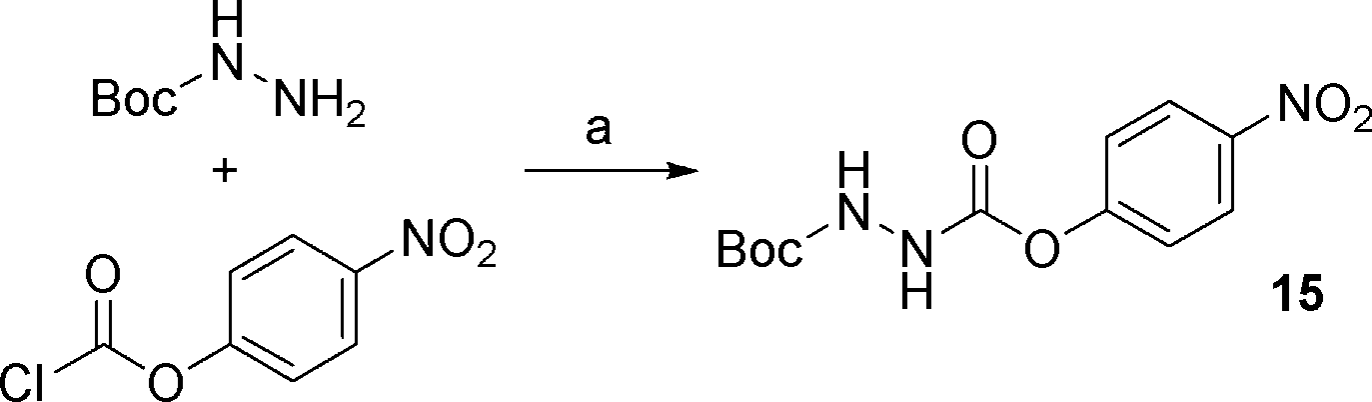
Synthesis of Boc‐aza‐Gly 4‐nitrophenyl ester **15**: Reagents and conditions: a) Boc‐hydrazine (2.0 equiv), 4‐nitrophenyl chloroformate (1.0 equiv), THF, RT, 12 h, 96 %.

The site‐specific labeling of glycoproteins or N‐terminal serines/threonines is achieved by periodate oxidation and subsequent reaction with a hydrazide linker (a model reaction of N‐terminal serine with **17**‐Eu is presented in Scheme 4 at the bottom).^19^, ^20^ Amine‐reactive nitrophenyl ester **15** was synthesized from Boc‐hydrazine and 4‐nitrophenyl chloroformate in dry THF according to the protocol of Gante and Weitzel in 96 % yield (Scheme 5).^28^ Coupling of **15** with **13** was achieved in dry DMF with *N*,*N*‐diisopropylethylamine (DIPEA) (Scheme 3, step e). Purification was performed by HPLC (method B) with an aqueous acetic acid system, and elution of the pure product (93 % yield) was achieved by applying a gradient of increasing acetonitrile content. The Boc group of **16** was quantitatively cleaved in TFA/CH_2_Cl_2_ (1:1), and metalation of **17** was performed by using the procedure described for **13**. Final product **17**‐Eu was isolated by evaporation of water. Thereby, some sodium chloride was formed as a byproduct, which originated from adjustment of the pH. It was not removed, because it did not influence further steps.

### 2.5 Luminescent BSA Conjugate

**Figure 2:**
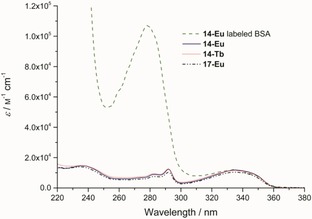
Absorption spectra of BSA labeled with **14**‐Eu, **14**‐Eu, **14**‐Tb, and **17**‐Eu in HBS 7.3. The values were renormalized to the appropriate extinction coefficients at *λ*=335 nm (11 900, 11 580, and 10 420 m ^−1^ cm^−1^ for **14**‐Eu, **14**‐Tb, and **17**‐Eu, respectively) to estimate the labeling efficiency of BSA with **14**‐Eu.

Bovine serum albumin (BSA) was chosen for labeling with sulfhydryl‐reactive complex **14**‐Eu, because on average every second BSA molecule possesses one accessible sulfhydryl group.^29^ The coupling was performed overnight in 2‐[4‐(2‐hydroxyethyl)piperazin‐1‐yl]ethanesulfonic acid (HEPES)‐buffered saline (HBS) 7.3 containing 1 mm EDTA under an argon atmosphere. In contrast to the general procedure shown in Scheme 4, the reducing agent, tris(2‐carboxyethyl)phosphine (TCEP), was omitted for two reasons: 1) TCEP cleaves important disulfides in BSA, which causes destabilization and the appearance of additional thiol groups. 2) The endogenous free cysteine of BSA is known to resist oxidation for a very long time.^29^ The labeled BSA was isolated by size‐exclusion chromatography, and a labeling efficiency of 91 % was estimated by comparing the extinction coefficients of BSA^30^ and **14**‐Eu (for the spectra, see Figure 2; also see calculations in the Supporting Information).

### 2.6 Photophysical Properties

**Figure 3:**
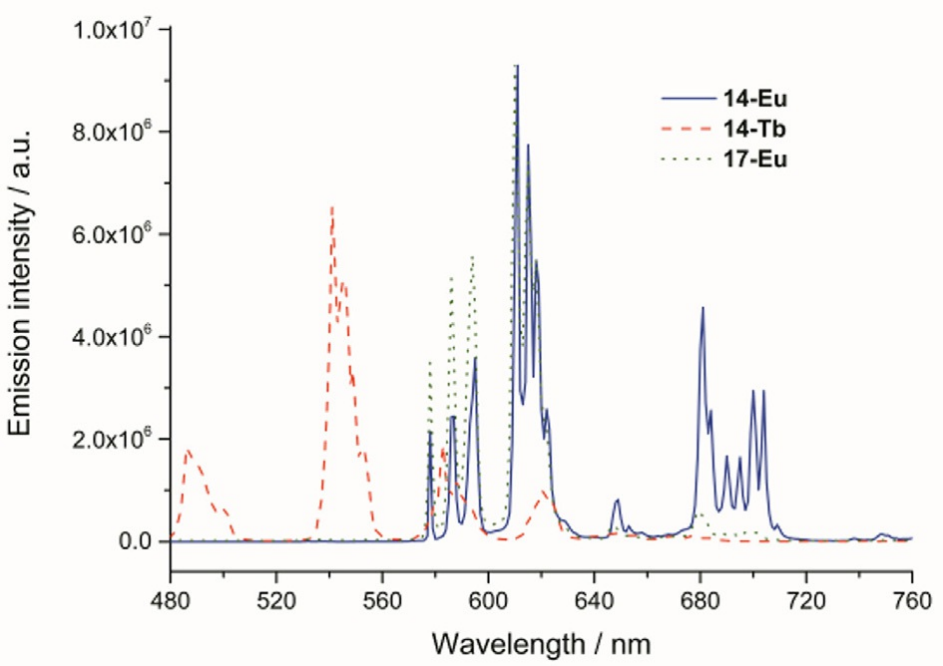
Emission spectra of **14**‐Eu, **14**‐Tb, and **17**‐Eu in HBS 7.3. The excitation was performed at *λ*=335 nm with excitation and emission slits of 1 nm. The spectrum of **17**‐Eu was measured with a spectrometer with poorer sensitivity in the long‐wavelength region.

The most important photophysical data are summarized in Table 1. The absorption spectra of chelates **14**‐Eu, **14**‐Tb, and **17**‐Eu and that of **14**‐Eu‐labeled BSA are shown in Figure 2. The characteristic luminescence spectra of **14**‐Eu, **17**‐Eu, and **14**‐Tb in HBS 7.3 are presented in Figure 3. The europium emission bands arise from ^5^D_0_→^7^F_*J*_ (*J=*0–4) transitions, and the strongest emission is at *λ*=611 nm (*J=*2). The terbium bands result from ^5^D_4_→^7^F_*J*_ (*J=*6–3) transitions, and the strongest emission is at *λ*=541 nm (*J=*5).

**Table 1:** Spectroscopic properties of **14**‐Eu, **14**‐Tb, **17**‐Eu, and **14**‐Eu‐labeled BSA.

Compound	*λ*_max_ [nm]	*ϵ* [m ^−1^ cm^−1^]	*Φ* ^[a]^ [%]	*τ*_Donor_ [μs]	*τ* ${}_{D{}_{2}O}$ ^[b]^ [μs]	*q* ^[c]^
**14**‐Eu	236	14 700	32.3±2.0	1197±5	1767±8	0.02±0.01
**14**‐Eu on BSA	227	107 100 (278 nm)	28.8±2.3	1254±7	1765±13	−0.02±0.01
**14**‐Tb	236	14 300	18.4±2.4	961±8 205±19	–	–
**17**‐Eu	235	13 900	–	1199±6	–	–

[a] Quantum yield was determined by the relative method by using rhodamine 6G as a standard.^31^, ^32^ The measurement was performed in aerated HBS 7.3. Excitation was performed at *λ*=335 nm by using a 1 nm slit for emission and excitation. The data were corrected for changes in the refractive indices. [b] Measured in deuterated water for **14**‐Eu and deuterated HBS 7.3 for **14**‐Eu‐labeled BSA. [c] The hydration state *q* was calculated by using the equation *q*=1.2(1000/*τ*_Donor_−1000/*τ* ${}_{D{}_{2}O}$ −0.25), in which *τ*_Donor_ is the lifetime in nondeuterated buffer and *τ* ${}_{D{}_{2}O}$ is the lifetime in deuterated buffer.^33^

**Figure 4:**
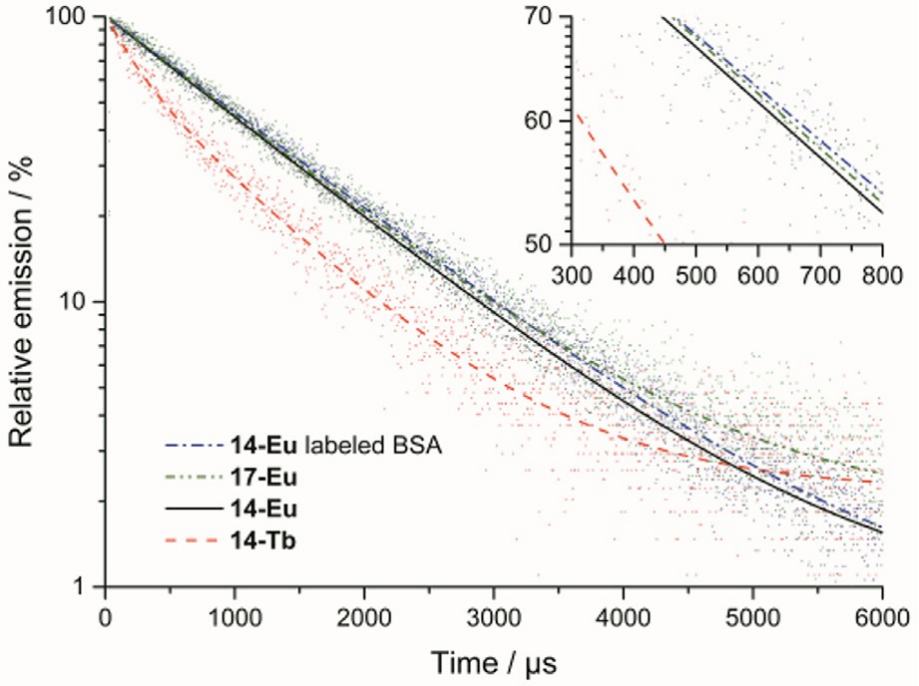
Lifetimes of **14**‐Eu‐labeled BSA, **17**‐Eu, **14**‐Eu, and **14**‐Tb in HBS 7.3 at a concentration of 3 μm. Chelates **14**‐Eu and **17**‐Eu show single exponential decays, which were undisturbed by protein coupling, whereas **14**‐Tb exhibits biexponential behavior with two shorter lifetimes. The inset illustrates the parallel time courses for all Eu complexes, which reflects equal lifetimes.

The luminescent quantum yield was determined by a relative method by using rhodamine 6G as a standard.^31^, ^32^ The data were collected in aerated HBS 7.3 and were corrected for changes in refractive indices (see Figure S1 in the Supporting Information). The quantum yield determined for **14**‐Eu [*Φ*=(32.2±2.0) %] is comparable to the highest values measured for Eu complexes.^34^ Coupling **14**‐Eu to BSA affected the quantum yield only marginally [*Φ*=(28.8±2.3) %], which led to the conclusion that conjugation to proteins has no adverse effect. Tb‐complex **14**‐Tb had a rather poor quantum yield [*Φ*=(18.4±2.4) %] relative to those of other known Tb complexes. Analogous observations were also reported by Bechara et al.^14^, ^15^ A similar distinction between Eu and Tb was seen upon comparing the lifetimes: **14**‐Eu and **17**‐Eu showed slow single‐exponential decays with *τ*=(1197±5) and (1199±6) μs, respectively. These long lifetimes are comparable to those of other Eu complexes with good luminescence properties. In contrast to the Eu complexes, **14**‐Tb showed two lifetimes, one with *τ*=(961±8) μs and the other one with *τ*=(205±19) μs. The banana‐shaped curve presented in Figure 4 (red dashed line) is a clear indicator of this multiexponential decay. The fact that **14**‐Tb possesses a low quantum yield and two lifetimes, both of which are shorter than expected for a good Tb complex, demonstrates that **14**‐Tb is not suited for LRET experiments.

The lifetimes of free **14**‐Eu and BSA‐bound **14**‐Eu were measured in nondeuterated and deuterated buffer (Figure S2 and Table 1). A general reason for the shorter lifetimes in nondeuterated buffer is the presence of water in the outer hydration shell of the lanthanide complexes.^33^ In deuterated buffer, free **14**‐Eu and BSA‐bound **14**‐Eu exhibited the same lifetimes (Table 1). In nondeuterated buffer, however, BSA‐bound **14**‐Eu showed a small but significant increase in the lifetime from *τ*=(1197±5) to (1254±7) μs. Using the method of Beeby et al.,^33^ the *q* values for free **14**‐Eu and BSA‐bound **14**‐Eu were calculated as 0.02±0.01 and −0.02±0.01, respectively. This *q* value is interpreted as the number of water molecules in the inner coordination sphere.^33^ We cannot be fully sure whether the measured effect is truly significant, but if so, then the data suggest reduced access of water to Eu^III^ in the BSA‐bound state.

Chelates **14**‐Eu and **17**‐Eu were intended for labeling of proteins and for measuring distances within proteins or between interacting proteins. Many proteins are Ca^II^ dependent and require EGTA for adjustment of defined Ca^II^ concentrations. In such cases, it is important that Eu^III^ cannot be extracted by EGTA but that it remains bound. The luminescence intensity of **17**‐Eu was monitored over a period of at least 24 h with 2 mm EDTA, EGTA, or Ca‐EGTA (2 mm EGTA and 2.1 mm CaCl_2_ resulting in 100 μm free Ca^II^ ion concentration)^35^ in the buffer. No decrease in signal intensity was observed over the whole 24 h time period (see Figure S3), and the characteristic emission spectrum of **17**‐Eu was fully conserved (data not shown), which implies that Eu^III^ stays tightly bound to the chelate. Apart from signal intensity, the lifetime of **14**‐Eu‐labeled BSA listed in Table 2 also remained unchanged in the presence of 2 mm EDTA or EGTA. Numerous proteins require metal ions such as zinc(II) or copper(II) for their proper function. It is known that some lanthanide complexes possess low stability against zinc(II) and copper(II), which cause transmetalation with the lanthanide.^36^ For this reason, the stability of **17**‐Eu (3 μm) in the presence of 10 μm Zn^II^ (physiological concentration^37^) and 200 μm Zn^II^ was examined by monitoring the luminescence intensity for more than 24 h (Figure S3). No change in luminescence intensity was observed, which indicates that the Eu complex could be used in the presence of physiological zinc(II) concentrations without any concerns. Analogous measurements were also performed with 20 μm (physiological concentration^38^) and 200 μm copper(II) sulfate. In both cases, the luminescence was also constant for 24 h, but the total luminescence in 200 μm copper(II) sulfate was already at the beginning of the experiment 1.5 times lower than that in absence of Cu^II^ (data not shown). This was attributed to the colloidal nature of the 200 μm copper(II) sulfate solution at pH 7.3.

**Table 2:** Lifetimes of **14**‐Eu‐labeled BSA in HBS 7.3 (3 μm) under different conditions (see curves in Figure S5).

Additive^[a]^	Buffer	*τ* ^[b]^ [μs]
–	HBS 7.3	1254±7
–	50 mm phosphate	1227±11
2 mm ATP	HBS 7.3	1250±5
2 mm AMP	HBS 7.3	1248±13
2 mm adenosine	HBS 7.3	1232±8
2 mm GTP	HBS 7.3	1244±6
2 mm EDTA	HBS 7.3	1256±5
2 mm EGTA	HBS 7.3	1255±3

[a] The pH of the nucleotide, adenosine, EGTA, or EDTA stock solution was adjusted to pH 7.3 before it was used as an additive in the experiments. [b] All lifetimes exhibited a monoexponential decay and were fitted to the equation *I*=*I*_0_+*I*_1_⋅*e* ^−(*t*/*τ*)^, in which *I* is the total luminescence intensity, *I*_0_ is time‐independent background light, *I*_1_ is the luminescence intensity from the Eu emission, *t* is the time and *τ* is the luminescence lifetime of the Eu emission.

Lanthanide ions bound to lanthanide‐binding tags and some lanthanide complexes were reported to be sensitive to inorganic phosphate, so that physiological phosphate buffers could not be used.^6^, ^39^ No such sensitivity to phosphate was seen in BSA‐bound **14**‐Eu during 15 min exposure to 50 mm phosphate buffer (Table 2).

**Figure 5:**
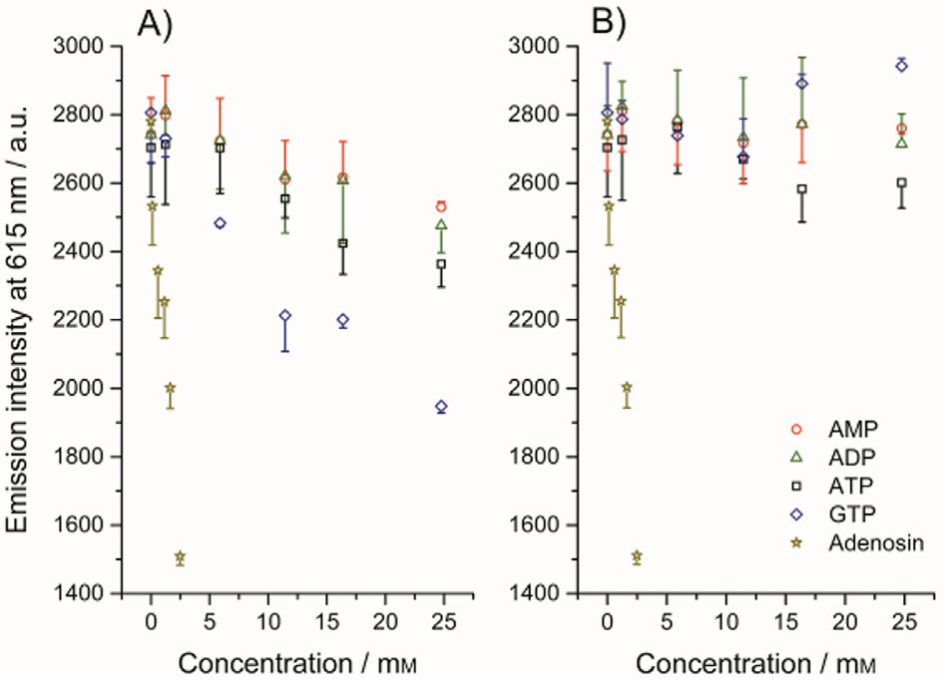
The emission of BSA‐linked **14**‐Eu in HBS 7.3 (3 μm) was monitored at *λ*=615 nm (2.5 nm slit) with excitation at *λ*=335 nm (10 nm slit), while titrating with different nucleotides or adenosine. a) Emission intensities were corrected for the dilution caused by the addition of the 100 mm nucleotide stock solution (the stock solution of adenosine was 10 mm). b) Data were in addition to panel A corrected for the filter effect caused by the nucleotide solutions. The error bars are shown to one side only to minimize overlapping of the data.

Weitz et al. reported quenching of a luminescent Tb complex by millimolar concentrations of adenosine 5′‐monophosphate (AMP), adenosine 5′‐diphosphate (ADP), and especially adenosine 5′‐triphosphate (ATP), which was explained by photoelectron transfer caused by stacking of the nucleobases with the antenna molecule.^40^ Fortunately, no indication of interactions with nucleotides was found with BSA‐bound **14**‐Eu. Slow gradual titration with up to 25 mm ATP, ADP, and AMP caused minor changes in the emission intensity of BSA‐bound **14**‐Eu (Figure 5), and prolonged incubation at the highest concentration showed completely stable emission (Figure S4). The luminescence lifetime of **14**‐Eu‐labeled BSA remained constant after the addition of 2 mm AMP or ATP (see Table 2 and Figure S5). Guanosine 5′‐triphosphate (GTP) caused a stronger concentration dependence of the emission intensity of **14**‐Eu‐bound BSA (see Figure 5 a), but this was not due to quenching, as explained in the following. The reason for the concentration dependence of the emission intensity is the redshifted absorption spectrum of GTP (or of a contamination commonly present in commercial GTP), which caused absorption of the excitation beam used to excite **14**‐Eu (for the absorption spectra of the nucleotide stock‐solutions, see Figure S6) in the fluorescence cuvette. This “filter effect” caused a GTP‐concentration‐dependent decrease in the emission intensity of the Eu complex (Figure 5 a), which disappeared once we corrected the data for the absorption caused by the GTP component (Figure 5 b). Moreover, the emission intensity was not dependent on the time of GTP incubation (Figure S4), and the lifetime of **14‐**Eu‐labeled BSA was also unaffected by GTP (Table 2, Figure S5). Only adenosine caused a strong concentration‐dependent reduction in **14**‐Eu emission, which was clearly not just due to a filter effect, because the absorption of adenosine at *λ*=335 nm was too small for this purpose. By analogy to the findings of Weitz et al., stacking of adenosine with the antenna of the chelate could be responsible for the intensity loss.^40^ The higher tendency of adenosine (relative to ATP, ADP, and AMP) to form stacks with **14**‐Eu seems plausible in light of its much lower solubility, due to the lack of a phosphate group. Interestingly, adenosine influenced only the luminescence intensity of **14**‐Eu‐labeled BSA in a concentration‐dependent manner (Figure 5) but not the luminescence lifetime (Table 2 and Figure S5). Fortunately, only nucleotides (e.g., ATP, ADP, AMP, and GTP) but not nucleosides (e.g., adenosine) are typically required as important cofactors for in vitro studies, for which distances are measured by LRET. In conclusion, new complex **14**‐Eu appears to be unperturbed by most buffer components that are to be expected.

At a given concentration of GTP, the long light path inside a fluorimeter cuvette causes a much larger reduction in the excitation intensity than the short light path of an inverted microscope, for which the imaged volume is located immediately above the surface of the glass slide. Unfortunately, the Nd:YAG laser used in our inverted microscope had an excitation wavelength of only 266 nm, which is very close to the absorption maximum of the nucleotides. To compensate for signal loss due to the filter effect caused by the nucleotides, the laser power was increased, and this resulted in decay curves with slightly increased background signals. This becomes clear upon comparing the curves with and without nucleotides in Figure S5. In spite of this increased background, the fitted lifetimes summarized in Table 2 were not noticeably influenced by the higher background and gave the same results within experimental uncertainty.

## 3 Conclusions

New terpyridine‐based Eu complexes were designed and synthesized for site‐specific labeling of thiols and carbonyls in proteins, peptides, and nucleic acids. The Eu complexes were found to possess high thermodynamic and kinetical stability, as previously observed for similar complexes. The high quantum yield and the long lifetimes were not affected by coupling to proteins or by typical buffer components such as ethylenediamine‐*N*,*N*,*N*′,*N*′‐tetraacetate, ethylene glycol‐bis(2‐aminoethylether)‐*N*,*N*,*N*′,*N*′‐tetraacetic acid, phosphate, calcium(II), zinc(II), copper(II), and nucleotides. These findings show that the new Eu complexes fulfill the crucial requirements for distance measurements by luminescence resonance energy transfer (LRET). Unlike the Eu complex, the Tb complex of the same chelator was not suitable for LRET, as it exhibited two lifetimes, both of which were shorter than that of the Eu complex.

The most significant new feature of the new Eu complexes was the fact that the linkers were much shorter than those in comparable published lanthanide chelates. The 2,4‐diaminobutyric acid linker module is shorter than the lysine linker in a comparable complex, and the terminal amine of this linker was not extended with an additional heterobifunctional linker; instead, it was directly converted into a maleimide or a hydrazide function, with minimal length extension. The maleimide and hydrazide derivatives could be used for site‐specific labeling of biomolecules by relying on simple well‐established coupling protocols. The short length of the linker is expected to minimize systematic errors upon measuring intra‐ and intermolecular distances by LRET. Presently, we are examining this crucial aspect in appropriate test systems.

## Experimental Section

### General Methods

Analytical‐grade solvents and reagents were used as long as they were commercially available. 2,2′:6′,2“‐Terpyridine, *N*‐bromosuccinimide (NBS), benzoyl chloride (PhCOCl), lithium bromide (ultradry, 99.998 %), phosphorus tribromide, *N* ^α^‐benzyloxycarbonyl‐*N* ^γ^‐*tert*‐butyloxycarbonyl‐l‐2,4‐diaminobutyric acid dicyclohexylammonium salt [Z‐Dab(Boc)‐OH**⋅**DCHA], europium(III) chloride hexahydrate (99.99 %), terbium(III) chloride hexahydrate (99.99 %), trimethylsilyl cyanide [(CH_3_)_3_SiCN], trifluoroacetic acid, and adenosine were purchased from Alfa Aesar. *tert*‐Butyl trichloracetimidate (TBTA), *N*‐benzylethanolamine, boron trifluoride diethyl ether (BF_3_ **⋅**OEt_2_), *tert*‐butyl carbazate, *meta*‐chloroperoxybenzoic acid (*m*CPBA), adenosine 5′‐triphosphate (ATP) disodium salt hydrate, guanosine 5′‐triphosphate (GTP) sodium salt hydrate, adenosine 5′‐monophosphate (AMP) sodium salt hydrate, ethylene glycol‐bis(2‐aminoethylether)‐*N*,*N*,*N*′,*N*′‐tetraacetic acid (EGTA), and disodium dihydrogen ethylenediamine‐*N*,*N*,*N*′,*N*′‐tetraacetate (EDTA) were ordered from Sigma–Aldrich. Sodium borohydride and *N*‐methoxycarbonylmaleimide were purchased from Fluka. Dry acetonitrile, dry *N*,*N*‐dimethylformamide (DMF), and 4‐nitrophenyl chloroformate were obtained from Acros Organics, whereas dry ethanol, triphenylphosphine (PPh_3_), and dry *N*,*N*‐diisopropylethylamine (DIPEA) were obtained from Merck Millipore. HBS 7.3 [150 mm NaCl, 5 mm 2‐[4‐(2‐hydroxyethyl)piperazin‐1‐yl]ethanesulfonic acid (HEPES) adjusted with NaOH to pH 7.3] and 50 mm phosphate buffer (sodium dihydrogen phosphate monohydrate was adjusted with NaOH to pH 7.5) were used in the experiments. Toluene and dichloromethane were dried and stored over 4 Å molecular sieves (Merck) under an argon atmosphere. Dry peroxide‐free tetrahydrofuran (THF) was prepared by passing twice through a bed of basic alumina (60, Merck) of approximately the same volume as the THF and was used immediately. Reactions requiring an inert atmosphere were performed under argon 5.0. Thin‐layer chromatography (TLC) was performed on Merck Millipore 60 Å silica sheets without a fluorescence indicator. TLC staining was performed in a chamber with sublimating iodine. For selective staining of primary or Boc‐protected amines, a solution of 0.1 % ninhydrin in acetic acid/1‐butanol mixture (1:50, *v*/*v*) was sprayed onto the TLC plates, and subsequently, the plates were heated to 120–150 °C until violet spots became visible. Column chromatography was performed on silica gel (Davisil, 70–200 μm, porosity 60 Å). Reverse‐phase (RP) HPLC was performed by using a Dionex P680 HPLC pump with a Dionex PDA 100 detector and a polymer based reverse phase column (Hamilton PRP‐3, 10 μm, 300 Å, 305×7.0 mm). The flow rate was 7 mL min^−1^, and the absorbance was monitored at *λ*=210, 230, 280, 335, and 359 nm. Method A: The solvents were 3 % acetonitrile in water (solvent A), acetonitrile (solvent B), and 1 % acetic acid in water (solvent C). The compounds were separated by applying a gradient from solvent A to solvent B, while keeping the fraction of solvent C constant at 10 %. The solvent gradient started with 0 % solvent B for 10 min, and solvent B was then linearly increased to 22 % from 10 to 45 min and then kept at 22 % for 10 min. Finally, the column was regenerated with 90 % solvent B for 15 min. Method B: Solvents A, B, and C were the same as in method A, and the fraction of solvent C was also kept constant at 10 %. The fraction of solvent B was kept constant during the initial 10 min and was then raised from 0 to 90 % within 60 min, and finally kept constant at 90 % for another 15 min. Size‐exclusion chromatography was performed in HBS 7.3 at 4 °C with a GE Healthcare (Little Chalfont, UK) ÄKTA pure system equipped with a Superdex 200 increase column (10 mm×300 mm, GL). The flow rate was 0.5 mL min^−1^, and the absorbance was monitored at *λ*=280 and 335 nm.

### Physical Measurements

^1^H NMR and ^13^C NMR spectra were recorded with a Bruker Avance 300 MHz spectrometer, but in some cases, a Bruker Avance‐III 700 MHz spectrometer was used. Chemical shifts are given in parts per million according to the solvent signal. Electrospray (ES) mass spectra were obtained with an Agilent MSD SL ion‐trap mass spectrometer (Agilent, Waldbronn, Germany), and high‐resolution mass spectrometry (HRMS) was performed with a LTQ Orbitrap XL from Thermo Fisher Scientific (Waltham, MA, USA), operating in positive‐ion mode by using electrospray ionization. Absorption measurements were performed with a Hitachi U‐3010 or an Agilent Cary 300. Emission spectra were measured with a Hitachi F‐4500 or a Horiba Fluorolog‐3–22. The quantum yields were determined in aerated HBS 7.3 by the relative method^41^ by using rhodamine 6G (*Φ*=94 %)^31^, ^32^ as a standard. The quantum yield determinations were performed with a Cary 300 UV/Vis spectrophotometer and a Fluorolog‐3–22 fluorimeter, whereby the data were corrected for changes in refractive indices. Excitation of reference standard and samples was performed at *λ*=335 nm, and the concentration was held below an absorbance value of 0.1. During all the measurements, the same quartz glass cuvette was used, and the orientation of the cuvette in the spectrometers was always identical.

### Stability of the Eu Complexes

The stability was assessed with a Hitachi F‐4500 by using a quartz glass cuvette with dimensions of 8.5 mm×5 mm×2 mm (w×h×d; the small window 5 mm×2 mm was used for excitation, and the large window 8.5 mm×5 mm was used for emission detection). For the measurements, excitation was at *λ*=335 nm (10 nm slit), emission was observed at *λ*=615 nm (2.5 nm slit), and the photomultiplier voltage was set to 700 V. To a solution of **14**‐Eu‐labeled BSA in HBS 7.3 (3 μm, 120 μL) increasing amounts of a 100 mm stock solution of nucleotides (adjusted to pH 7.3) were added at 3 min intervals, until a concentration of approximately 25 mm was reached (the measurements were performed in triplicate). In the case of adenosine, a 10 mm stock solution (adjusted to pH 7.3) was used, and it was added up to a final concentration of about 2.5 mm. Solutions with the highest nucleotide concentrations were monitored at 1 min intervals for at least 10 min to examine changes due to prolonged incubation. The luminescence intensity of **17**‐Eu in HBS 7.3 (3 μm) in the presence and absence of 2 mm EDTA, 2 mm EGTA, 2 mm Ca‐EGTA buffer, 10 μm zinc(II) chloride, and 200 μm zinc(II) chloride buffer was monitored over more than 24 h to assess the stability of the complex against competitors. The EDTA (350 mm) and the EGTA (100 mm) stock solutions were adjusted to pH 7.3 prior to the preparation of the final dilutions. For the Ca‐EGTA buffer, the EGTA concentration was 2 mm and the CaCl_2_ concentration was 2.1 mm, which resulted in a free Ca^II^ ion concentration of 100 μm according to Schoenmakers et al.^35^ Control experiments were performed by measuring solutions containing EuCl_3_ (3 μm) and EDTA (2 mm) or EGTA (2 mm) in HBS 7.3.

### Lifetime Measurements

The time‐resolved measurements were performed with a home‐built setup, and the exact measurement conditions are described in the Supporting Information.

### Syntheses

The syntheses of compounds **1**–**5**,^5^ **8**,^14^, ^15^ **9**,^14^, ^15^ and Z‐Dab(Boc)‐OH (according to the manufacturer's procedure) were performed in analogy to published procedures and are described in the Supporting Information.

*tert*‐Butyl *N* ^α^‐Benzyloxycarbonyl‐*N* ^γ^‐*tert*‐butyloxycarbonyl‐l‐2,4‐diaminobutyrate (**6**): Z‐Dab(Boc)‐OH (805 mg, 2.29 μmol) was dissolved in dry CH_2_Cl_2_ (5 mL), and a solution of TBTA (818 μL, 4.6 mmol, 2.0 equiv) in dry cyclohexane (10 mL) was added under an argon atmosphere. BF_3_ **⋅**OEt_2_ (50.8 μL, 411 μmol, 0.18 equiv) was added as a catalyst, and the mixture was stirred overnight. The precipitate was filtered off and washed with CH_2_Cl_2_. The combined CH_2_Cl_2_ layer was taken to dryness under reduced pressure. Saturated Na_2_CO_3_ was added to the oily residue, and the mixture was stirred for 2 h. CHCl_3_ was added to the suspension, the phases were separated, and the organic solution was washed with 10 % Na_2_CO_3_ (2×) and 0.1 m NaH_2_PO_4_ (2×). The organic solution was dried with Na_2_SO_4_, and the solvent was removed under reduced pressure to give **6** (870.3 mg, 2.13 mmol, 93 %). ^1^H NMR (300 MHz, CDCl_3_, 25 °C, Me_4_Si): *δ*=1.44 (s, 9 H, C(C*H*_3_)_3_), 1.45 (s, 9 H, C(C*H*_3_)_3_), 1.60–1.72 (m, 1 H, β‐CH^I^_2_), 1.99–2.10 (m, 1 H, β‐CH^II^_2_), 2.94–3.05 (m, 1 H, γ‐CH^I^_2_), 3.37–3.45 (m, 1 H, γ‐CH^II^_2_), 4.30 (dt, ^3^ *J*_H,H_=8.7 Hz, ^3^ *J*_H,H_=4.2 Hz, 1 H, α‐CH), 5.11 (s, 2 H, C*H*_2_‐C_6_H_5_), 5.14 (br s, 1 H, N*H*), 5.53 (d, ^3^ *J*_H,H_=7.6 Hz, 1 H, N*H*), 7.32–7.37 ppm (m, 5 H, aromatic CH of C_6_H_5_); ^13^C NMR (75 MHz, CDCl_3_, 25 °C, Me_4_Si): *δ*=28.1 (3 C, C(*C*H_3_)_3_), 28.6 (3 C, C(*C*H_3_)_3_), 33.8 (1 C, β‐CH_2_), 36.8 (1 C, γ‐CH_2_), 52.3 (1 C, α‐CH), 67.2 (1 C, *C*H_2_‐C_6_H_5_), 79.4 (1 C, *C*(CH_3_)_3_), 82.6 (1 C, *C*(CH_3_)_3_), 128.2 (2 C, *ortho*‐CH of C_6_H_5_), 128.3 (1 CH, *para*‐CH of C_6_H_5_), 128.7 (2 C, *meta*‐CH of C_6_H_5_), 136.4 (1 C, *ipso*‐C of C_6_H_5_), 156.1 (1 C, C carbonyl), 156.5 (1 C, C carbonyl), 171.5 ppm (1 C, C carbonyl); HRMS (ESI+): *m*/*z* (%): 409.2334 [*M*+H]^+^ (100), 426.2598 [*M*+NH_4_]^+^ (40), 431.2151 [*M*+Na]^+^ (10), 817.4587 [2 *M*+H]^+^ (15), 839.4406 [2 *M*+Na]^+^ (30) (C_21_H_32_N_2_O_6_ requires 409.2333 [*M*+H]^+^, 426.2599 [*M*+NH_4_]^+^, 431.2153 [*M*+Na]^+^, 817.4594 [2 *M*+H]^+^, 839.4413 [2 *M*+Na]^+^).

*tert*‐Butyl *N* ^γ^‐*tert*‐Butyloxycarbonyl‐l‐2,4‐diaminobutyrate (**7**): Compound **6** (165 mg, 404 μmol) was dissolved in 1‐propanol (1.7 mL), and 10 % Pd/C (33 mg, 31 μmol, 0.08 equiv) was added. Ammonium formate (264 mg, 4.2 mmol, 10 equiv) was separately dissolved in MeOH (1.7 mL) and added under an argon atmosphere. The suspension was stirred at RT overnight. The suspension was filtered through Celite and washed with 1‐propanol, and the solvent was evaporated under vacuum. The crude product was dissolved in CHCl_3_ and washed with saturated NaHCO_3_ (2×). The organic solution was dried with Na_2_SO_4_, and the solvent was removed under reduced pressure to give **7** (106.8 mg, 389 μmol, 96 %). ^1^H NMR (300 MHz, CDCl_3_, 25 °C, Me_4_Si): *δ*=1.44 (s, 9 H, C(C*H*_3_)_3_), 1.47 (s, 9 H, C(C*H*_3_)_3_), 1.59–1.70 (m, 1 H, β‐CH^I^_2_), 1.89–1.99 (m, 1 H, β‐CH^II^_2_), 3.19–3.29 (m, 1 H, γ‐CH^I^_2_), 3.31–3.37 (m, 1 H, γ‐CH^II^_2_), 3.39 (dd, ^3^ *J*_H,H_=8.7 Hz, ^3^ *J*_H,H_=4.5 Hz, 1 H, α‐CH), 5.23 ppm (br s, 1 H, NH); ^13^C NMR (75 MHz, CDCl_3_, 25 °C, Me_4_Si): *δ*=28.2 (3 C, C(*C*H_3_)_3_), 28.6 (3 C, C(*C*H_3_)_3_), 34.6 (1 C, β‐CH_2_), 38.2 (1 C, γ‐CH_2_), 53.9 (1 C, α‐CH), 79,2 (1 C, *C*(CH_3_)_3_), 81.4 (1 C, *C*(CH_3_)_3_), 156.1 (1 C, C carbonyl of Boc), 175.2 ppm (1 C, *C*OOC(CH_3_)_3_); HRMS (ESI+): *m*/*z* (%): 275.1963 [*M*+H]^+^ (100), 297.1780 [*M*+Na]^+^ (10), 549.3850 [2 *M*+H]^+^ (35), 571.3667 [2 *M*+Na]^+^ (10) (C_13_H_26_N_2_O_4_ requires 275.1965 [*M*+H]^+^, 297.1785 [*M*+Na]^+^, 549.3858 [2 *M*+H]^+^). 571.3677 [2 *M*+Na]^+^).

*tert*‐Butyl *N* ^*α*^,*N* ^α^‐Bis({*N*‐benzyl‐*N*‐[(2‐*tert*‐butoxy)‐2‐oxoethyl]}‐2‐aminoethyl) *N* ^γ^‐*tert*‐Butyloxycarbonyl‐2,4‐diaminobutyrate (**10**): Compound **7** (219 mg, 798 μmol, dried by evaporating three times with dry toluene) and K_2_CO_3_ (1.1 g, 8.0 mmol, 10 equiv) were dissolved/suspended in dry acetonitrile (27 mL) and heated at reflux for 1 h. In the meantime, **9** (524 mg, 1.60 mmol, 2 equiv) was dissolved in dry acetonitrile (5.4 mL) and added to the stirred suspension. The mixture was stirred overnight at reflux temperature, and the turnover was checked by TLC (silica gel, CHCl_3_/MeOH/acetic acid=9:1:0.1, *R*_f_=0.83). After complete conversion, the mixture was filtered, and the solid was washed with acetonitrile. The combined acetonitrile layer was taken to dryness under reduced pressure, and the obtained residue was dissolved in CHCl_3_ and washed with 10 % Na_2_CO_3_ (2×) and brine (1×). The organic layer was dried with Na_2_SO_4_, and the solvent was removed under vacuum to obtain a crude product (507 mg). Purification was done by chromatography (silica gel, gradient elution starting with CHCl_3_/*n*‐heptane=3:2, and *n*‐heptane was stepwise decreased, followed by pure chloroform and finally increasing the MeOH content stepwise to CHCl_3_/MeOH=95:5) to give **10** (433 mg, 563 μmol, 71 %). ^1^H NMR (300 MHz, CDCl_3_, 25 °C, Me_4_Si): *δ*=1.38–1.46 (m, 36 H, C(C*H*_3_)_3_), 1.60–1.83 (m, 2 H, β‐CH_2_), 2.57–2.86 (m, 8 H, N‐C*H*_2_‐C*H*_2_‐N), 3.01–3.15 (m, 1 H, γ‐CH^I^_2_), 3.22 (s, 4 H, C*H*_2_‐COOC_4_H_9_), 3.26–3.41 (m, 2 H, γ‐CH^II^_2_ and α‐CH), 3.70–3.83 (m, 4 H, C*H*_2_‐C_6_H_5_), 6.02 (t, ^3^ *J*_H,H_=5.4 Hz, 1 H, NH), 7.21–7.32 ppm (m, 10 H, C_6_ *H*_5_); ^13^C NMR (75 MHz, CDCl_3_, 25 °C, Me_4_Si): *δ*=28.35 (3 C, C(*C*H_3_)_3_), 28.39 (3 C, C(*C*H_3_)_3_), 28.62 (3 C, C(*C*H_3_)_3_), 29.2 (1 C, β‐CH_2_), 37.4 (CH_2_, γ‐CH_2_), 49.3 (2 C, N‐*C*H_2_‐CH_2_‐N‐CH_2_‐*C*H_2_‐N), 52.4 (2 C, N‐CH_2_‐*C*H_2_‐N‐*C*H_2_‐CH_2_‐N), 55.2 (2 C, *C*H_2_‐C_6_H_5_ or *C*H_2_‐COOC_4_H_9_), 58.2 (2 C, *C*H_2_‐C_6_H_5_ or *C*H_2_‐COOC_4_H_9_), 60.4 (1 C, α‐CH), 78.7 (1 C, *C*(CH_3_)_3_), 80.9 (1 C, *C*(CH_3_)_3_), 81.0 (1 C, *C*(CH_3_)_3_), 127.3 (2 C, *para*‐CH of C_6_H_5_), 128.4 (4 C, *meta*‐CH of C_6_H_5_), 129.4 (4 C, *ortho*‐CH of C_6_H_5_), 138.7 (2 C, *ipso*‐C of C_6_H_5_), 156.2 (1 C, C carbonyl of Boc), 170,7 (2 C, C carbonyl of *t*‐butyl ester), 172,3 ppm (1 C, C carbonyl of *t*‐butyl ester); HRMS (ESI+): *m*/*z* (%): 769.5098 [*M*+H]^+^ (100), 791.4913 [*M*+Na]^+^ (10) (C_43_H_68_N_4_O_8_ requires 769.5110 [*M*+H]^+^, 791.4929 [*M*+Na]^+^).

*tert*‐Butyl *N* ^*α*^,*N* ^α^‐Bis({*N*‐[(2‐*tert*‐butoxy)‐2‐oxoethyl]}‐2‐aminoethyl) *N* ^γ^‐*tert*‐Butyloxycarbonyl‐2,4‐diaminobutyrate (**11**): Compound **10** (420 mg, 546 μmol) was dissolved in 1‐propanol (4.5 mL), and 10 % Pd/C (143 mg, 134 μmol, 0.25 equiv) was added. Ammonium formate (1.43 g, 22.6 mmol, 42 equiv) was separately dissolved in MeOH (4.5 mL) and was added under an argon atmosphere. The suspension was stirred at RT overnight. The suspension was filtered through Celite and washed with 1‐propanol, and the solvent was evaporated under vacuum. The crude product was dissolved in CHCl_3_ and washed with 10 % Na_2_CO_3_ (2×). The organic solution was dried with Na_2_SO_4_, and the solvent was removed under reduced pressure to give **11** (270.1 mg, 459 μmol, 84 %). ^1^H NMR (300 MHz, CDCl_3_, 25 °C, Me_4_Si): *δ*=1.437 (s, 9 H, C(C*H*_3_)_3_), 1.444 (s, 9 H, C(C*H*_3_)_3_), 1.465 (s, 18 H, C(C*H*_3_)_3_), 1.65–1.80 (m, 1 H, β‐CH^I^_2_), 1.86–1.93 (m, 1 H, β‐CH^II^_2_), 2.55–2.90 (m, 8 H, N‐C*H*_2_‐C*H*_2_‐N), 3.09–3.25 (m, 1 H, γ‐CH^I^_2_), 3.32 (d, ^3^ *J*_H,H_=2.5 Hz, 4 H, C*H*_2_‐COOC_4_H_9_), 3.26–3.45 (m, 2 H, γ‐CH^II^_2_ and α‐CH), 6.72 ppm (t, ^3^ *J*_H,H_=4.9 Hz, 1 H, NH); ^13^C NMR (75 MHz, CDCl_3_, 25 °C, Me_4_Si): *δ*=28.3 (6 C, C(*C*H_3_)_3_), 28.4 (3 C, C(*C*H_3_)_3_), 28.7 (3 C, C(*C*H_3_)_3_), 29.2 (1 C, β‐CH_2_), 37.2 (1 C, γ‐CH_2_), 47.6 (2 C, NH‐*C*H_2_‐CH_2_‐N‐CH_2_‐*C*H_2_‐NH), 50.6 (2 C, *C*H_2_‐COOC_4_H_9_), 51.5 (2 C, NH‐CH_2_‐*C*H_2_‐N‐*C*H_2_‐CH_2_‐NH), 60.4 (1 C, α‐CH), 78.5 (1 C, *C*(CH_3_)_3_), 81.1 (1 C, *C*(CH_3_)_3_), 81.2 (2 C, *C*(CH_3_)_3_), 156.5 (1 C, *C*OOC_4_H_9_), 172.0 (2 C, *C*OOC_4_H_9_), 172.2 ppm (1 C, *C*OOC_4_H_9_); HRMS (ESI+): *m*/*z* (%): 589.4164 [*M*+H]^+^ (100), 611.3974 [*M*+Na]^+^ (15) (C_29_H_56_N_4_O_8_ requires 589.4171 [*M*+H]^+^, 611.3990 [*M*+Na]^+^).

Di‐*tert*‐butyl 2,2′‐(8‐{1‐(*tert*‐Butoxy)‐4‐[(*tert*‐butoxycarbonyl)amino]‐1‐oxobutan‐2‐yl}‐5,8,11‐triaza‐1,2,3(2,6)‐tripyridinacyclododecaphane‐5,11‐diyl)diacetate (**12**): Compound **11** (175 mg, 297 μmol) and **5** (125 mg, 297 μmol, 1 equiv) were dried by azeotropic distillation (3×) with dry toluene under reduced pressure and dissolved in dry acetonitrile (110 mL). Na_2_CO_3_ (315 mg, 2.97 mmol, 10 equiv) was added, and the suspension was heated at reflux overnight under an argon atmosphere (with an argon bubbler connected to the outlet of the reflux condenser). The suspension was filtered and washed with acetonitrile and CHCl_3_, and the solvent was evaporated under vacuum. The crude product was dissolved in CHCl_3_ and washed with brine (2×). The organic solution was dried with Na_2_SO_4_, and the solvent was removed under reduced pressure. The crude mixture was purified by chromatography (silica gel, gradient elution starting with CHCl_3_/*n*‐heptane=98:2, followed by CHCl_3_, increasing the MeOH content stepwise and ending with CHCl_3_/MeOH=9:1) to give **12** (126 mg, 149 μmol, 50 %). ^1^H NMR (300 MHz, CDCl_3_, 25 °C, Me_4_Si): *δ*=1.17 (s, 27 H, C(C*H*_3_)_3_), 1.36 (s, 9 H, C(C*H*_3_)_3_), 1.75–1.89 (m, 2 H, β‐CH_2_), 2.63–3.00 (m, 8 H, N‐C*H*_2_‐C*H*_2_‐N), 3.06–3.19 (m, 6 H, C*H*_2_‐COOC_4_H_9_ and γ‐CH_2_), 3.65 (d, ^3^ *J*_H,H_=6.7 Hz, 1 H, α‐CH), 3.96 (d, ^2^ *J*_H,H_=12.9 Hz, 2 H, N‐C*H* ^I^_2_(*meso*)‐C‐N), 4.19 (d, ^2^ *J*_H,H_=12.8 Hz, 2 H, N‐C*H* ^II^_2_(*meso*)‐C‐N), 5.00 (br s, 1 H, NH), 7.35 (d, ^3^ *J*_H,H_=7.5 Hz, 2 H, CH of 5 and 5′′ in terpyridine), 7.92 (t, ^3^ *J*_H,H_=7.7 Hz, 2 H, CH of 4 and 4′′ in terpyridine), 8.01 (d, ^3^ *J*_H,H_=7.6 Hz, 2 H, CH of 3 and 3′′ in terpyridine), 8.11 ppm (s, 3 H, CH of 3′, 4′ and 5′ in terpyridine); ^13^C NMR (75 MHz, CDCl_3_, 25 °C, Me_4_Si): *δ*=26.4 (1 C, β‐CH_2_), 27.9 (9 C, C(*C*H_3_)_3_), 28.5 (3 C, C(*C*H_3_)_3_), 38.7 (1 C, γ‐CH_2_), 49.5 (2 C, N‐CH_2_‐*C*H_2_‐N‐*C*H_2_‐CH_2_‐N), 53.2 (2 C, N‐*C*H_2_‐CH_2_‐N‐CH_2_‐*C*H_2_‐N), 57.1 (2 C, *C*H_2_‐COOC_4_H_9_), 57.8 (2 C, N‐*C*H_2_(*meso*)‐C‐N), 60.9 (1 C, α‐CH), 79.2 (1 C, *C*(CH_3_)_3_), 82.0 (2 C, *C*(CH_3_)_3_), 82.6 (1 C, *C*(CH_3_)_3_), 120.5 (2 C, CH of 3 and 3′′ in terpyridine), 121.7 (2 C, CH of 3′ and 5′ in terpyridine), 124.0 (2 C, CH of 5 and 5′′ in terpyridine), 138.5 (2 C, CH of 4 and 4′′ in terpyridine), 139.2 (1 C, CH of 4′ in terpyridine), 155.0 (2 C, C of 2, 2′′ or 2′, 6′ in terpyridine), 155.3 (2 C, C of 2, 2′′ or 2′, 6′ in terpyridine), 156.1 (1 C, C of *C*OOC_4_H_9_), 158.1 (2 C, C of 6 and 6′′ in terpyridine), 171.8 (2 C, C of CH_2_‐*C*OOC_4_H_9_), 173.2 ppm (1 C, C of *C*OOC_4_H_9_); HRMS (ESI+): *m*/*z* (%): 846.5128 [*M*+H]^+^ (90), 868.4941 [*M*+Na]^+^ (100) (C_46_H_67_N_7_O_8_ requires 846.5124 [*M*+H]^+^, 868.4943 [*M*+Na]^+^).

2,2′‐[8‐(3‐Amino‐1‐carboxypropyl)‐5,8,11‐triaza‐1,2,3(2,6)‐tripyridinacyclododecaphane‐5,11‐diyl]diacetic acid (**13**): Compound **12** (20.4 mg, 24.1 μmol) was dissolved in dry CH_2_Cl_2_ (1 mL) and TFA (1 mL) to give a concentration of 12.05 mm for **12**. The mixture was stirred overnight at RT. Toluene was added to the solution, and the solvent was removed under reduced pressure. The resulting product was evaporated from toluene (1×), MeOH (3×), and water (1×) to give **13** (24 mg, 23.3 mol, 96 %). ^1^H NMR (300 MHz, CDCl_3_, 25 °C, Me_4_Si): *δ*=1.97–2.18 (m, 2 H, β‐CH_2_), 3.00 (t, ^3^ *J*_H,H_=6.3 Hz, 2 H, γ‐CH_2_), 3.13–3.21 (m, 2 H, N‐CH_2_‐C*H*_2_‐N‐C*H*_2_‐CH_2_‐N), 3.35–3.42 (m, 2 H, N‐CH_2_‐C*H*_2_‐N‐C*H*_2_‐CH_2_‐N), 3.48–3.51 (m, 1 H, α‐CH), 3.62–3.70 (m, 4 H, N‐C*H*_2_‐CH_2_‐N‐CH_2_‐C*H*_2_‐N), 3.74 (s, 4 H, C*H*_2_‐COOH), 4.72 (m, 4 H, N‐C*H*_2_(*meso*)‐C‐N shown in the HSQC), 7.80 (d, ^3^ *J*_H,H_=7.7 Hz, 2 H, CH of 5 and 5′′ in terpyridine), 8.27 (t, ^3^ *J*_H,H_=7.8 Hz, 2 H, CH of 4 and 4′′ in terpyridine), 8.43 (d, ^3^ *J*_H,H_=8.0 Hz, 2 H, CH of 3 and 3′′ in terpyridine), 8.61–8.72 ppm (m, 3 H, CH of 3′, 4′ and 5′ in terpyridine); ^13^C NMR (75 MHz, CDCl_3_, 25 °C, Me_4_Si): *δ*=23.3 (1 C, β‐CH_2_), 37.3 (1 C, γ‐CH_2_), 47.0 (2 C, N‐CH_2_‐*C*H_2_‐N‐*C*H_2_‐CH_2_‐N), 53.1 (2 C, N‐*C*H_2_‐CH_2_‐N‐CH_2_‐*C*H_2_‐N), 55.4 (2 C, *C*H_2_‐COOH), 57.3 (2 C, N‐*C*H_2_(*meso*)‐C‐N), 61.2 (1 C, α‐CH), 116.3 (1 C, ^1^ *J*_C,F_=291.7 Hz, *C*F_3_‐COOH), 124.3 (2 C, CH of 3 and 3′′ in terpyridine), 125.6 (2 C, CH of 3′ and 5′ in terpyridine), 128.4 (2 C, CH of 5 and 5′′ in terpyridine), 141.9 (2 C, CH of 4 and 4′′ in terpyridine), 147.16 (1 or 2 C, C of 2, 2′′ or CH of 4′), 147.19 (1 or 2 C, C of 2, 2′′ or CH of 4′), 148.1 (2 C, CH of 2′ and 6′ in terpyridine), 150.7 (2 C, CH of 6 and 6′′ in terpyridine), 162.9 (1 C, ^2^ *J*_C,F_=35.5 Hz, CF_3_‐*C*OOH), 170.4 (2 C, CH_2_‐*C*OOH), 173.6 ppm (1 C, CH‐*C*OOH); HRMS (ESI+): *m*/*z* (%): 578.2721 [*M*+H]^+^ (100), 600.2536 [*M*+Na]^+^ (15) (C_29_H_35_N_7_O_6_ requires 578.2722 [*M*+H]^+^, 600.2541 [*M*+Na]^+^).

General Procedure for Lanthanide Complex Formation: 2 mm Europium(III) chloride hexahydrate [or terbium(III) chloride hexahydrate] in water was added in portions to a 2 mm solution of the ligand in water. During the addition, the pH was repeatedly adjusted to 6.5 with dilute sodium hydroxide (150 mm). The solution was stirred overnight under an argon atmosphere at RT. Water was removed under reduced pressure to give the lanthanide complex in quantitative yield together with some sodium chloride.

Europium(III) 2,2′‐[8‐(3‐Amino‐1‐carboxylatopropyl)‐5,8,11‐triaza‐1,2,3(2,6)‐tripyridinacyclododecaphane‐5,11‐diyl]diacetate (**13**‐Eu): See the general procedure for preparation. ^1^H NMR (700 MHz, [D_4_]methanol, 25 °C): *δ*=49.20, 35.45, 29.15, 22.85, 20.08 18.23, 16.89, 14.93, 14.37, 11.18, 10.54, 7.27, 6.99, 5.60, 3.75, 3.42, 3.30, 2.66, 0.93, 0.74, 0.08, −0.90, −7.81, −14.01, −15.07, −17.86, −18.68, −21.20 ppm; ^13^C NMR (176 MHz, [D_4_]methanol, 25 °C): *δ*=125.4, 164.9, 119.1, 107.3, 111.8, 7.1, 93.1, 147.7, 111.8, 7.1, 141.4, 48.5, 35.4, 35.4, 62.3, 98.2, 71.6, 90.7, 71.6, 62.3, 42.6, 42.6, 29.5, 67.8, 132.7, 136.8, 142.1, 161.7, 165.0, 167.5, 174.2, 177.5, 189.9, 213.7 ppm; UV/Vis (H_2_O): *λ*_em_ (*ϵ*)=235 (14400), 283 (8300), 292 (11500), 335 nm (10900 mol^−1^ dm^3^ cm^−1^); HRMS (ESI+): *m*/*z* (%): 726.1681 [*M*+H]^+^ (85), 728.1695 [*M*+H]^+^ (100), 748.1498 [*M*+Na]^+^ (10), 750.1512 [*M*+Na]^+^ (12) (C_29_H_32_N_7_O_6_ requires 726.1685 [*M*+H]^+^, 728.1699 [*M*+H]^+^, 748.1505 [*M*+Na]^+^, 750.1519 [*M*+Na]^+^).

Europium(III) 2,2′‐{8‐[3‐(*N*‐maleimido)‐1‐carboxylatopropyl]‐5,8,11‐triaza‐1,2,3(2,6)‐tripyridinacyclododecaphane‐5,11‐diyl}diacetate (**14**‐Eu): Compound **13**‐Eu (8.8 mg, 12.1 μmol) was dissolved in water (280 μL), and saturated NaHCO_3_ (3.1 mL) was added under an argon atmosphere. The solution was stirred at 0 °C, and *N*‐methoxycarbonylmaleimide (101 mg, 654 μmol, 54 equiv) was added in one portion. After 15 min, the solution was warmed to RT and stirred for another 30 min. Subsequently, the pH of the solution was adjusted to pH 6 with 6 m HCl. The solution was concentrated under vacuum, and the solid was dissolved in a mixture of water (4 mL) and MeOH (0.6 mL). The solution was filter through cotton and was concentrated under vacuum to a volume of about 200 μL. The product was diluted with water to a total volume of 900 μL and was loaded on an HPLC column. The collected fractions were evaporated under vacuum to dryness. HPLC (method A): *t*_R_=19.3 min. Pure product **14**‐Eu (7.9 mg, 9.8 μmol, 60 %) was isolated. UV/Vis (HBS 7.3): *λ*_max_ (*ϵ*)=236 (14 700), 283 (8900), 292 (12 500), 335 nm (11 900 mol^−1^ dm^3^ cm^−1^); luminescence (HBS 7.3, *λ*_exc_=335 nm): *λ*_em_ (%)=578 (22), 586 (26), 595 (38), 611 (100), 615 (81), 618 (58), 622 (27), 649 (9), 681 (48), 684 (27), 690 (17), 695 (17), 700 (31), 704 nm (32); luminescence quantum yield (rhodamine 6G standard): *Φ*=0.32; HRMS (ESI+): *m*/*z* (%): 806.1583 [*M*+H]^+^ (85), 808.1598 [*M*+H]^+^ (100), 828.1401 [*M*+Na]^+^ (17), 830.1415 [*M*+Na]^+^ (20), 838.1843 [*M*+MeOH+H]^+^ (32), 840.1858 [*M*+MeOH+H]^+^ (35), 862.1678 [*M*+MeOH+K]^+^ (10) (C_33_H_32_N_7_O_8_Eu requires 806.1584 [*M*+H]^+^, 808.1597 [*M*+H]^+^, 828.1403 [*M*+Na]^+^, 830.1417 [*M*+Na]^+^, 838.1846 [*M*+MeOH+H]^+^, 840.1860 [*M*+MeOH+H]^+^, 862.1679 [*M*+MeOH+H]^+^).

Terbium(III) 2,2′‐[8‐(3‐Amino‐1‐carboxylatopropyl)‐5,8,11‐triaza‐1,2,3(2,6)‐tripyridinacyclododecaphane‐5,11‐diyl]diacetate (**13**‐Tb): See the general procedure for the preparation. UV/Vis (H_2_O): *λ*_max_ (*ϵ*)=235 (14 100), 283 (8100), 292 (11 300), 335 nm (10 700 mol^−1^ dm^3^ cm^−1^); MS (ESI+): *m*/*z* (%): 734.2 [*M*+H]^+^ (100), 756.2 [*M*+Na]^+^ (50), 772.1 [*M*+K]^+^ (30).

Terbium(III) 2,2′‐{8‐[3‐(*N*‐Maleimido)‐1‐carboxylatopropyl]‐5,8,11‐triaza‐1,2,3(2,6)‐tripyridinacyclododecaphane‐5,11‐diyl}diacetate (**14**‐Tb): Maleimide **14**‐Tb was prepared according to the procedure outlined for **14**‐Eu with **13**‐Tb (1.4 mg, 1.9 μmol). HPLC (method A): *t*_R_=19.0 min. Pure product **14**‐Tb (1.4 mg, 1.7 μmol, 89 %) was isolated. UV/Vis (HBS 7.3): *λ*_max_ (*ϵ*)=236 (14 300), 283 (8600), 292 (12 100), 335 nm (11 500 mol^−1^ dm^3^ cm^−1^); luminescence (HBS 7.3, *λ*_exc_=335 nm): *λ*_em_ (%)=486 (28), 501 (10), 541 (100), 546 (78), 549 (51), 553 (28), 583 (29), 588 (18), 620 (15), 624 nm (12); luminescence quantum yield (rhodamine 6G standard): *Φ*=0.18; HRMS (ESI+): *m*/*z* (%): 814.1636 [*M*+H]^+^ (70), 836.1458 [*M*+Na]^+^ (15), 846.1897 [*M*+MeOH+H]^+^ (100), 868.1716 [*M*+MeOH+Na]^+^ (25) (C_33_H_32_N_7_O_8_Tb requires 814.1639 [*M*+H]^+^, 836.1458 [*M*+Na]^+^, 846.1901 [*M*+CH_3_OH+H]^+^, 868.1720 [*M*+CH_3_OH+Na]^+^).

*tert*‐Butyloxycarbonyl‐aza‐glycine 4‐Nitrophenyl Ester (**15**): Boc‐hydrazine (251 mg, 1.9 mmol) was dissolved in dry THF (2.0 mL) and nitrophenyl chloroformate (191 mg, 0.95 mmol, 0.5 equiv) was added in portions. The solution was stirred overnight, and the precipitate was filtered off. The filtrate was dissolved in CHCl_3_ and extracted with 100 mm phosphate buffer pH 6.0 (2×) and 100 mm phosphate buffer pH 7.5 (sat. with NaCl) for as often as needed until the buffer phase no longer turned yellow. The organic solution was dried with Na_2_SO_4_ and concentrated under vacuum to give **15** (320 mg, 908 μmol, 96 %). ^1^H NMR (300 MHz, CDCl_3_, 25 °C, Me_4_Si): *δ*=1.50 (s, 9 H, C(C*H*_3_)_3_), 6.51 (br s, 1 H, NH), 7.11 (br s, 1 H, NH), 7.36 (d, ^3^ *J*_H,H_=8.9 Hz, 2 H, *ortho* CH), 8.26 ppm (d, ^3^ *J*_H,H_=9.0 Hz, 2 H, *meta* CH).

2,2′‐(8‐{3‐[2‐(*tert*‐Butoxycarbonyl)hydrazine‐1‐carboxamido]‐1‐carboxypropyl}‐5,8,11‐triaza‐1,2,3(2,6)‐tripyridinacyclododecaphane‐5,11‐diyl)diacetic acid (**16**): A solution of **15** (3.4 mg, 10 μmol, 2 equiv) in dry DMF (120 μL) was added to dry **13** (5.0 mg, 4.8 μmol, dried by evaporation with dry toluene, 3×). DIPEA (13.5 μL, 77 μmol, 16 equiv) was added, and the solution was stirred under an argon atmosphere at RT overnight. Subsequently, the mixture was frozen in liquid nitrogen and attached to a liquid‐nitrogen‐cooled cold trap, and the solvent was removed with stirring at 10–100 Pa. The residue was dissolved in 0.1 % acetic acid/5 % MeOH in water (1 mL). This solution was loaded onto the HPLC column, and the collected fractions were evaporated under vacuum to dryness. HPLC (method B): *t*_R_=15.5 min. Pure product **16** (4.1 mg, 4.5 μmol, 93 %) was isolated. ^1^H NMR (300 MHz, [D_4_]methanol, 25 °C, Me_4_Si): *δ*=1.46 (s, 9 H, C(C*H*_3_)_3_), 1.56–1.73 (m, 1 H, β‐CH^I^_2_), 1.74–1.89 (m, 1 H, β‐CH^II^_2_), 2.91–3.14 (m, 2 H, γ‐CH_2_), 3.14–3.45 (m, 4 H, N‐CH_2_‐C*H*_2_‐N‐C*H*_2_‐CH_2_‐N), 3.45–3.54(m, 1 H, α‐CH), 3.54–3.70 (m, 4 H, N‐C*H*_2_‐CH_2_‐N‐CH_2_‐C*H*_2_‐N), 3.76 (br s, 4 H, N‐C*H*_2_‐COOH), 4.73 (br s, 4 H, N‐C*H*_2_(*meso*)‐C‐N), 7.61 (d, ^3^ *J*_H,H_=6.9 Hz, 2 H, CH of 5 and 5′′ in terpyridine), 8.07–8.21 ppm (m, 7 H, CH of 3, 4, 3′, 4′, 5′, 3′′ and 4′′ in terpyridine); ^13^C NMR (75 MHz, [D_4_]methanol, 25 °C, Me_4_Si): *δ*=27.1 (3 C, C(*C*H_3_)_3_), 29.4 (1 C, β‐CH_2_), 36.1 (1 C, γ‐CH_2_), 46.8 (2 C, CH_2_, N‐CH_2_‐*C*H_2_‐N‐*C*H_2_‐CH_2_‐N), 53.3 (2 C, N‐*C*H_2_‐CH_2_‐N‐CH_2_‐*C*H_2_‐N), 56.7 (2 C, N‐*C*H_2_‐COOH), 58.0 (2 C, N‐*C*H_2_(*meso*)‐C‐N), 59.9 (1 C, α‐CH), 122.4 (1 C, CH of 3, 4, 3′, 4′, 5′, 3′′ or 4′′ in terpyridine), 122.6 (1 C, CH of 3, 4, 3′, 4′, 5′, 3′′ or 4′′ in terpyridine), 123.8 (2 C, CH of 5 and 5′′ in terpyridine), 138.8 (C, CH of 3, 4, 3′, 4′, 5′, 3′′ or 4′′ in terpyridine), 139.1 ppm (C, CH of 3, 4, 3′, 4′, 5′, 3′′ or 4′′ in terpyridine); MS (ESI+): *m*/*z* (%): 736.5 [*M*+H]^+^ (100), 758.4 [*M*+Na]^+^ (40), 774.3 [*M*+K]^+^ (5), 734.3 [*M*−H]^−^ (100), 756.1 [*M*+N−2 H]^−^ (10).

2,2′‐{8‐[1‐Carboxy‐3‐(hydrazinecarboxamido)propyl]‐5,8,11‐triaza‐1,2,3(2,6)‐tripyridinacyclododecaphane‐5,11‐diyl}diacetic acid (**17**): Dry **16** (4.1 mg) was treated overnight in dry CH_2_Cl_2_ (190 μL) and TFA (190 μL) under an argon atmosphere to remove the Boc group. After the reaction was finished, toluene was added to the solution. The residue was evaporated from toluene (3×), MeOH (3×), and Milli‐Q water (1×) to give **17** (3.9 mg, 4.5 μmol, 100 %). ^1^H NMR (300 MHz, D_2_O, 25 °C): *δ*=1.84–2.00 (m, 2 H, β‐CH_2_), 3.05–3.12 (m, 2 H, γ‐CH_2_), 3.17–3.33 (m, 4 H, N‐CH_2_‐C*H*_2_‐N‐C*H*_2_‐CH_2_‐N), 3.34–3.38 (m, 1 H, α‐CH), 3.61–3.77 (m, 8 H, N‐C*H*_2_‐CH_2_‐N‐CH_2_‐C*H*_2_‐N and N‐C*H*_2_‐COOH), 4.76 (br s, 4 H, N‐C*H*_2_(*meso*)‐C‐N), 7.80 (d, ^3^ *J*_H,H_=7.7 Hz, 2 H, CH of 5 and 5′′ in terpyridine), 8.26 (t, ^3^ *J*_H,H_=7.9 Hz, 2 H, CH of 4 and 4′′ in terpyridine), 8.41 (d, ^3^ *J*_H,H_=8.0 Hz, 2 H, CH of 3 and 3′′ in terpyridine), 8.63 (d, ^3^ *J*_H,H_=7.6 Hz, 2 H, CH of 3′ and 5′ in terpyridine), 8.73 ppm (t, ^3^ *J*_H,H_=8.0 Hz, 1 H, CH of 4′ in terpyridine); ^13^C NMR (75 MHz, D_2_O, 25 °C): *δ*=27.0 (1 C, β‐CH_2_), 37.5 (1 C, γ‐CH_2_), 47.3 (2 C, N‐CH_2_‐*C*H_2_‐N‐*C*H_2_‐CH_2_‐N), 53.4 (2 C, N‐*C*H_2_‐CH_2_‐N‐CH_2_‐*C*H_2_‐N), 56.1 (2 C, N‐*C*H_2_‐COOH), 57.2 (2 C, N‐*C*H_2_(*meso*)‐C‐N), 61.5 (1 C, α‐CH), 124.2 (2 C, CH of 3 and 3′′ in terpyridine), 125.5 (2 C, CH of 3′ and 5′ in terpyridine), 128.1 (2 C, CH of 5 and 5′′ in terpyridine), 141.4 (2 C, CH of 4 and 4′′ in terpyridine), 147.0 ppm (1 C, CH of 4′ in terpyridine); MS (ESI+): *m*/*z* (%):636.2 [*M*+H]^+^ (80), 658.3 [*M*+Na]^+^ (100), 674.2 [*M*+K]^+^ (30), 690.3 [*M*+MeOH+Na]^+^ (35), 712.4 [*M*−H+2 K]^+^ (35).

Europium(III) 2,2′‐{8‐[1‐Carboxylato‐3‐(hydrazinecarboxamido)propyl]‐5,8,11‐triaza‐1,2,3(2,6)‐tripyridinacyclododecaphane‐5,11‐diyl}diacetate (**17**‐Eu): See the general procedure for lanthanide complex formation for the preparation. UV/Vis (HBS 7.3): *λ*_max_ (*ϵ*)=235 (13 900), 283 (7560), 292 (10 430), 336 nm (10 430 mol^−1^ dm^3^ cm^−1^); luminescence (HBS 7.3, *λ*_exc_=335 nm): *λ*_em_=578, 586, 594, 610, 615, 618, 622, 647, 679, 688, 693, 698, 702 nm; HRMS (ESI+): *m*/*z* (%): 784.1850 [*M*+H]^+^ (90), 786.1864 [*M*+H]^+^ (100), 806.1669 [*M*+Na]^+^ (21), 808.1682 [*M*+Na]^+^ (23), 822.1407 [*M*+K]^+^ (10), 824.1420 [*M*+K]^+^ (11) (C_30_H_34_N_9_O_7_Eu requires 784.1852 [*M*+H]^+^, 786.1866 [*M*+H]^+^, 806.1672 [*M*+Na]^+^, 808.1686 [*M*+Na]^+^, 822.1411 [*M*+K]^+^, 824.1425 [*M*+K]^+^).

BSA labeling: A solution of **14**‐Eu (0.1 mg, 124 nmol) in MeOH/H_2_O (1:1, 200 μL) was transferred to a 1.5 mL vial commonly used in autosamplers and taken to dryness under reduced pressure. BSA (13.2 mg, 100 nmol, 0.81 equiv) was separately dissolved in HBS 7.3 containing 1 mm EDTA (0.5 mL), and foam formation was avoided by stirring the solution slowly until complete solvation. The BSA solution was added to the vial containing dry **14**‐Eu, and subsequently, the air was displaced by argon and the vial was closed with a crimp cap. The vial was cautiously swirled from time to time and was left to stand overnight at room temperature. Labeled BSA was purified by size‐exclusion chromatography in HBS 7.3 with a Superdex 200 increase column (10 mm×300 mm, GE Healthcare, flow rate=0.5 mL min^−1^), while collecting 0.5 mL fractions. The product was detected by its absorbance at *λ*=280 and 330 nm (for the chromatogram see Figure S69).

## Conflict of interest

*The authors declare no conflict of interest*.

## Supporting information

As a service to our authors and readers, this journal provides supporting information supplied by the authors. Such materials are peer reviewed and may be re‐organized for online delivery, but are not copy‐edited or typeset. Technical support issues arising from supporting information (other than missing files) should be addressed to the authors.

Supplementary
