# Supplementary material for: Stable Europium(III) Complexes with Short Linkers for Site‐Specific Labeling of Biomolecules
Source: ChemistryOpen. 2017 Sep 4;6(6):721–32. doi: 10.1002/open.201700122 (PMC5715356; doi:10.1002/open.201700122)
Supplement: Supplementary file 1 — Supplementary [file OPEN-6-721-s001.pdf]

ChemistryOPEN

Supporting Information

© 2017 The Authors. Published by Wiley-VCH Verlag GmbH & Co. KGaA, Weinheim

Stable Europium(III) Complexes with Short Linkers for Site-Specific Labeling of Biomolecules

Felix Faschinger,[a] Martin Ertl,[b] Mirjam Zimmermann,[a] Andreas Horner,[a] Markus Himmelsbach,[c] Wolfgang Schöfberger,[d] Günther Knör,[b] and Hermann J. Gruber*[a]

open_201700122_sm_miscellaneous_information.pdf

Author Contributions

F.F. Conceptualization: Equal; Data curation: Lead; Investigation: Lead; Methodology: Lead; Project administration: Supporting; Writing – original draft: Lead; Writing – review & editing: Equal
M.E. Data curation: Supporting; Investigation: Supporting; Writing – review & editing: Supporting
M.Z. Conceptualization: Supporting; Data curation: Supporting; Methodology: Supporting
A.H. Data curation: Supporting; Methodology: Supporting; Resources: Supporting
M.H. Data curation: Supporting; Investigation: Supporting; Resources: Supporting; Writing – review & editing: Supporting
W.S. Data curation: Supporting; Investigation: Supporting; Resources: Supporting; Writing – review & editing: Supporting
G.K. Conceptualization: Supporting; Resources: Supporting; Writing – review & editing: Supporting
H.G. Conceptualization: Equal; Funding acquisition: Lead; Investigation: Supporting; Methodology: Supporting; Project administration: Lead; Resources: Lead; Supervision: Lead; Writing – original draft: Supporting; Writing – review & editing: Equal.

Supporting information

Estimation of the labeling efficiency of BSA:
The coupling reaction of **14-Eu** with BSA results in almost complete labeling of all available cysteine residues. It is known that on average every second BSA molecule possesses a reactive sulfhydryl-group.[1] The extinction coefficient of free **14-Eu** at 335 nm was determined to be 11900 M^{-1} cm^{-1}. The formal extinction coefficient of labeled BSA at 280 nm is composed of the extinction coefficient of two BSA molecules (2 × 44300 M^{-1} cm^{-1})[2] and the absorbance of one **14-Eu** at 280 nm (8200 M^{-1} cm^{-1}), which sums up to 96800 M^{-1} cm^{-1}. This theoretical extinction coefficient is valid for 100% labeling of the available free 0.5 thiol groups per BSA molecule; it is shown in Table S1. In this maximally labeled complex the ratio of ε(335 nm)/ε(280 nm) has the value 0.123. In the actual sample of **14-Eu**-labeled BSA, the ratio of ε(335 nm)/ε(280 nm) was 0.113, which means that approx. 91% of the available thiol groups of BSA were labeled with **14-Eu**.

Table S1: Extinction of the **14-Eu** labeled BSA at 335 and 280 nm was used to estimate the labeling efficiency.

	ε(335 nm) [M^{-1} cm^{-1}]	ε(280 nm) [M^{-1} cm^{-1}]	$\frac{\varepsilon(335\ \text{nm})}{\varepsilon(280\ \text{nm})}$	Labeling efficiency [%]
**Theory**	11900	96800	0.123	100
**Measured**	11900	105800	0.113	91

Determination of the luminescence quantum yield:

The quantum yields were calculated with the following formula:[3]

$$\phi_D = \phi_S \cdot \frac{E_D \cdot A_s}{A_D \cdot E_s} \cdot \left(\frac{n_D}{n_S}\right)^2$$

ϕ_D is the quantum yield of the sample dye, ϕ_S is the quantum yield of the reference standard, E_D/A_D is the slope of emission intensity area vs. the absorption of the sample dye at 335 nm and E_S/A_S is the corresponding slope of the reference standard, n_D = 1.334 is the refractive index of HBS 7.3 buffer used to dissolve the sample dye and n_S = 1.3638 is the refractive index of ethanol used to dissolve the reference standard.

Table S2: Determination of the luminescence quantum yield by relative method with rhodamine 6G as standard.[3,4]

Compound	Absorbance *A* at 335 nm [A.U.]	Emission area *E* [A.U.]	Slope *E*/*A*	Quantum yield ϕ [%]
**Rhodamine 6G**	0.0081	$8.09 \cdot 10^7$	$6.97 \cdot 10^9$	94.0±3.8
	0.0060	$6.71 \cdot 10^7$		
	0.0037	$4.82 \cdot 10^7$		
	0.0016	$3.28 \cdot 10^7$		
	$1.28 \cdot 10^{-4}$	$2.72 \cdot 10^7$		
**14-Eu**	0.060	$1.60 \cdot 10^8$	$2.50 \cdot 10^9$	32.3±2.0
	0.045	$1.25 \cdot 10^8$		
	0.040	$1.09 \cdot 10^8$		
	0.030	$8.50 \cdot 10^7$		
	0.022	$6.84 \cdot 10^7$		
	0.016	$5.23 \cdot 10^7$		
	0.0079	$2.92 \cdot 10^7$		
	0.0070	$2.50 \cdot 10^7$		
**14-Tb**	0.053	$7.09 \cdot 10^7$	$1.42 \cdot 10^9$	18.4±2.4
	0.038	$5.10 \cdot 10^7$		
	0.027	$3.70 \cdot 10^7$		
	0.023	$2.90 \cdot 10^7$		
	0.018	$2.10 \cdot 10^7$		
	0.016	$1.75 \cdot 10^7$		
	0.013	$1.47 \cdot 10^7$		
**14-Eu labeled BSA**	0.032	$8.25 \cdot 10^7$	$2.24 \cdot 10^9$	28.8±2.3
	0.024	$6.55 \cdot 10^7$		
	0.015	$4.44 \cdot 10^7$		
	0.0095	$3.43 \cdot 10^7$		
	0.0069	$2.62 \cdot 10^7$		
	0.0033	$1.67 \cdot 10^7$		

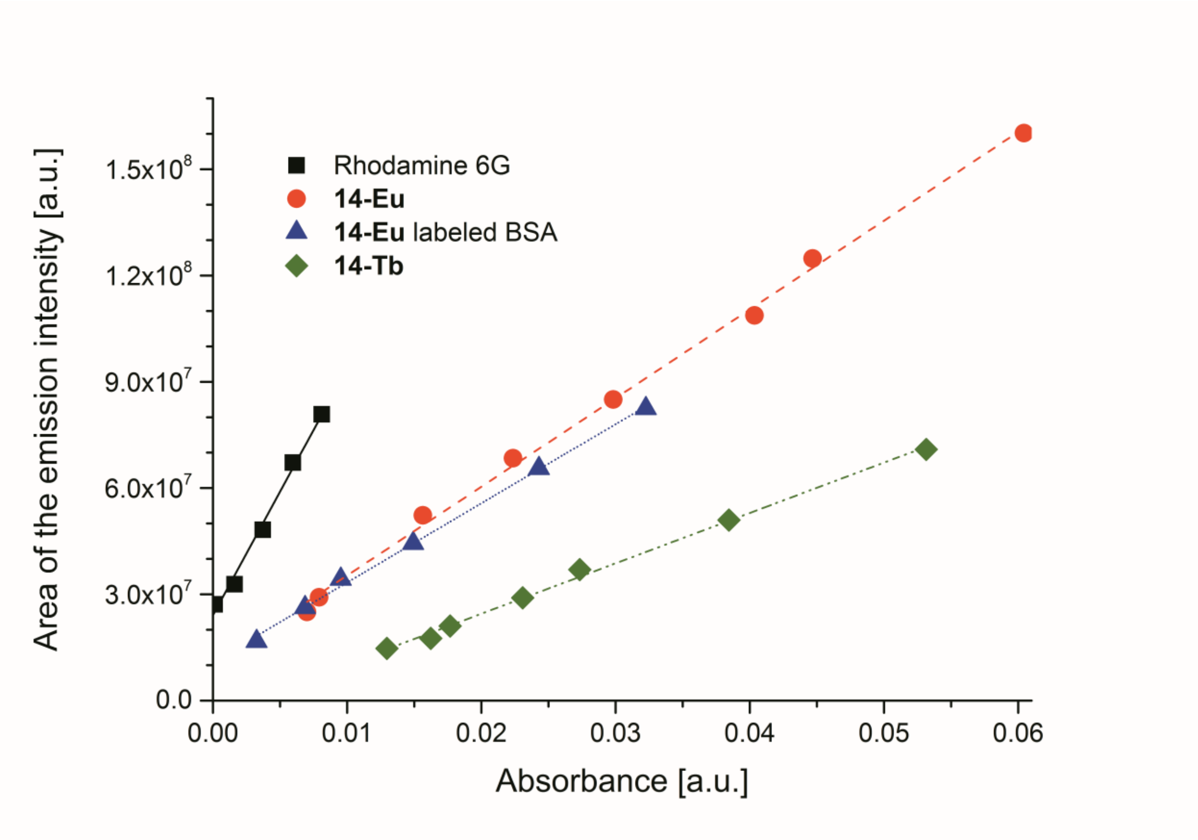

Figure S1: Measurements of luminescence quantum yield by relative method, using rhodamine 6G as standard.[3,4]

Determination of the number of water molecules in the inner coordination-sphere:

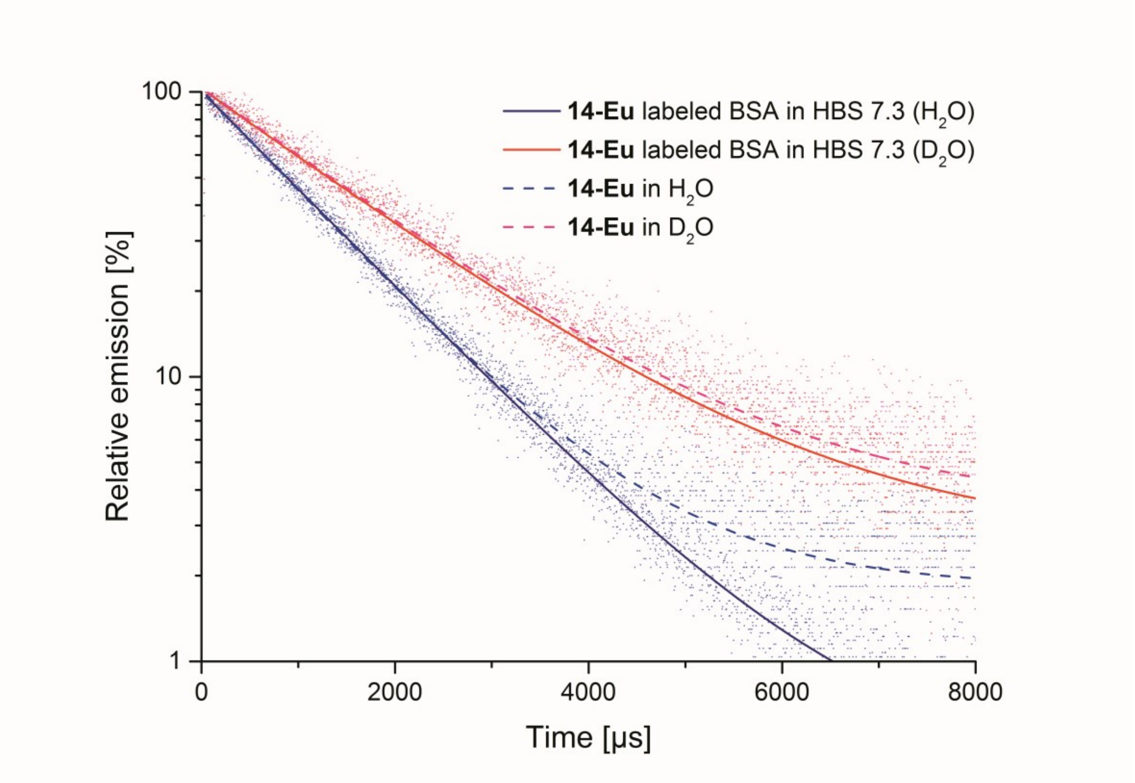

Figure S2: Lifetime measurement of **14-Eu**-labeled BSA in HBS 7.3 buffer (dark blue solid line) and in deuterated HBS 7.3 buffer (red solid line). Essentially the same lifetimes were observed for **14-Eu** in water (blue dashed line) and in deuterated water (pink dashed line).

Eu-complex stability in presence of competitors:

In Figure S3 the luminescence intensity of **17-Eu** (3 µM) in HBS 7.3 was monitored in the presence of competitors for more than 24 h. The luminescence intensity was not influenced upon addition of 2 mM EDTA, 2 mM EGTA, 2 mM EGTA-Ca-buffer, 10 µM zinc(II) or 200 µM zinc(II). This compares well with the observations that neither EDTA nor EGTA can extract the Eu-ion from the chelate, and that zinc(II) or calcium(II) are not able to cause transmetalation. Control experiments were performed with 3 µM $EuCl_3$ in the identical HBS 7.3 buffer and 2 mM EDTA or 2 mM EGTA, in order to ensure that neither Eu-EDTA, Eu-EGTA nor any other buffer contents could cause luminescence, which would lead to misinterpretation of the data.

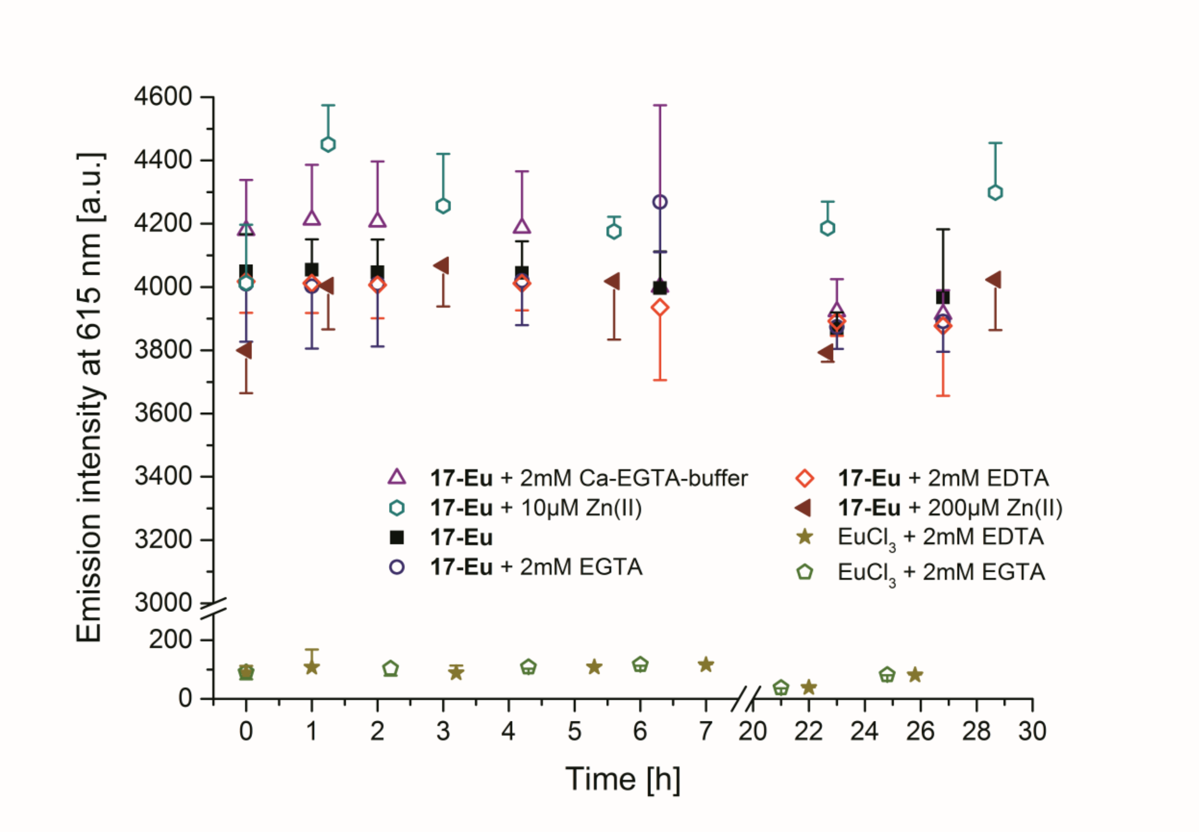

Figure S3: The luminescence intensity of **17-Eu** (3 µM) and **17-Eu** in presence of 2 mM EDTA, 2 mM EGTA, 2 mM Ca-EGTA buffer, 10 µM Zn^{II} and 200 µM Zn^{II} was monitored for more than 24 h. As control experiment the luminescence intensity of 3 µM europium(III) chloride in HBS 7.3 buffer with 2 mM EDTA or 2 mM EGTA was monitored, in order to control if luminescence from buffer components can be observed.

Influence of nucleotides on the luminescence properties of 14-Eu labeled BSA: The effect of nucleotide present in the solution was strongly dependent on the wavelength used to excite the Eu-complex. For measurements in a fluorimeter cuvette, the samples were excited at 335 nm (slit 10 nm). Hereby the nucleotides (or a common impurity of the nucleotides) caused a significant 335 nm absorption at a nucleotide concentration of approx. 25 mᴍ (see Figure S6). When monitoring the luminescence intensity of **14-Eu** labeled BSA incubated with nucleotide solutions over a period of 10 min, the intensity was not changed (see Figure S4). Figure S4B displays the luminescence intensity corrected for the dilution which had been caused by the addition of the nucleotide stock solution. In this case a prominent filter-effect was observed for GTP only. This experiment allows the conclusion, that the nucleotides do not perturb the emission of the Eu-complex when corrected for the "filter effect" (Figure 5B and Figure S6).

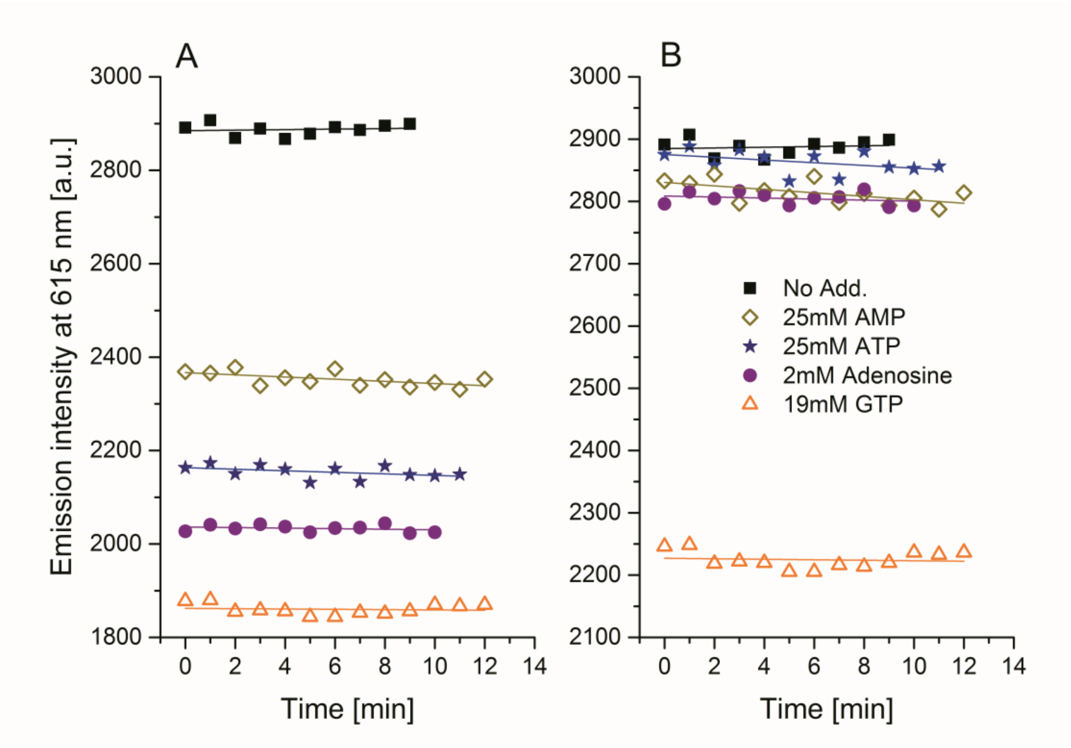

Figure S4: Stability of **14-Eu** labeled BSA in the presence of different nucleotides or adenosine. The luminescence intensity was monitored at 1 min intervals. A) The emission intensities as measured is shown. B) The data from panel A were corrected for the dilution caused by the addition of the 100 mᴍ stock solution (the stock solution of adenosine was 10 mᴍ). The reduced emission intensity of **14-Eu** in presence of 19 mᴍ GTP in panel B is the consequence of much higher absorbance of GTP or a common impurity of GTP at 335 nm, as shown in Figure S6.

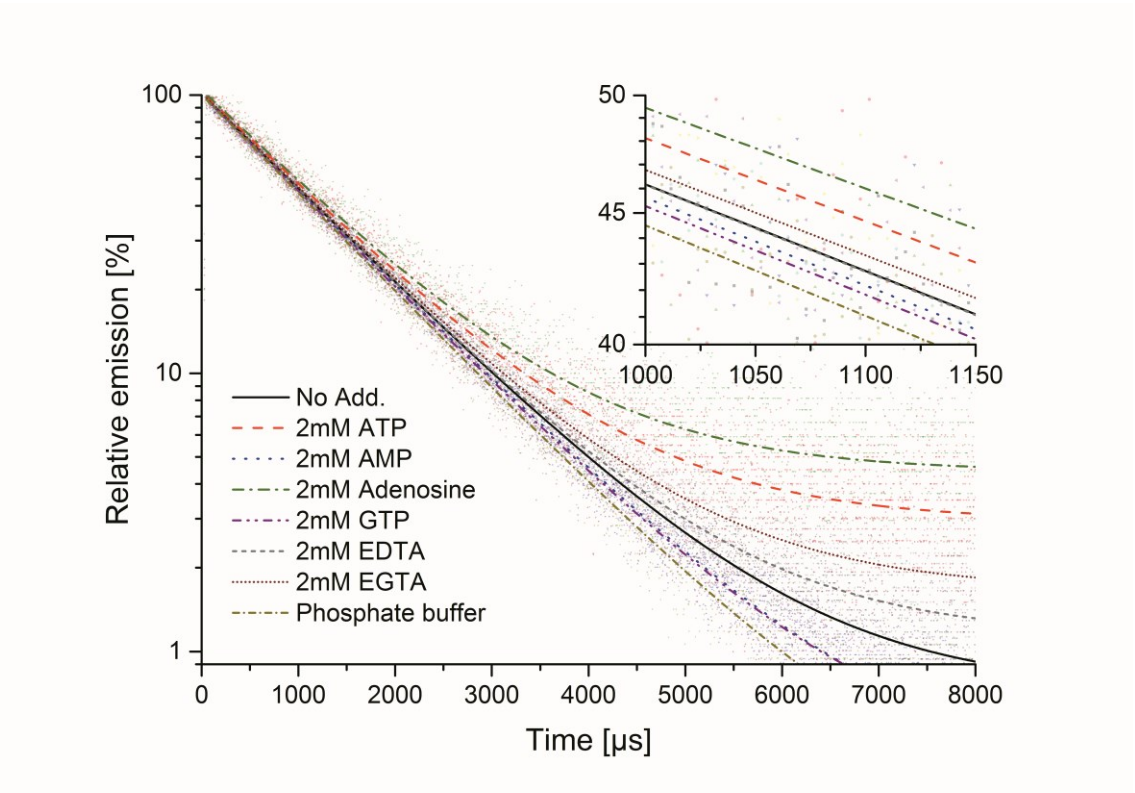

Figure S5: Lifetimes of **14-Eu**–labeled BSA in presence of nucleotides or competitive chelators like EDTA and EGTA. The measurements were performed with 3 µм europium label in HBS 7.3 buffer (except for the test in phosphate buffer). All time courses exhibited a mono-exponential decay and were fitted to the equation $I = I_0 + I_1 \cdot e^{-(t/\tau)}$ which takes into account a constant background (resulting from background light and detector noise). The insert illustrates the nearly parallel nature of the curves at levels which are much higher than the background, reflecting essentially equal lifetimes.

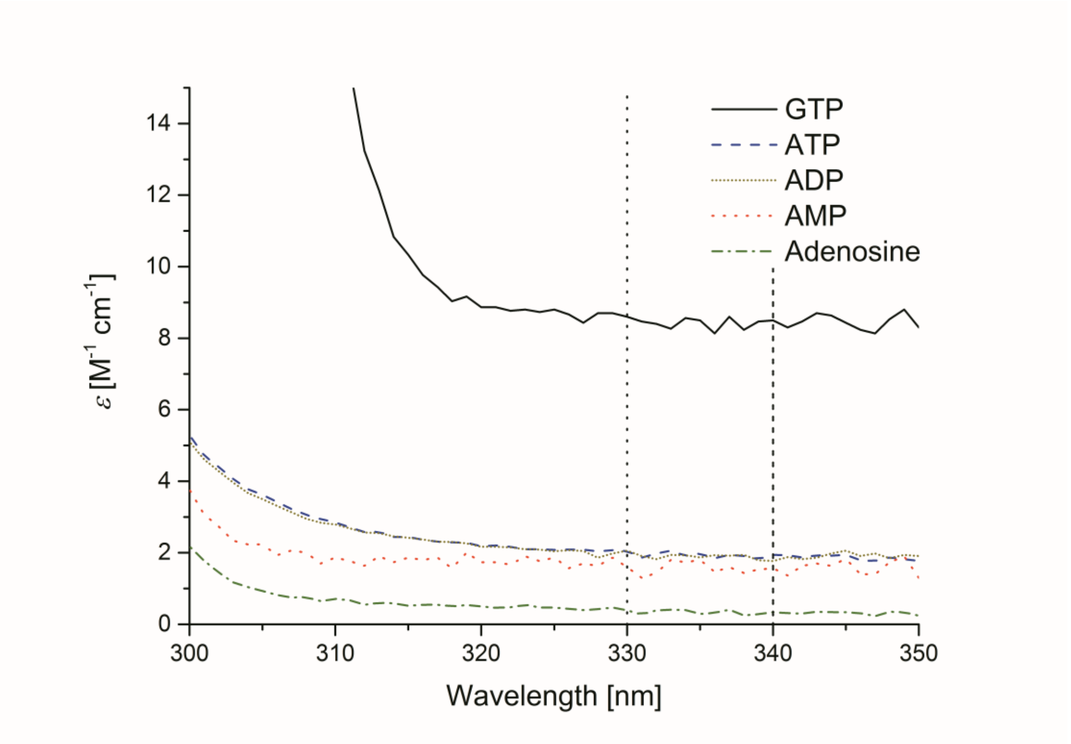

Figure S6: Extinction coefficient determined from approx. 10 mм nucleotide or adenosine solutions in HBS 7.3 buffer. The window between 330 and 340 nm was used for **14/17-Eu** excitation in the stability tests.

Material and methods

Lifetime measurements: The time-resolved measurements were performed on a home-build setup based on a Nikon Diaphot 300 (Tokyo, Japan). Excitation was performed with a 266 nm Nd:YAG laser from VM-TIM LS-2132UTF (Jena, Germany). Monochromaticity was ensured with two FGUV11 UV-bandpass filters (Thorlabs, Dachau, Germany) and the laser-beam was redirected into the microscope body with two PF10-03-F01 mirrors (Thorlabs). The beam was directed with a z266rdc dichroic mirror Chroma (Bellows Falls, USA) through a Partec (Sysmex, Kobe, Japan) 40x1.25 glycerol immersion quartz objective, to focus the excitation light from the bottom onto the glass slide carrying the sample-solution. The emitted light was collected by the objective, passed through the dichroic mirror and an HQ465lp longpass filter (Chroma). The emitted light is focused onto the detector by a LA1805-A lens (Thorlabs) with focal length of 30 mm. A Perkin Elmer SPCM-AQRH gated single photon avalanche diode (Waltham, USA) which was controlled by a PicoQuant DSN 101 power supply with integrated counter (Berlin, Germany) was used for photon detection. The system was triggered and decay curves were recorded by a PicoQuant NanoHARP 250. The trigger signal was looped for 30 µs before gating the detector-photodiode, in order to prevent detection of short lived components. The measurements were performed with a droplet of sample solution (typically 50 – 100 µL with a lanthanide complex concentration of approx. 3 µм) on a glass slide. The obtained time courses were the sum of 600 single measurements, recorded in 0.1 s intervals. The total time per single measurement was 30 ms with 7325 data points detected at 4.096 µs intervals. The decay curves were fitted with OriginPro 9.1 (OriginLab Corporation, USA) by applying a statistically weighted least square fit of an exponential decay function ($I=I_0 + \sum_i I_i\, e^{-(t/\tau_i)}$) with *i* (the number of different decay times; ranging from 1 - 2). The resulting decay constants τ_i represent the lifetimes measured for the lanthanide complex in the sample solution. I_0 represents the time-independent background light.

Synthesis of 2,2':6',2''-terpyridine 1,1''-di-*N*-oxide (1): 2,2′:6′,2″-terpyridine (30 mg, 125 µmol) was placed in a round bottom flask and DCM (2.25 mL) was added to dissolve the solid. While stirring mCPBA (162.3 mg, 77 w/w %, 724 µmol, 5.8 equiv.) was added. The solution was stirred for 24 h at room temperature. The resulting mixture was extracted four times with 10% Na_2CO_3 (750 µL). The combined organic phases were dried with Na_2SO_4 and concentrated to dryness under reduced pressure. The crude solid was washed with acetonitrile, filtered and redissolved in DCM. The solvent was evaporated under reduced pressure to obtain product **1** (15.6 mg, 58.8 µmol, 47%). ^{1}H NMR (300 MHz, $CDCl_3$, 25°C, TMS): δ=8.94 (d, ^3J(H,H)=8.0 Hz, 2H, CH of 3' and 5'), 8.34 (d, ^3J(H,H)=5.9 Hz, 2H, CH of 6 and 6''), 8.20 (dd, ^3J(H,H)=7.9 Hz, ^4J(H,H)=2.1 Hz, 2H, CH of 3 and 3''), 7.98 (t, ^3J(H,H)=8.0 Hz, 1H, CH of 4'), 7.38 (dt, ^3J(H,H)=7.7 Hz, ^4J(H,H)=1.1 Hz, 2H, CH of 5 and 5''), 7.30 (dt, ^3J(H,H)=6.9 Hz, ^4J(H,H)=2.0 Hz, 2H, CH of 4 and 4''); HRMS (ESI+): *m/z* (%): 266.0927 $[M+H]^+$ (100), 288.0744 $[M+Na]^+$ (15), 304.0483 $[M+K]^+$ (1) ($C_{15}H_{11}N_3O_2$ requires 266.0924 $[M+H]^+$, 288.0743 $[M+Na]^+$, 304.0483 $[M+K]^+$).

Synthesis of 2,2':6',2''-terpyridine-6,6''-dicarbonitrile (2): 1 (15.6 mg, 58.8 µmol) was dissolved in DCM (706 µL) and trimethylsilyl cyanide (77 µL, 588 µmol, 10 equiv.) was added. The solution was stirred for 20 min at room temperature and subsequently PhCOCl (28 µL, 235 µmol, 4 equiv.) was added dropwise during a time period of 20 min. The mixture was stirred overnight at room temperature. The

reaction mixture was concentrated to half of the volume, 10% K_2CO_3 (1.4 mL) was added and the suspension was stirred for 1 h at r.t. The resulting precipitate was filtered and washed with water and cold DCM. The remaining solid was dried in vacuum to obtain **2** (11.7 mg, 41.3 µmol, 70%). ^{1}H NMR (300 MHz, $CDCl_3$, 25°C, TMS): δ=7.75 (dd, ^3J(H,H)=7.6 Hz, ^4J(H,H)=1.0 Hz, 2H, CH of 5 and 5''), 8.01 (t, ^3J(H,H)=7.9 Hz, 2H, CH of 4 and 4''), 8.05 (t, ^3J(H,H)=7.9 Hz, 1H, CH of 4'), 8.58 (d, ^3J(H,H)=7.9 Hz, 2H, CH of 3' and 5'), 8.82 (dd, ^3J(H,H)=8.1 Hz, ^4J(H,H)=1.0 Hz, 2H, CH of 3 and 3''); ^{13}C NMR (75 MHz, $CDCl_3$, 25°C, TMS): δ=117.4 (2 C, C^6-**C**N and $C^{6''}$-**C**N), 122.8 (2 C, CH of 3+3'' or 5+5'' or 3'+5'), 124.3 (2 C, CH of 3+3'' or 5+5'' or 3'+5'), 128.5 (2 C, CH of 3+3'' or 5+5'' or 3'+5'), 133.5 (2 C, C of 6+6''), 138.1 (2 C, CH of 4+4''), 138.7 (1 C, CH of 4'), 153.7 (2 C, C of 2+2'' or 2'+5'), 157.5 (2 C, C of 2+2'' or 2'+5'); HRMS (ESI+): *m/z* (%): 284.0933 $[M+H]^+$ (100), 306.0750 $[M+Na]^+$(20) ($C_{17}H_9N_5$ requires 284.0931 $[M+H]^+$, 306.0750 $[M+Na]^+$).

Synthesis of dimethyl 2,2':6',2''-terpyridine-6,6''-dicarboxylate (3): 2 (145 mg, 512 µmol) was dissolved in water (2.3 mL), acetic acid (2.3 mL), and conc. sulfuric acid (512 µL). The mixture was heated to 90 – 100°C and stirred for 24 h. Afterwards the solution was poured into ice-water (20.5 mL) and the precipitate was filtered off. The solid was washed with cold water and acetonitrile. The carboxylic acid was dried 10 – 100 Pa. MeOH (16 mL) was cooled in an ice bath and thionyl chloride (390 µL, 5.3 mmol, 10 equiv.) were added dropwise. The solution was stirred for 15 min at room temperature and was then added to the solid carboxylic acid (170 mg, 529 µmol). The mixture was heated to reflux for 18 h and subsequently the solvent was removed under reduced pressure. The solid was dissolved in $CHCl_3$ (27 mL) and washed three times with 5% $NaHCO_3$ (13 mL). The organic phase was dried with Na_2SO_4 and the solvent was removed in vacuum. The crude product was recrystallized from toluene (28 mL) to obtain **3** (126 mg, 445 µmol, 84%). ^{1}H NMR (300 MHz, $CDCl_3$, 25°C, TMS): δ=4.06 (s, 6H, COOC**H$_3$**), 8.02 (t, ^3J(H,H)=7.8 Hz, 3H, CH of 4, 4' and 4''), 8.18 (dd, ^3J(H,H)=7.7 Hz, ^4J(H,H)=1.1 Hz, 2H, CH of 5 and 5''), 8.63 (d, ^3J(H,H)=7.8 Hz, 2H, CH of 3' and 5'), 8.81 (dd, ^3J(H,H)=7.9 Hz, ^4J(H,H)=1.1 Hz, 2H, CH of 3 and 3''); ^{13}C NMR (75 MHz, $CDCl_3$, 25°C, TMS): δ=53.0 (2 C, COO**CH$_3$**), 122.3 (2 C, CH of 3+3'' or 5+5'' or 3'+5'), 124.5 (2 C, CH of 3+3'' or 5+5'' or 3'+5'), 125.3 (2 C, CH of 3+3'' or 5+5'' or 3'+5'), 138.0 (2 C, CH of 4+4''), 138.4 (1 C, CH of 4'), 147.8 (2 C, C of 6+6''), 154.7 (2 C, C of 2+2'' or 2'+5'), 156.5 (2 C, C of 2+2'' or 2'+5'), 166.0 (2 C, **C**OOCH$_3$); HRMS (ESI+): *m/z* (%):350.1141 $[M+H]^+$ (100), 372.0955 $[M+Na]^+$ (20), 388.0695 $[M+K]^+$ (2), ($C_{19}H_{15}N_3O_4$ requires 350.1135 $[M+H]^+$, 372.0955 $[M+Na]^+$, 388.0694 $[M+K]^+$).

Synthesis of 6,6''-dihydroxymethyl-2,2':6',2''-terpyridine (4): 3 (120 mg, 344 µmol) was dissolved in dry EtOH (10 mL) and sodium borohydride (77 mg, 1.96 mmol, 5.7 equiv.) was added under argon atmosphere. The mixture was stirred for 3 h at r.t. and subsequently heated to reflux for 1 h. The organic solvent was removed under reduced pressure, sat. $NaHCO_3$ (4.9 mL) was added and the mixture was shortly heated to boiling. After cooling to r.t. the precipitate was filtered off and washed with water to obtain **4** (87 mg, 297 µmol, 86%). ^{1}H NMR (300 MHz, [D$_6$] DMSO, 25°C, TMS): δ=4.69 (s, 4H, **CH$_2$**-OH), 5.57 (vbrs, 2H, CH$_2$-**OH**), 7.58 (d, ^3J(H,H)=7,6 Hz, 2H, CH of 5 and 5''), 8.01 (t, ^3J(H,H)=7.8 Hz, 2H, CH of 4 and 4''), 8.08 (t, ^3J(H,H)=7.8 Hz, 1H, CH of 4'), 8.43 (d, ^3J(H,H)=7.8 Hz, 2H, CH of 3' and 5'), 8.49 (d, ^3J(H,H)=7.6 Hz, 2H, CH of 3 and 3''). ^{13}C NMR (75 MHz, [D$_6$] DMSO, 25°C, TMS): δ=79.2 (2 C, **CH$_2$**-OH), 118.8 (2 C, CH of 3+3'' or 5+5'' or 3'+5'), 120.6 (2 C, CH of 3+3'' or 5+5'' or 3'+5'), 120.7 (2 C, CH of 3+3'' or 5+5'' or 3'+5'), 137.8 (2 C,

CH of 4+4”), 138.4 (1 C, CH of 4’), 154.1 (2 C, C of 6+6”), 154.9 (2 C, C of 2+2” or 2’+5’), 161.7 (2 C, C of 2+2” or 2’+5’); HRMS (ESI+): *m/z* (%): 294.1232 $[M+H]^+$ (45), 316.1050 $[M+Na]^+$ (100), ($C_{17}H_{15}N_3O_2$ requires 294.1237 $[M+H]^+$, 316.1056 $[M+Na]^+$).

Synthesis of 6,6”-dibromomethyl-2,2’:6’,2”-terpyridine (5): 4 (137 mg, 467 µmol) and lithium bromide (44.6 mg, 46.7 µmol, 1.1 equiv.) were dissolved in dry DMF (5 mL) in an argon atmosphere. Phosphorus tribromide (213 µL, 2.27 mmol, 4.9 equiv.) was slowly added from a Hamilton™ syringe to the rigorously stirred mixture. Reaction controls were performed by TLC (silica gel; $CHCl_3$:MeOH = 8:2, R_f=0.73). After complete reaction (approx. 4 h) the mixture was frozen in liquid nitrogen, attached to a liquid nitrogen-cooled cold-trap, and DMF was evaporated at 10 – 100 Pa. The residue was dissolved in $CHCl_3$ (22 mL). The organic phase was washed twice with 5% $NaHCO_3$ (13 mL). The combined aqueous phase was re-extracted with $CHCl_3$ (13 mL) and finally the combined organic phases were washed with brine. The organic solution was dried with Na_2SO_4 and the solvent was removed under reduced pressure to obtain **5** (167 mg, 399 µmol, 86%). ^1H NMR (300 MHz, $CDCl_3$, 25°C, TMS): δ=4.66 (s, 4H, $\mathbf{CH_2}$-Br), 7.49 (dd, ^3J(H,H)=7.7 Hz, ^4J(H,H)=0.8 Hz, 2H, CH of 5 and 5”), 7.86 (t, ^3J(H,H)=7.8 Hz, 2H, CH of 4 and 4”), 7.96 (t, ^3J(H,H)=7.8 Hz, 1H, CH of 4’), 8.52 (d, ^3J(H,H)=7.8 Hz, 2H, CH of 3’ and 5’), 8.53 (dd, ^3J(H,H)=7.9 Hz, ^4J(H,H)=0.8 Hz, 2H, CH of 3 and 3”); ^{13}C NMR (75 MHz, $CDCl_3$, 25°C, TMS): δ=34.3 (2 C, $\mathbf{CH_2}$-Br), 120.5 (2 C, CH of 3+3” or 5+5” or 3’+5’), 121.6 (2 C, CH of 3+3” or 5+5” or 3’+5’), 123.6 (2 C, CH of 3+3” or 5+5” or 3’+5’), 138.0 (2 C, CH of 4+4”), 138.1 (2 C, CH of 4’), 155.1 (2 C, C of 6+6”), 156.1 (2 C, C of 2+2” or 2’+5’), 156.4 (2 C, C of 2+2” or 2’+5’); HRMS (ESI+): *m/z* (%): 417.9539 $[M+H]^+$ (50), 419.9517 $[M+H]^+$ (100), 421.9496 $[M+H]^+$ (50), ($C_{17}H_{13}N_3Br_2$ requires 417.9549 $[M+H]^+$ (50), 419.9529 $[M+H]^+$ (100), 421.9508 $[M+H]^+$ (50)).

Synthesis of Z-Dab(Boc)-OH: Z-Dab(Boc)-OH·DCHA (100 mg, 187 µmol) was suspended in EtOAc (1.5 mL) and, while stirring, 1 M H_3PO_4 (1.0 mL, 0.94 mmol, 5 equiv.) was added dropwise at r.t. The phases were separated and washed thrice with 1 M H_3PO_4 (1 mL), once with water, and twice with brine. The organic solution was dried with Na_2SO_4 and concentrated in vacuum to obtain **Z-Dab(Boc)-OH** (62.1 mg, 176 µmol, 94%) ^1H NMR (300 MHz, $CDCl_3$, 25°C, TMS): δ=1.46 (s, 9H, C($\mathbf{CH_3}$)$_3$), 1.94-2.44 (m, 2H, β-CH_2), 3.07-3.51 (m, 2H, γ-CH_2), 4.41 (t, ^3J(H,H)=6.36 Hz, 1H, α-CH), 5.11 (s, 2H, $\mathbf{CH_2}$-C_6H_5), 5.77 (d, ^3J(H,H)=6.81 Hz, 1H, NH), 6.77 (brs, 1H, NH), 7.31-7.38 (m, 5H, aromatic CH of C_6H_5); HRMS (ESI+): *m/z* (%): 353.1710 $[M+H]^+$ (100), 370.1973 $[M+NH_4]^+$ (20), 375.1526 $[M+Na]^+$ (10), 391.1266 $[M+K]^+$ (2), 705.3339 $[2M+H]^+$ (40), 727.3156 $[2M+Na]^+$ (20), ($C_{17}H_{24}N_2O_6$ requires 353.1707 $[M+H]^+$, 370.1973 $[M+NH_4]^+$, 375.1527 $[M+Na]^+$, 391.1266 $[M+K]^+$, 705.3342 $[2M+H]^+$, 727.3161 $[2M+Na]^+$).

Synthesis of *tert*-butyl 2-(benzyl(2-hydroxyethyl)amino)acetate (8): *N-B*enzylethanolamine (2.50 mL, 16.5 mmol) was placed in a round bottom flask, DIPEA (2.13 mL, 16.5 mmol) and dry DMF (20 mL) were added. The solution was stirred at 0°C and *tert*-butyl bromoacetate (2.44 mL, 16.5 mmol) was added dropwise. The mixture was allowed to warm up to room temperature and was stirred over night at r.t. The solvent was removed under reduced pressure and the residue was dissolved in DCM (250 mL). The organic solution was washed four times with water (100 mL), dried with Na_2SO_4 and concentrated in vacuum to obtain **8** (4.40 g, 16.5 mmol, 100%). ^1H NMR (300 MHz, $CDCl_3$, 25°C, TMS): δ=1.45 (s, 9H, C($\mathbf{CH_3}$)$_3$), 2.85 (t, ^3J(H,H)=5.2 Hz, 2H, N-$\mathbf{CH_2}$-CH_2-OH), 3.22 (s, 2H, N-$\mathbf{CH_2}$-$COOC_4H_9$), 3.26

(brs, 1H, OH), 3.58 (t, ^3J(H,H)=5.1 Hz, 2H, N-CH_2-$\mathbf{CH_2}$-OH), 3.81 (s, 2H, $\mathbf{CH_2}$-C_6H_5), 7.24-7.33 (m, 5H, $\mathbf{C_6H_5}$); ^{13}C NMR (75 MHz, $CDCl_3$, 25°C, TMS): δ=28.1 (3 C, C($\mathbf{CH_3}$)$_3$), 55.5 (1 C, N-$\mathbf{CH_2}$-$\mathbf{CH_2}$-OH), 56.7 (1 C, N-$\mathbf{CH_2}$-$\mathbf{CH_2}$-OH), 58.7 (1 C, $\mathbf{CH_2}$-$COOC_4H_9$), 59.1 (1 C, $\mathbf{CH_2}$-C_6H_5), 81.3 (1 C, **C**(CH_3)$_3$), 127.3 (1 C, para-CH of C_6H_5), 128.4 (2 C, meta-CH of C_6H_5), 128.9 (2 C, ortho-CH of C_6H_5), 138.6 (1 C, ipso-C of C_6H_5), 171.2 (1 C, **C**OOC_4H_9); HRMS (ESI+): *m/z* (%): 266.1756 $[M+H]^+$ (100), 288.1573 $[M+Na]^+$ (10), ($C_{15}H_{23}N_1O_3$ requires 266.1751 $[M+H]^+$, 288.1570 $[M+Na]^+$).

Synthesis of *tert*-butyl 2-(benzyl(2-bromoethyl)amino)acetate (9): 8 (750 mg, 2.83 mmol) and triphenylphosphine (871 mg, 3.32 mmol, 1.18 equiv.) were combined and dried three times by addition and evaporation of dry toluene. To remove residual toluene, dry DCM was added and evaporated three times. The mixture was immediately dissolved in dry DCM (9.5 mL) under argon atmosphere. *N*-Bromosuccinimide (587 mg, 3.30 mmol, 1.17 equiv., dried in vacuum at 10 – 100 Pa) was added in portions over a period of 1 h at 0°C. The mixture was stirred for another 0.5 h at 0°C and subsequently the solution was allowed to warm up to r.t. Reaction controls were performed by TLC (silica gel; $CHCl_3$:MeOH:acetic acid = 9:1:0.1, R_f=0.87). After 4 h of stirring at r.t. the solvent was removed under reduced pressure. The crude product was purified by silica gel chromatography (gradient elution starting with $CHCl_3$:*n*-heptane = 2:3 and ending with $CHCl_3$:MeOH = 9:1) to obtain **9** (418 mg, 1.27 mmol, 45%). ^{1}H NMR (300 MHz, $CDCl_3$, 25°C, TMS): δ=11.47 (s, 9H, C($\mathbf{CH_3}$)$_3$), 3.13 (t, ^3J(H,H)=7.2 Hz, 2H, N-$\mathbf{CH_2}$-CH_2-Br), 3.30 (s, 2H, N-$\mathbf{CH_2}$-$COOC_4H_9$), 3.37 (t, ^3J(H,H)=7.3 Hz, 2H, N-CH_2-$\mathbf{CH_2}$-Br), 3.87 (s, 2H, N-$\mathbf{CH_2}$-C_6H_5), 7.22-7.37 (m, 5H, CH_2-$\mathbf{C_6H_5}$); ^{13}C NMR (75 MHz, $CDCl_3$, 25°C, TMS): δ=28.3 (3 C, C($\mathbf{CH_3}$)$_3$), 30.7 (1 C, N-CH_2-$\mathbf{CH_2}$-Br), 55.4 (1 C, N-$\mathbf{CH_2}$-$COOC_4H_9$), 56.0 (1 C, N-$\mathbf{CH_2}$-CH_2-Br), 58.2 (1 C, N-$\mathbf{CH_2}$-C_6H_5), 81.3 (1 C, **C**(CH_3)$_3$), 127.4 (1 C, para-CH of C_6H_5), 128.5 (2 C, meta-CH of C_6H_5), 128.9 (2 C, ortho-CH of C_6H_5), 138.9 (1 C, ipso-C of C_6H_5), 170.7 (1 C, **C**OOC_4H_9); HRMS (ESI+): *m/z* (%): 248.1645 $[M-Br^-]^+$ (100), 328.0908 $[M+H]^+$ (80), 330.0886 $[M+H]^+$ (80), ($C_{15}H_{22}N_1O_2Br$ requires 248.1645 $[M-Br^-]^+$, 328.0907 $[M+H]^+$, 330.0886 $[M+H]^+$).

References

[1] C. K. Riener, G. Kada, H. J. Gruber, *Anal. Bioanal. Chem.* **2002**, *373*, 266.
[2] A. V. Elgersma, R. L. Zsom, W. Norde, J. Lyklema, *J. Colloid Interface Sci.* **1990**, *138*, 145.
[3] A. M. Brouwer, *Pure Appl. Chem.* **2011**, *83*.
[4] R. F. Kubin, A. N. Fletcher, *J. Lumin.* **1982**, *27*, 455.

NMR-, mass- and UV/Vis-spectra of the synthesized compounds:

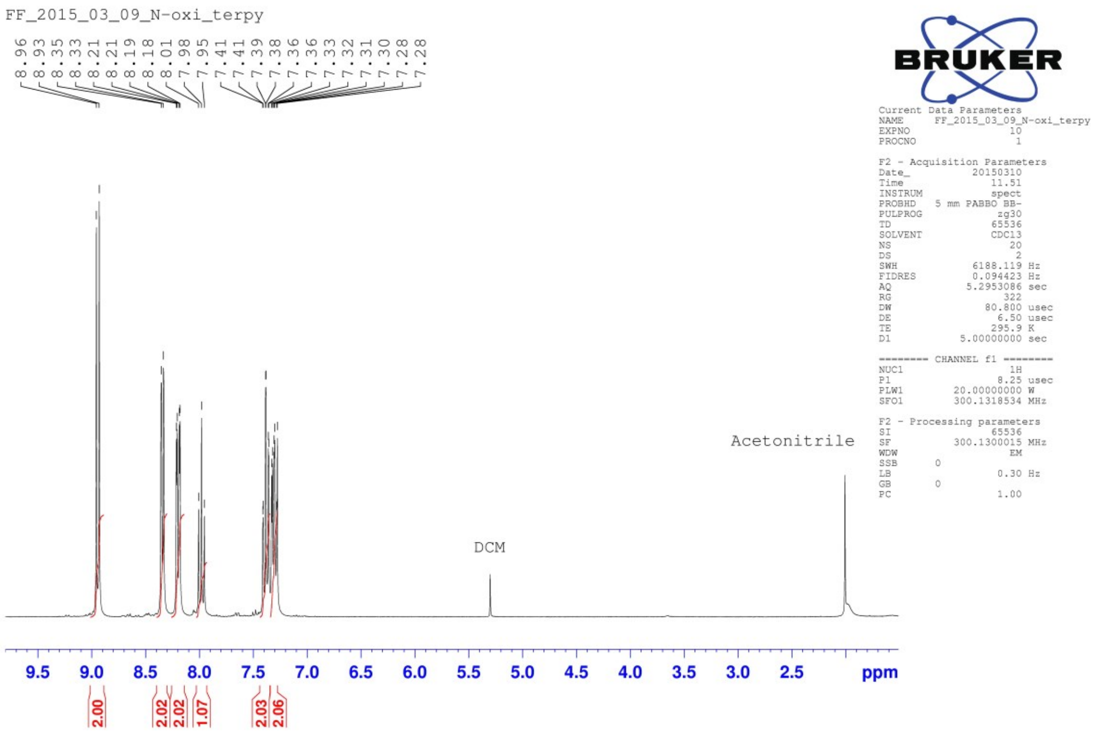

Figure S7: ^{1}H-NMR of compound **1**.

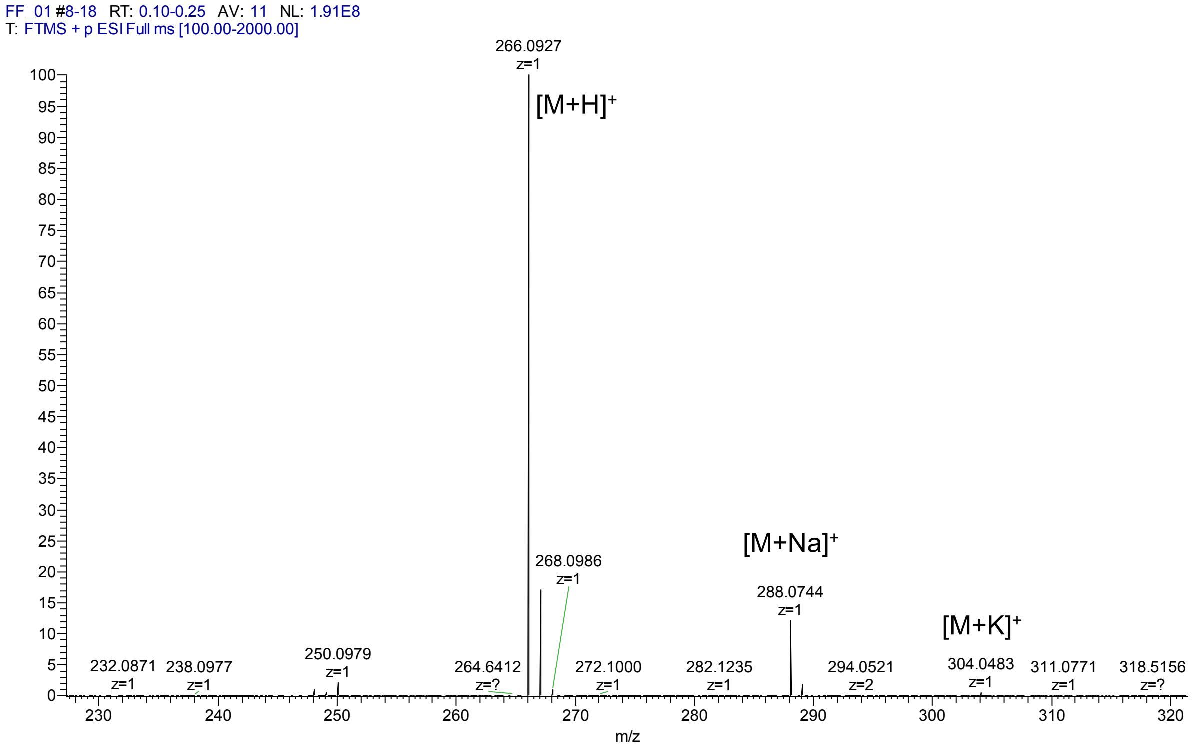

Figure S8: High resolution mass-spectrum of compound **1**.

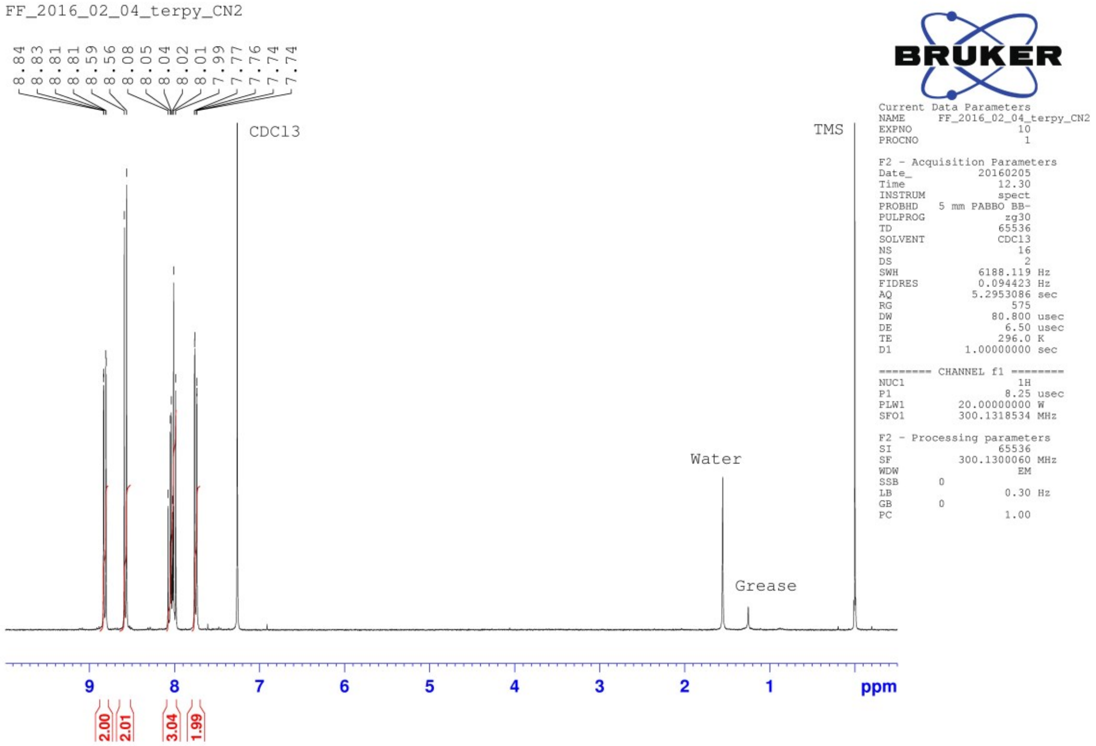

Figure S9: ^{1}H-NMR of compound **2**.

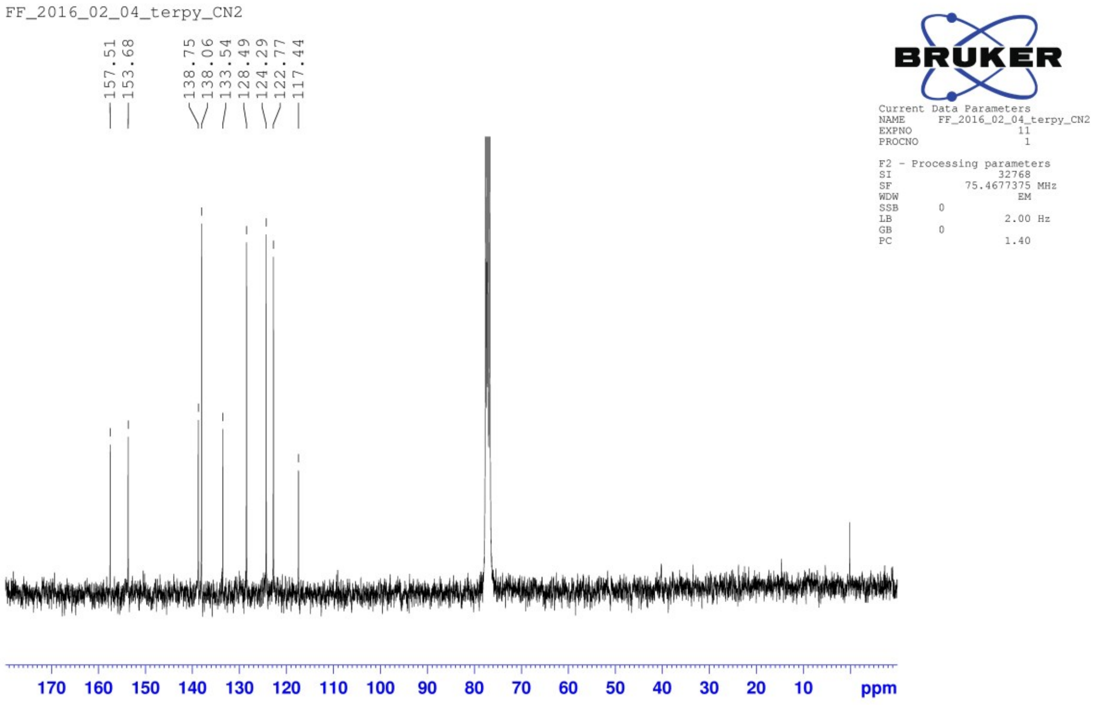

Figure S10: ^{13}C-NMR of compound **2**.

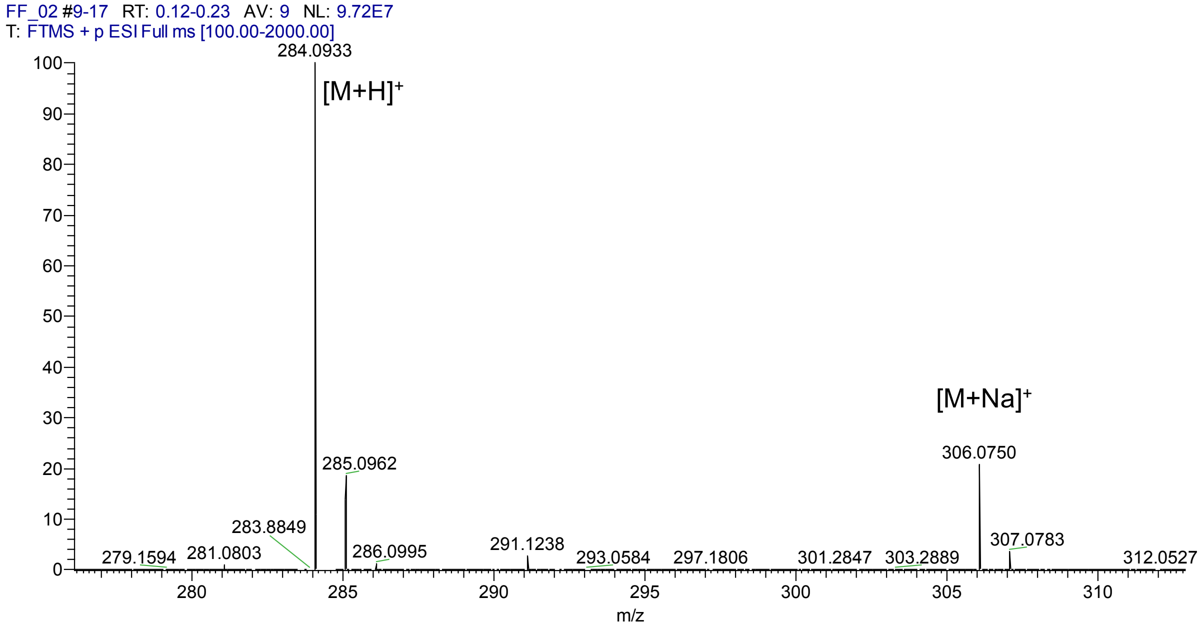

Figure S11: High resolution mass-spectrum of compound **2**.

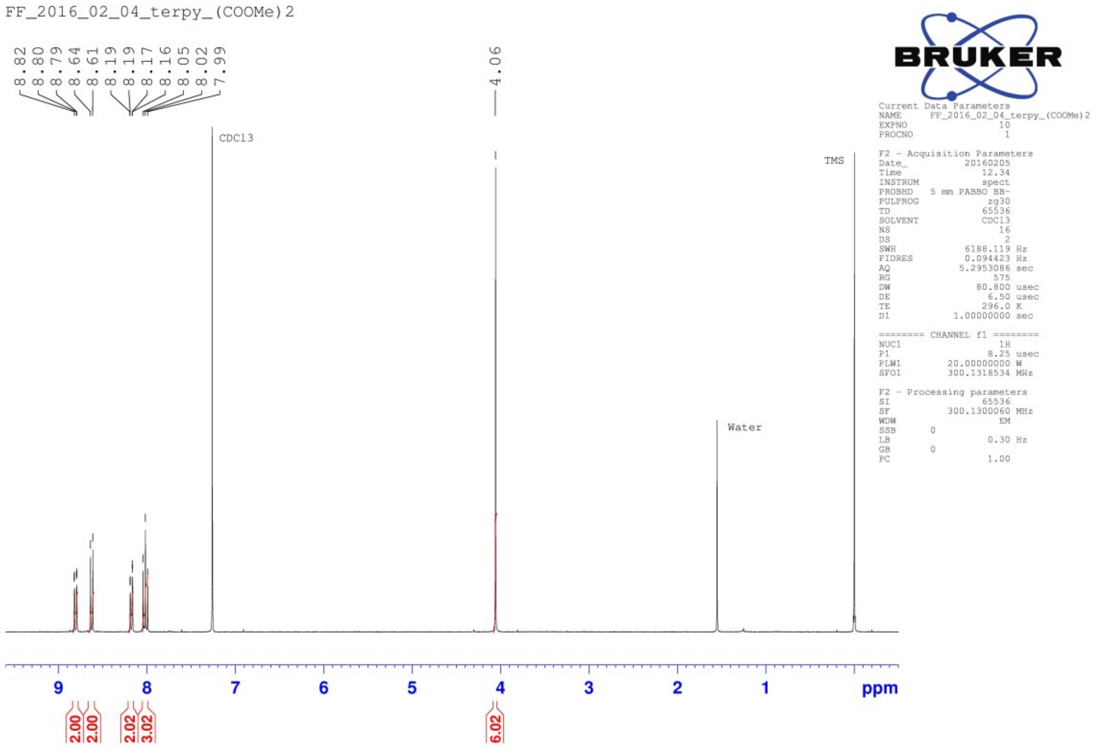

Figure S12: ^{1}H-NMR of compound **3**.

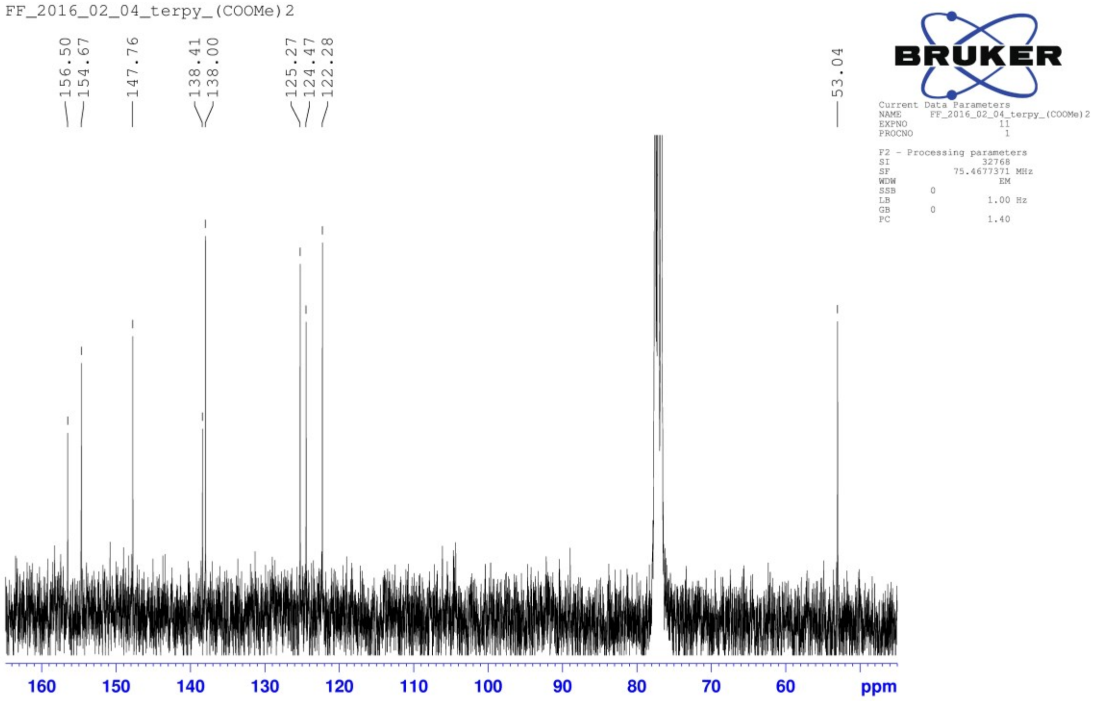

Figure S13: ^{13}C-NMR of compound **3**.

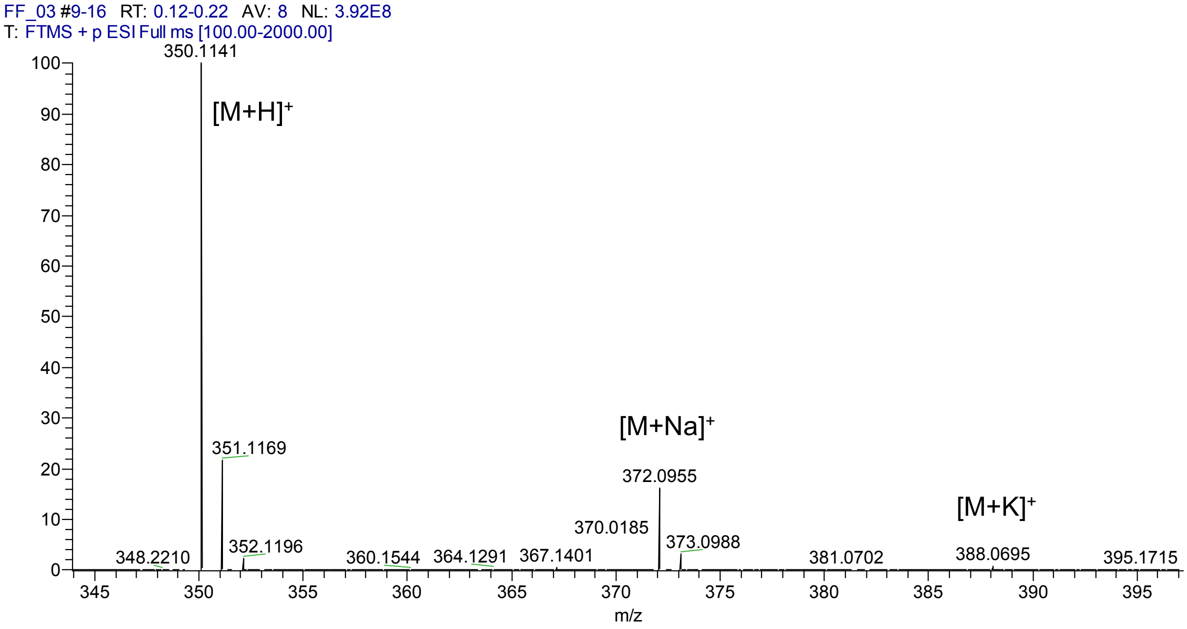

Figure S14: High resolution mass-spectrum of compound **3**.

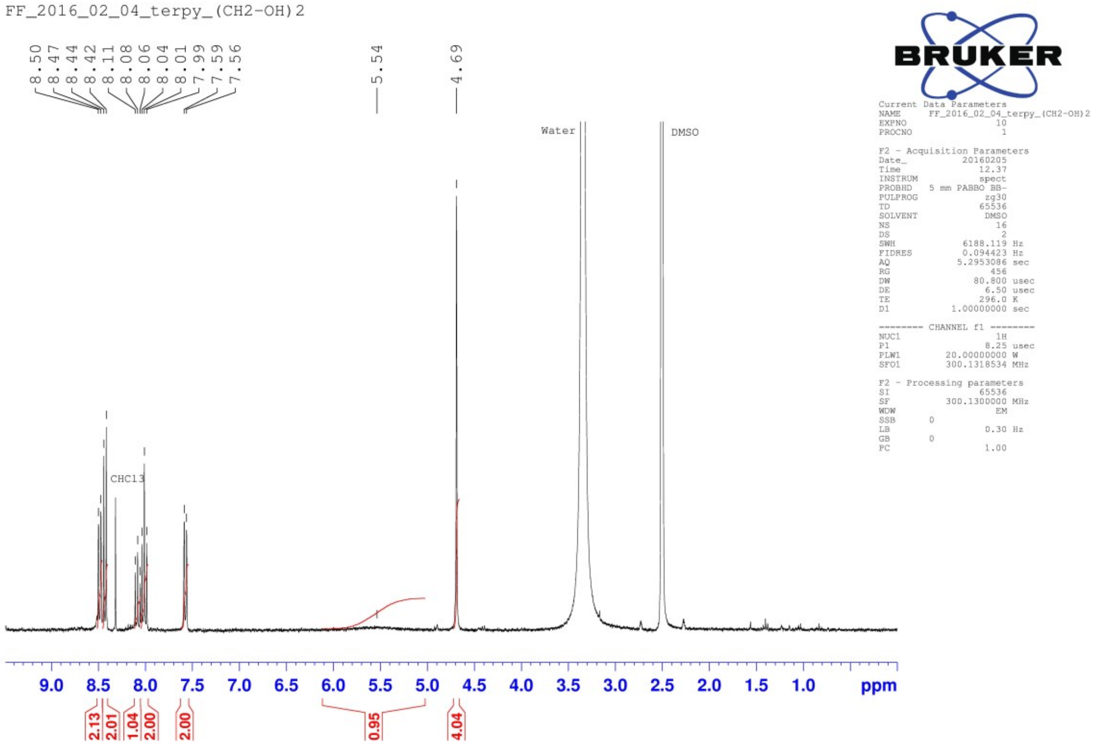

Figure S15: ^{1}H-NMR of compound **4**.

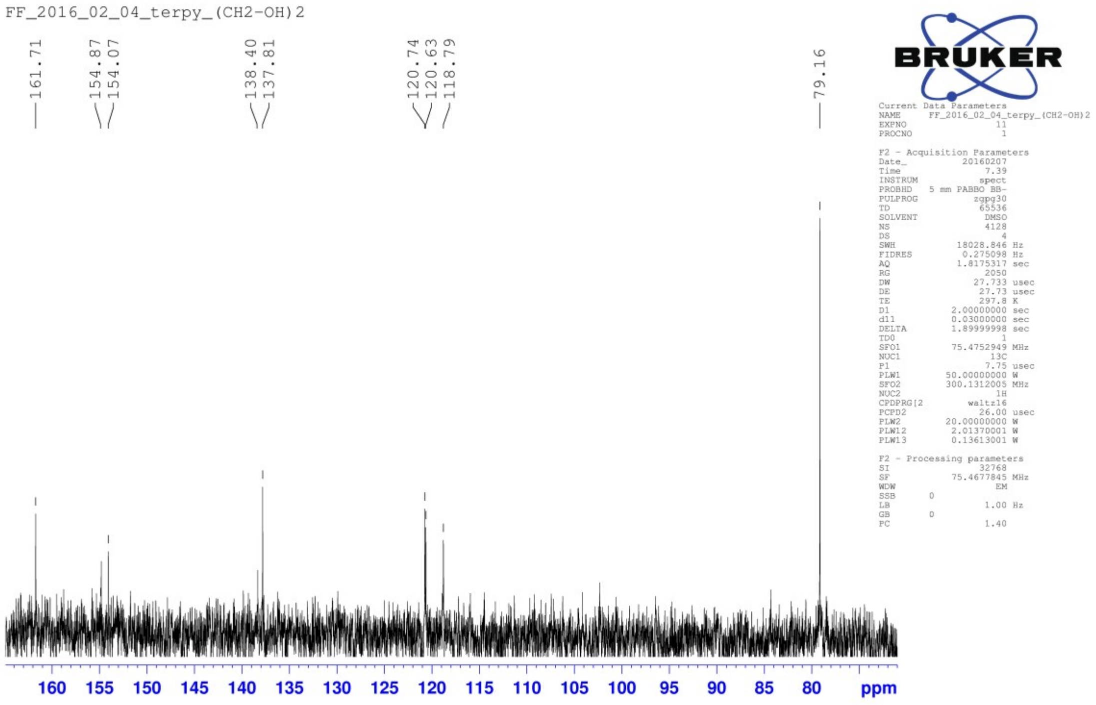

Figure S16: ^{13}C-NMR of compound **4**.

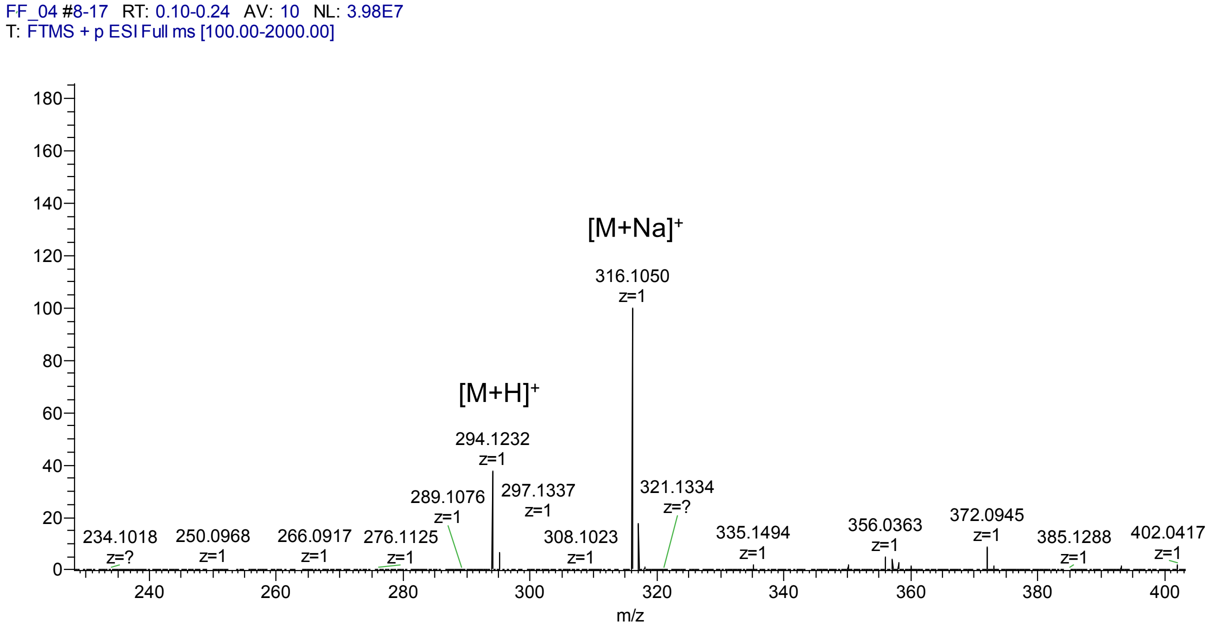

Figure S17: High resolution mass-spectrum of compound **4**.

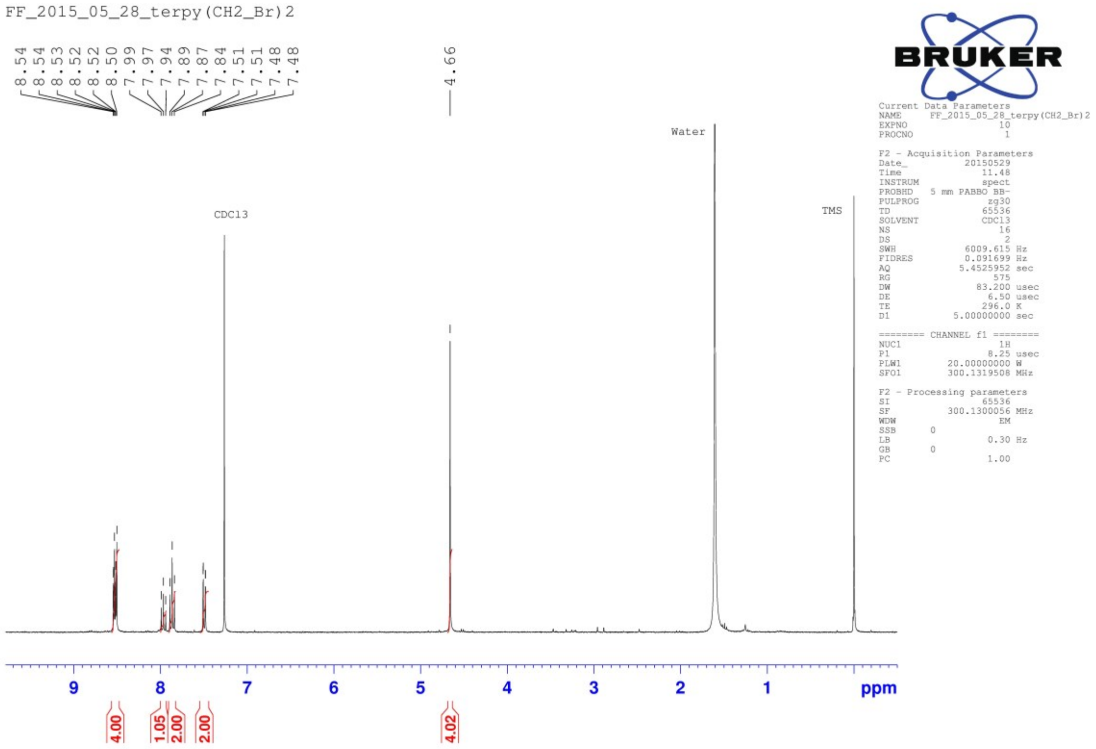

Figure S18: ^{1}H-NMR of compound **5**.

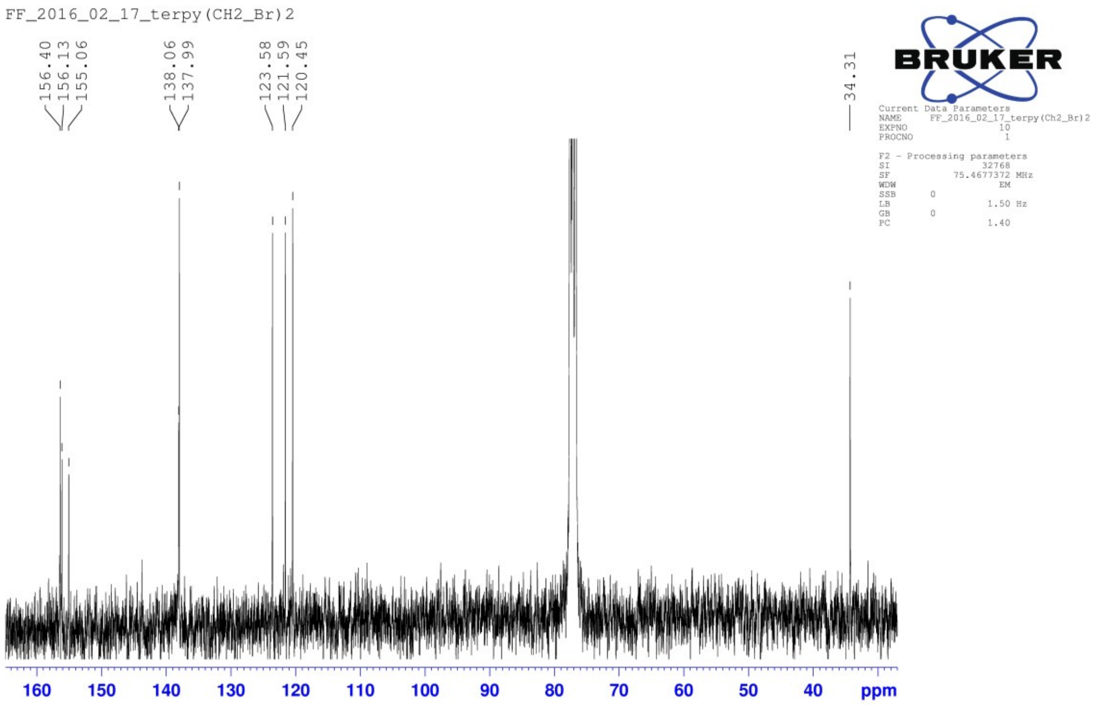

Figure S19: ^{13}C-NMR of compound **5**.

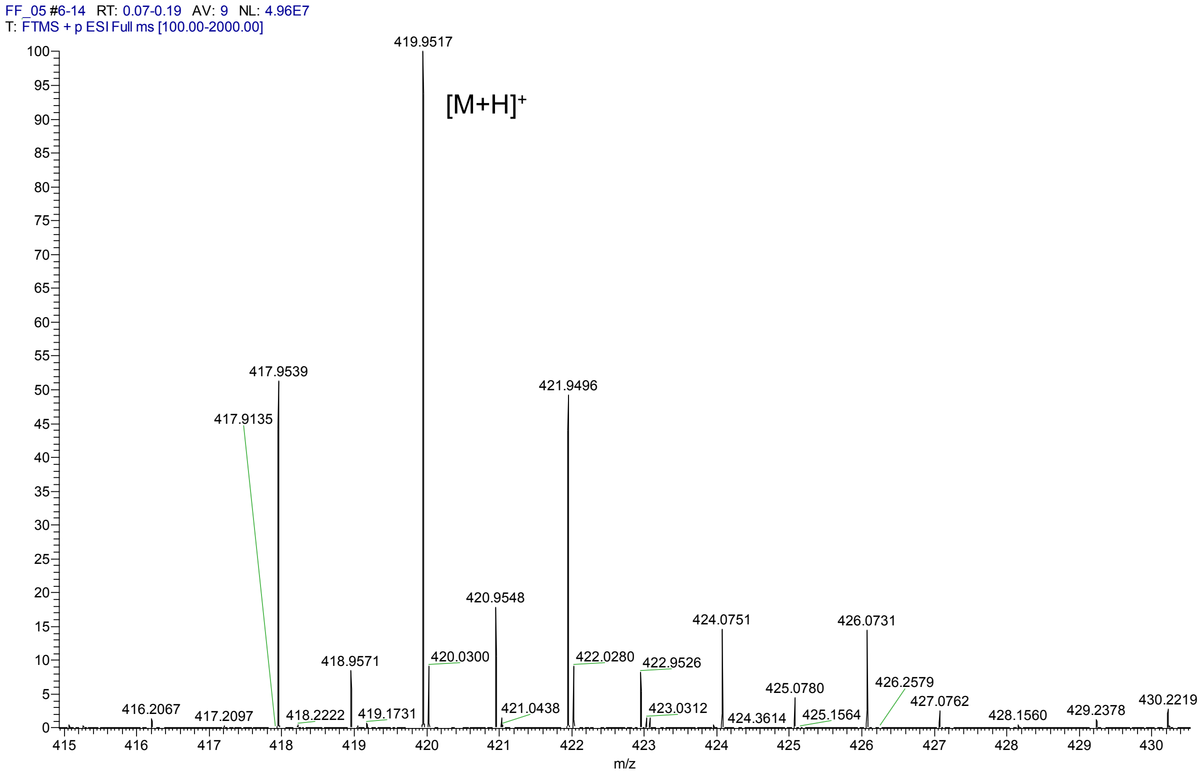

Figure S20: High resolution mass-spectrum of compound **5**.

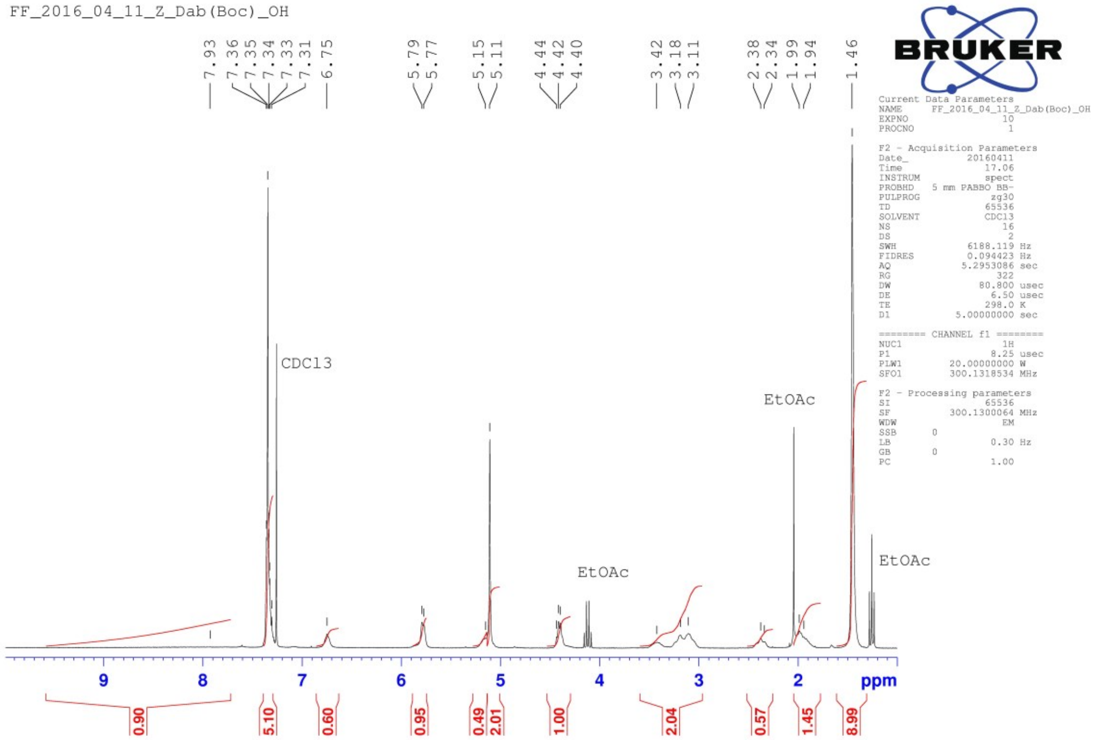

Figure S21: ^{1}H-NMR of **Z-Dab(Boc)-OH**.

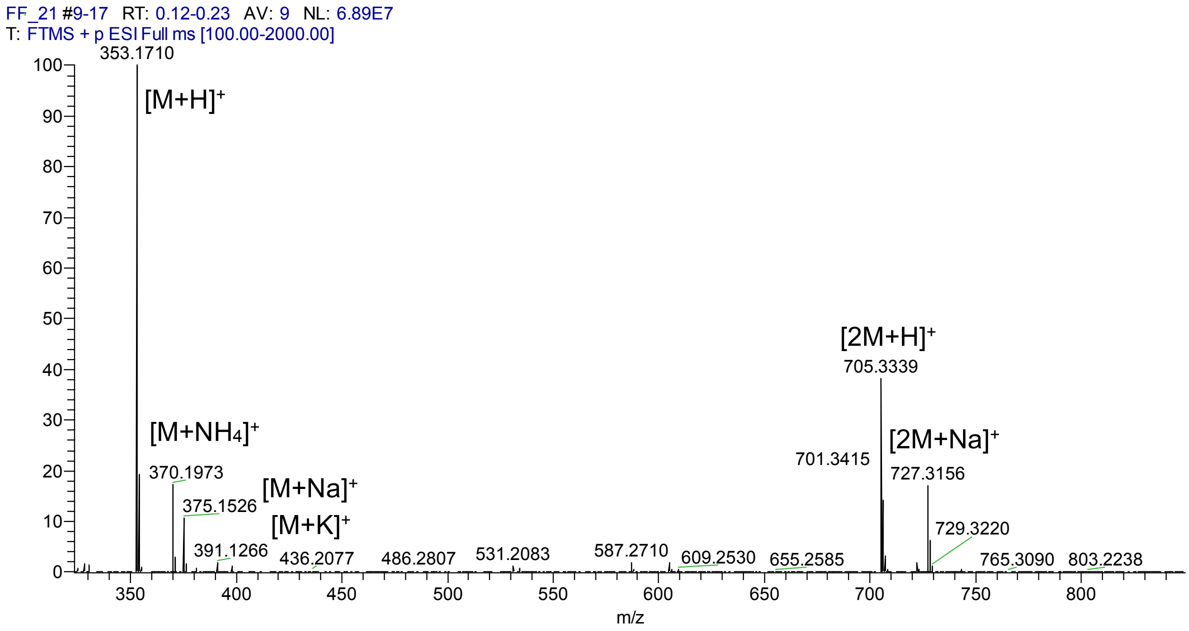

Figure S22: High resolution mass-spectrum of compound **Z-Dab(Boc)-OH**.

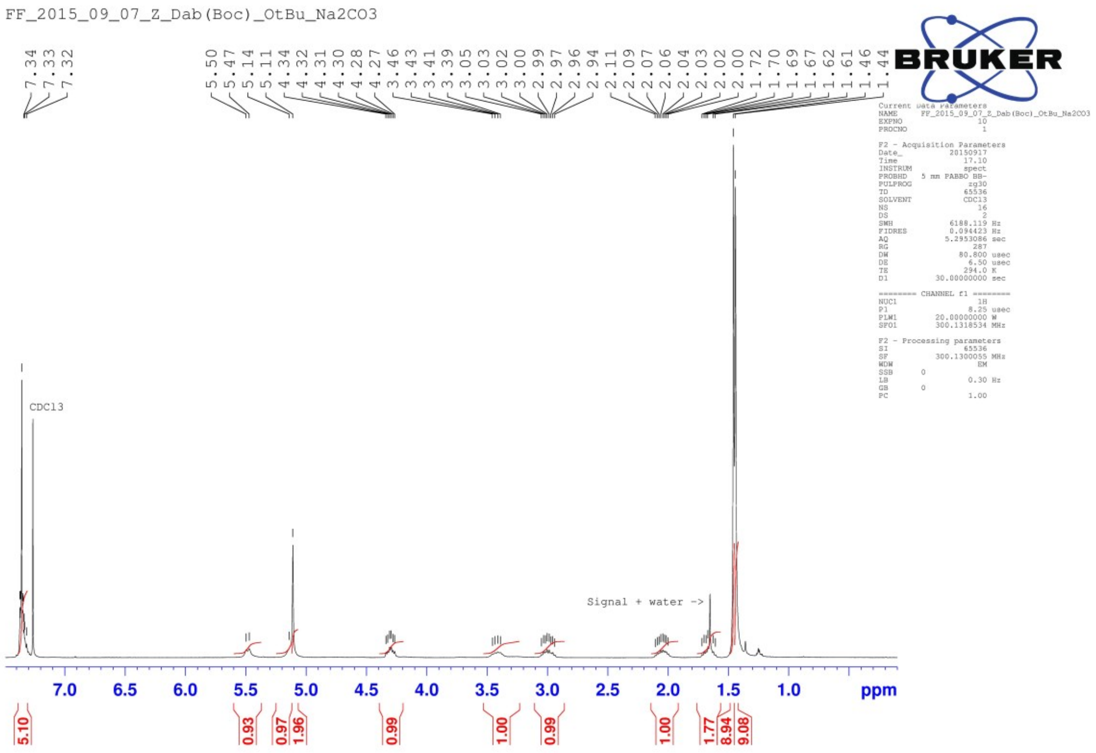

Figure S23: ^{1}H-NMR of compound **6**.

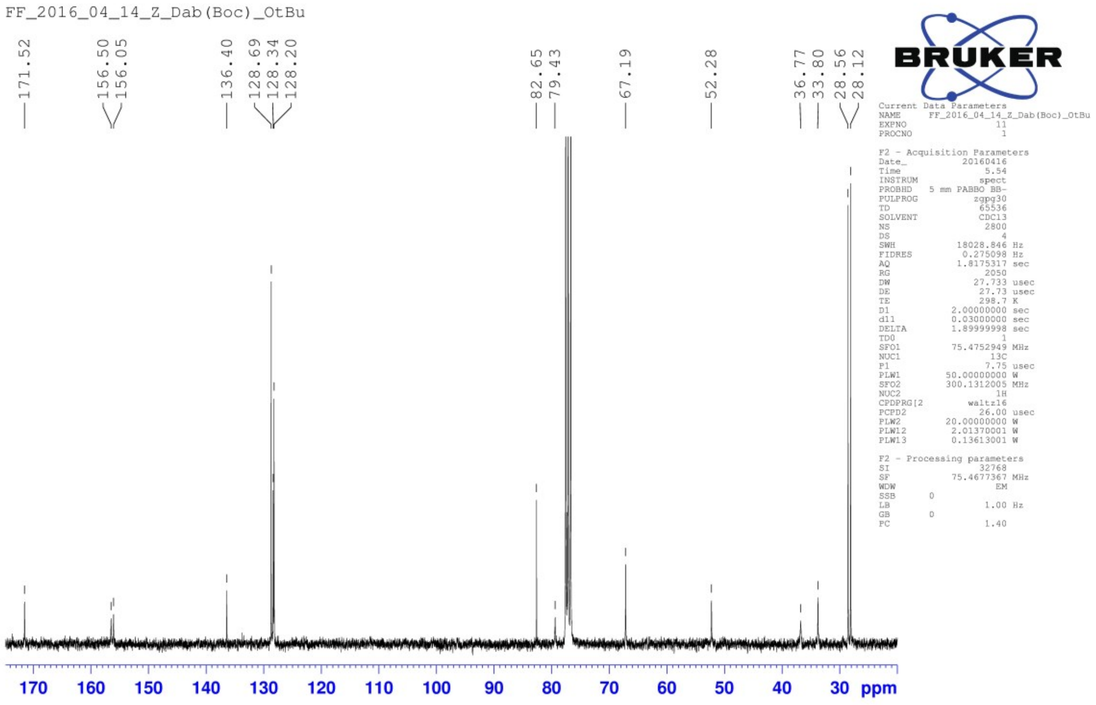

Figure S24: ^{13}C-NMR of compound **6**.

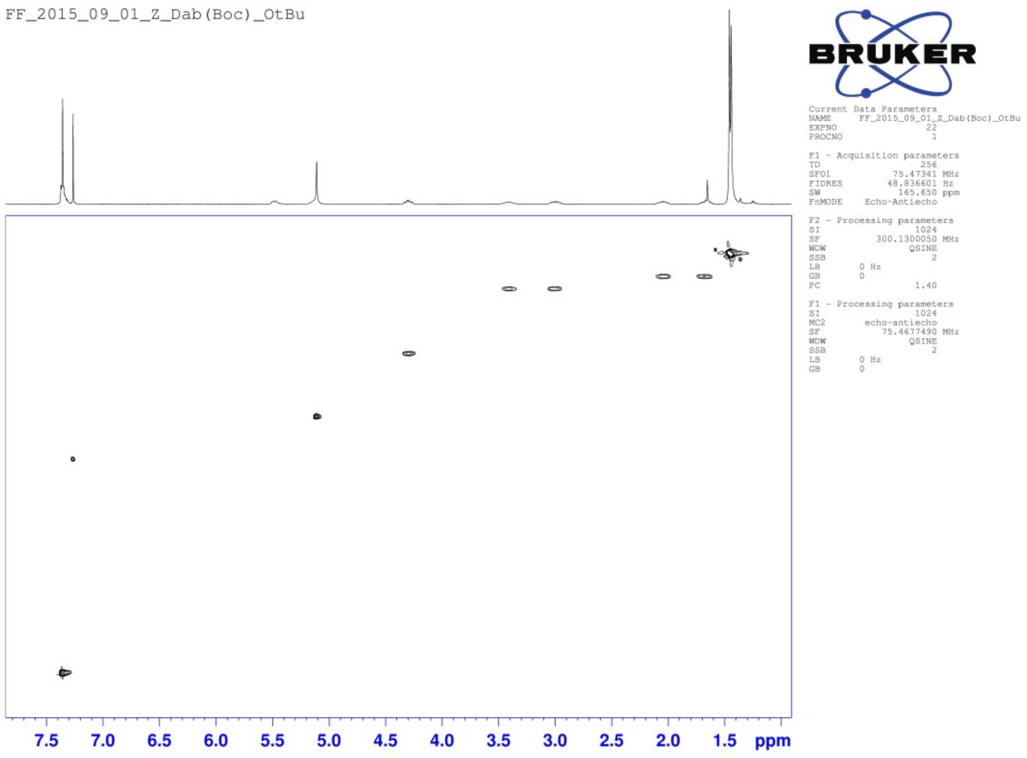

Figure S25: HSQC-NMR of compound **6**.

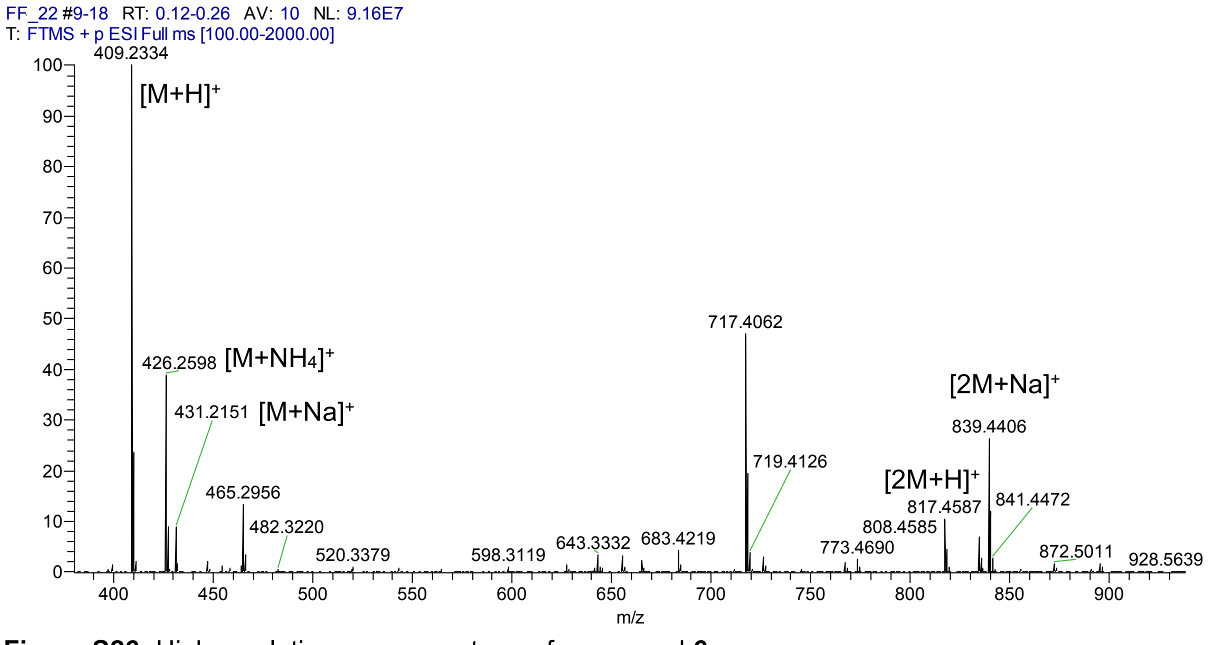

Figure S26: High resolution mass-spectrum of compound **6**.

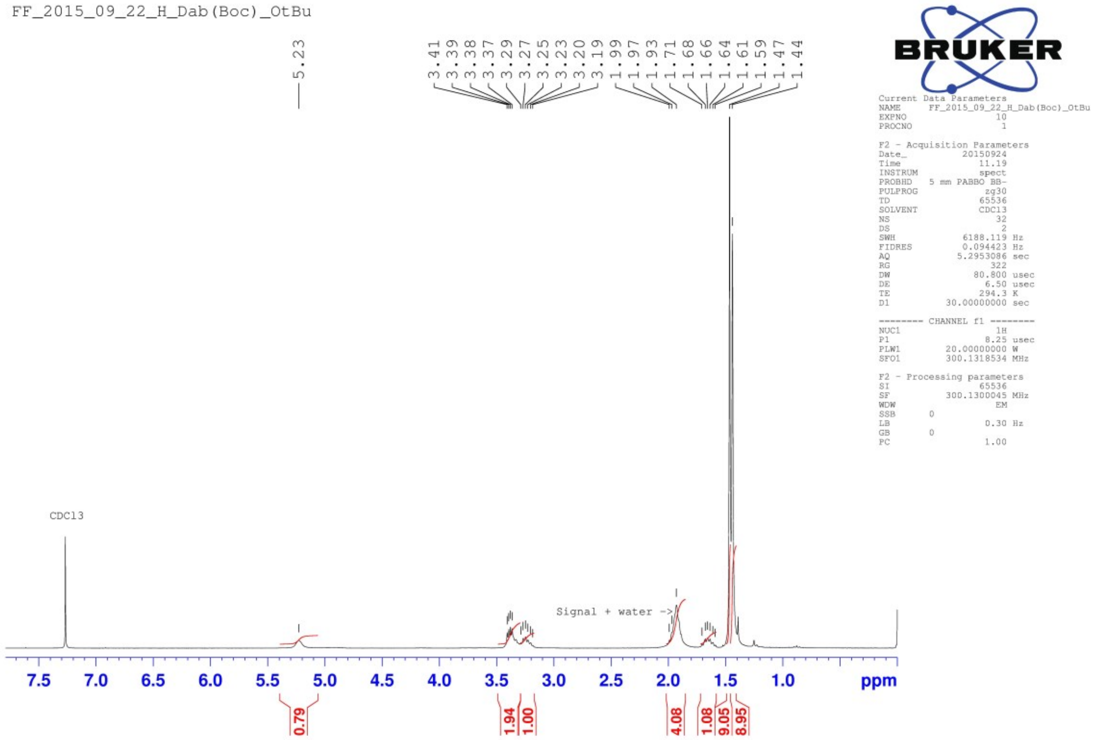

Figure S27: ^{1}H-NMR of of compound **7**.

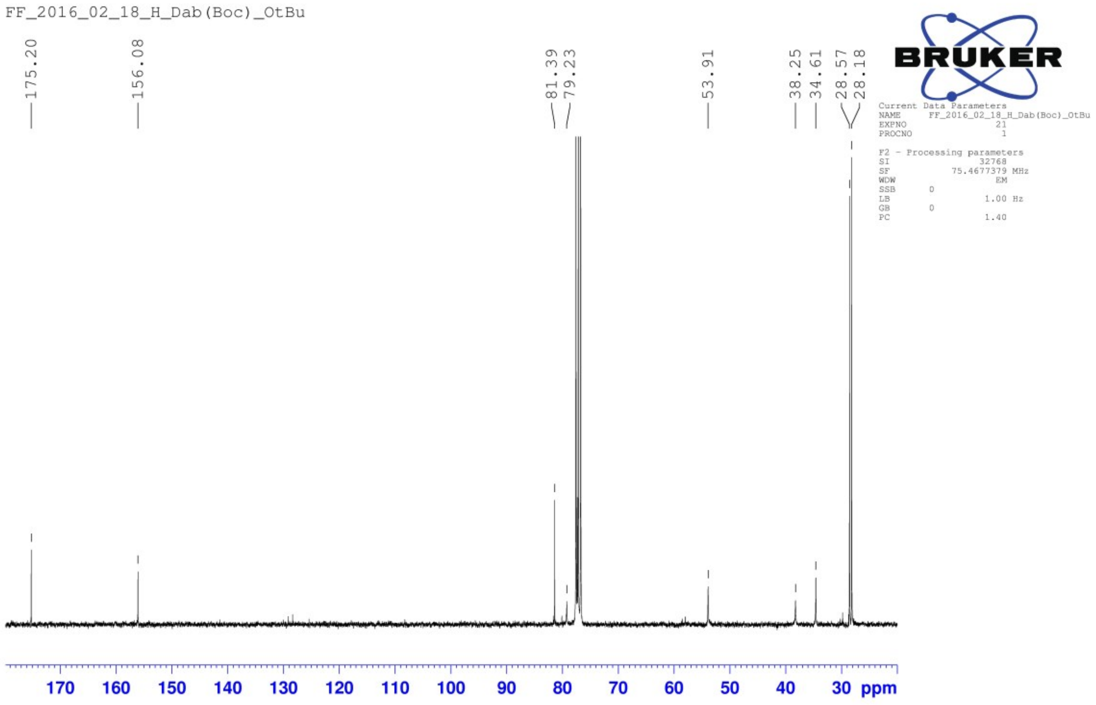

Figure S28: ^{13}C-NMR of compound **7**.

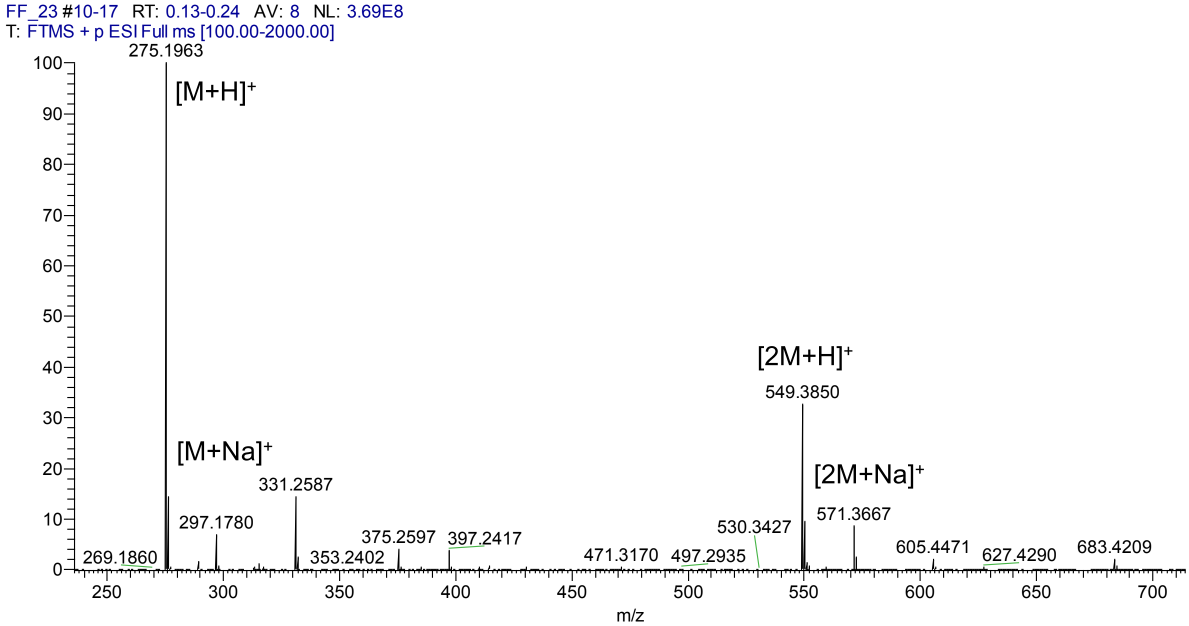

Figure S29: High resolution mass-spectrum of compound **7**.

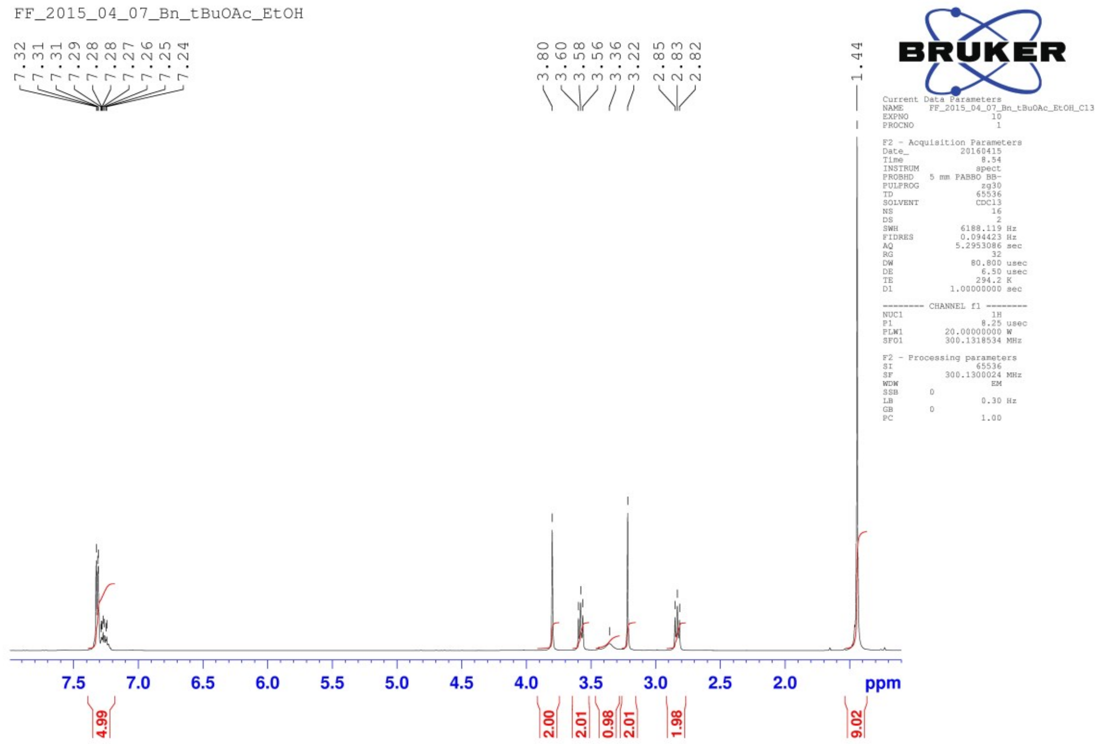

Figure S30: ^{1}H-NMR of compound **8**.

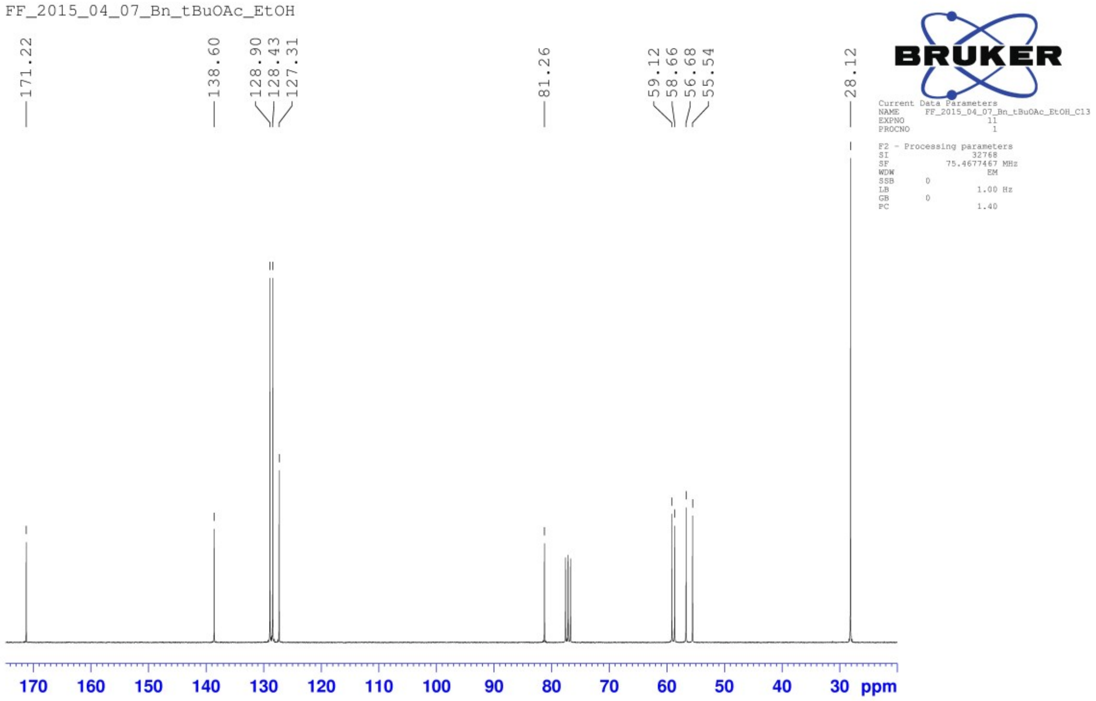

Figure S31: ^{13}C-NMR of compound **8**.

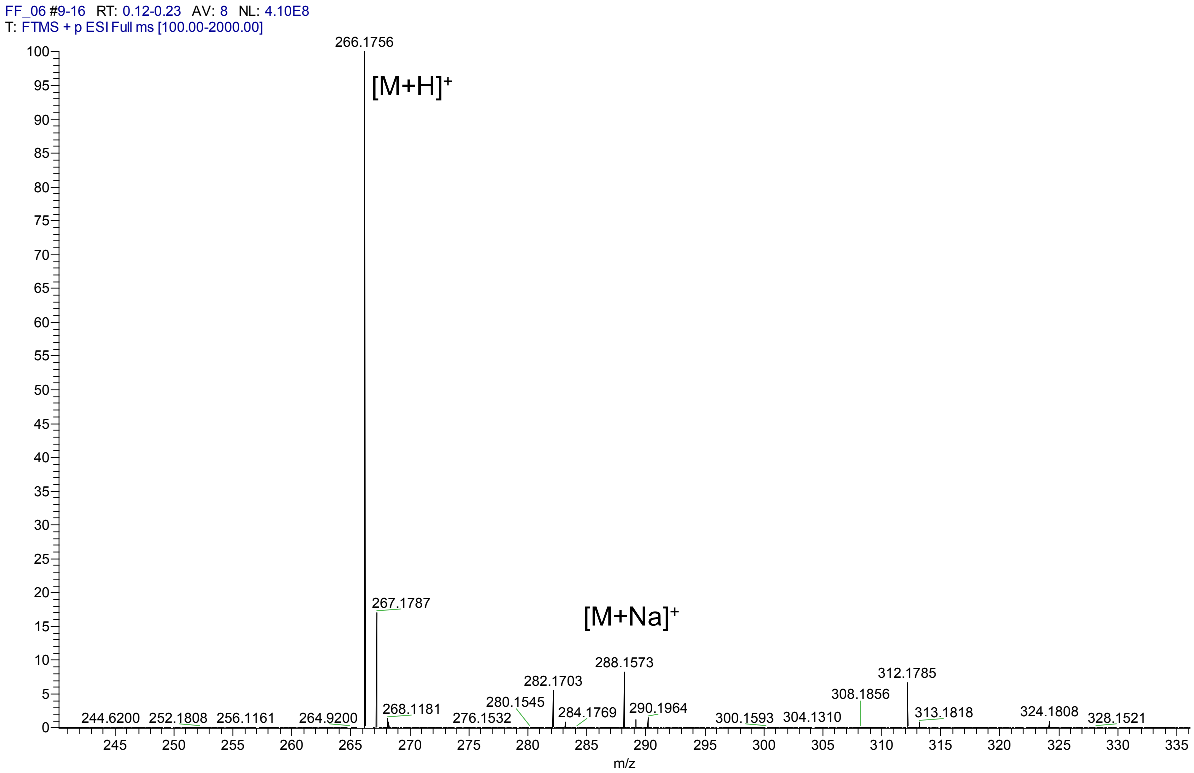

Figure S32: High resolution mass-spectrum of compound **8**.

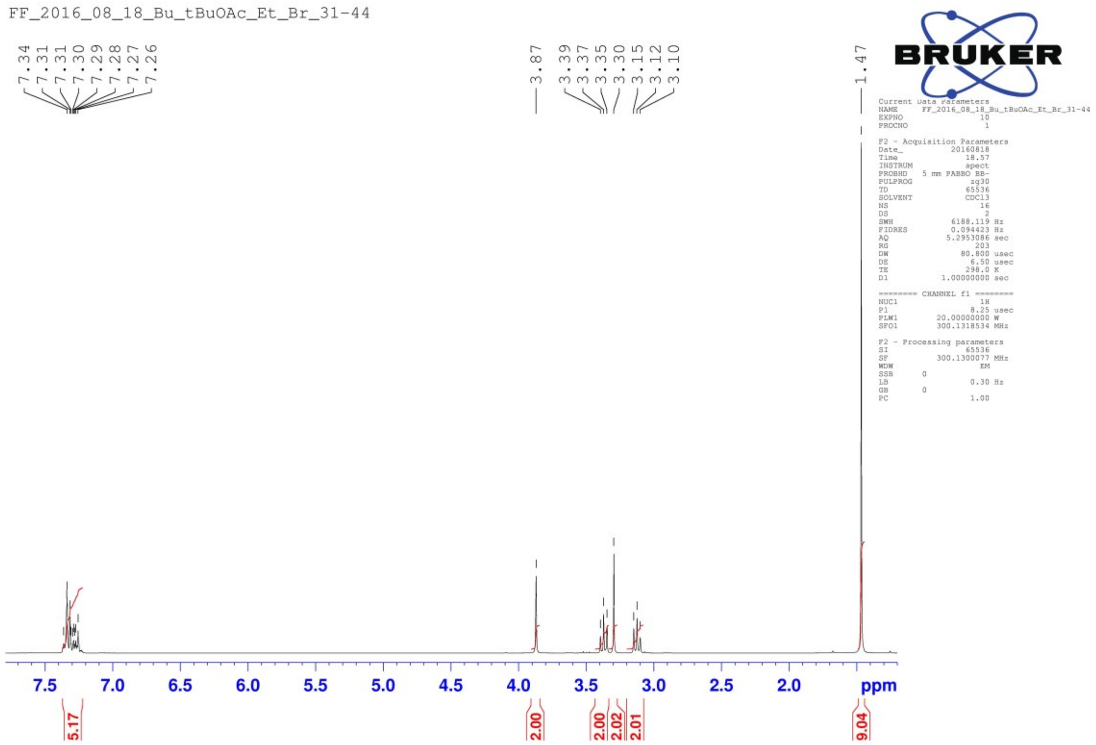

Figure S33: ^{1}H-NMR of compound **9**.

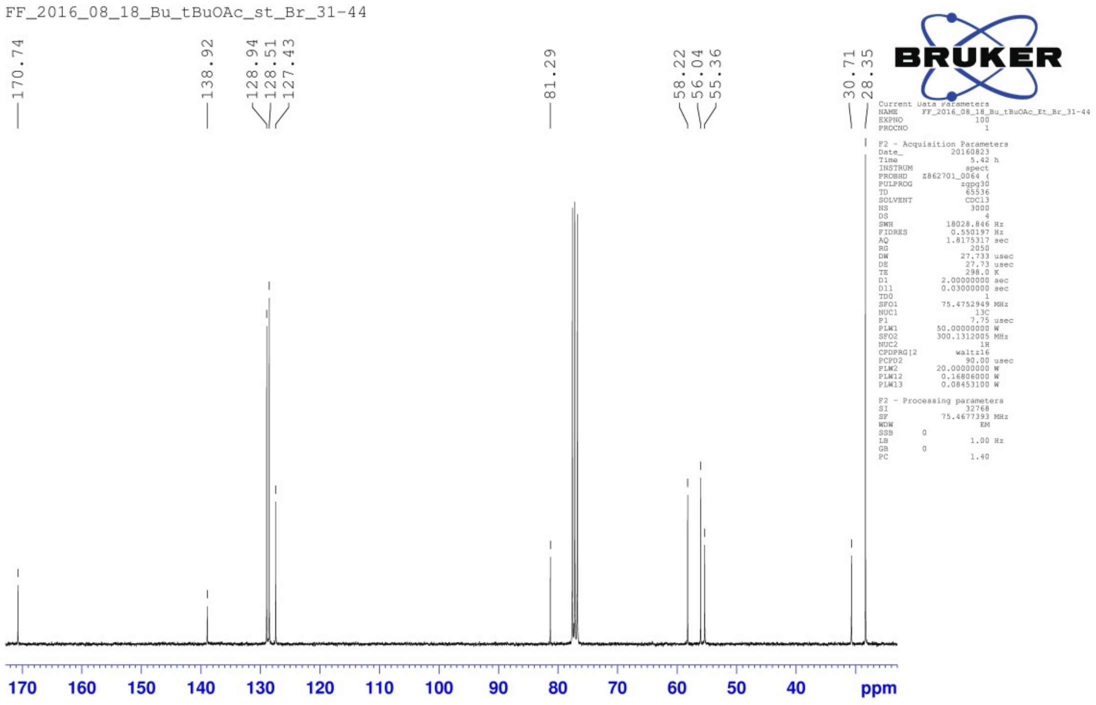

Figure S34: ^{13}C-NMR of compound **9**.

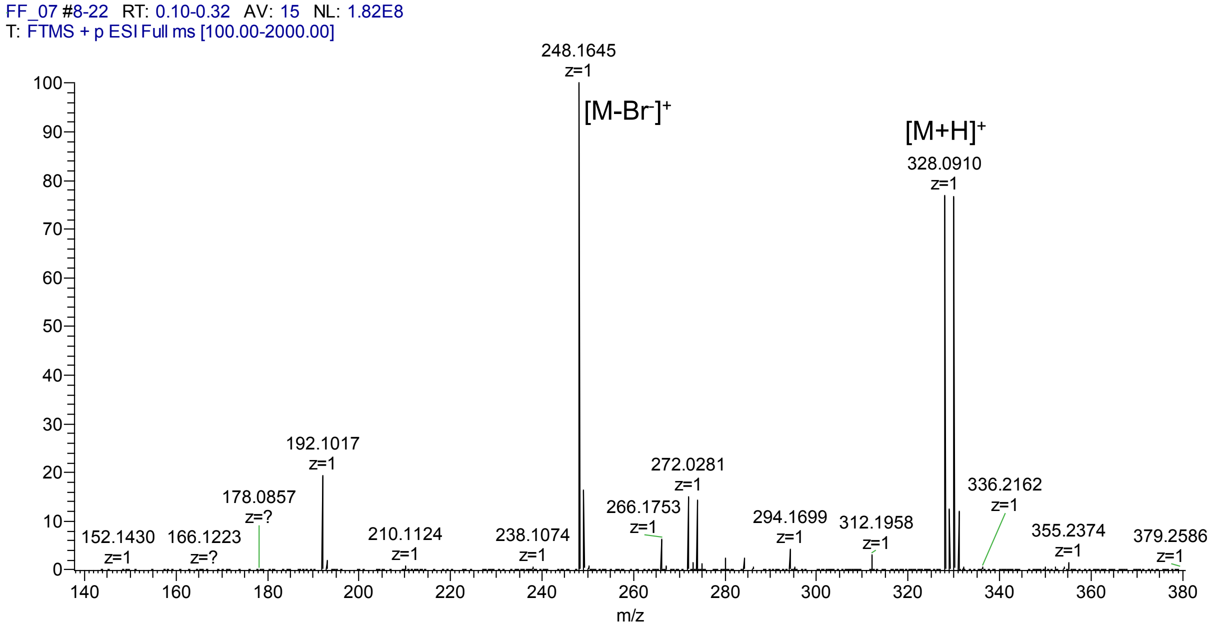

Figure S35: High resolution mass-spectrum of compound **9**.

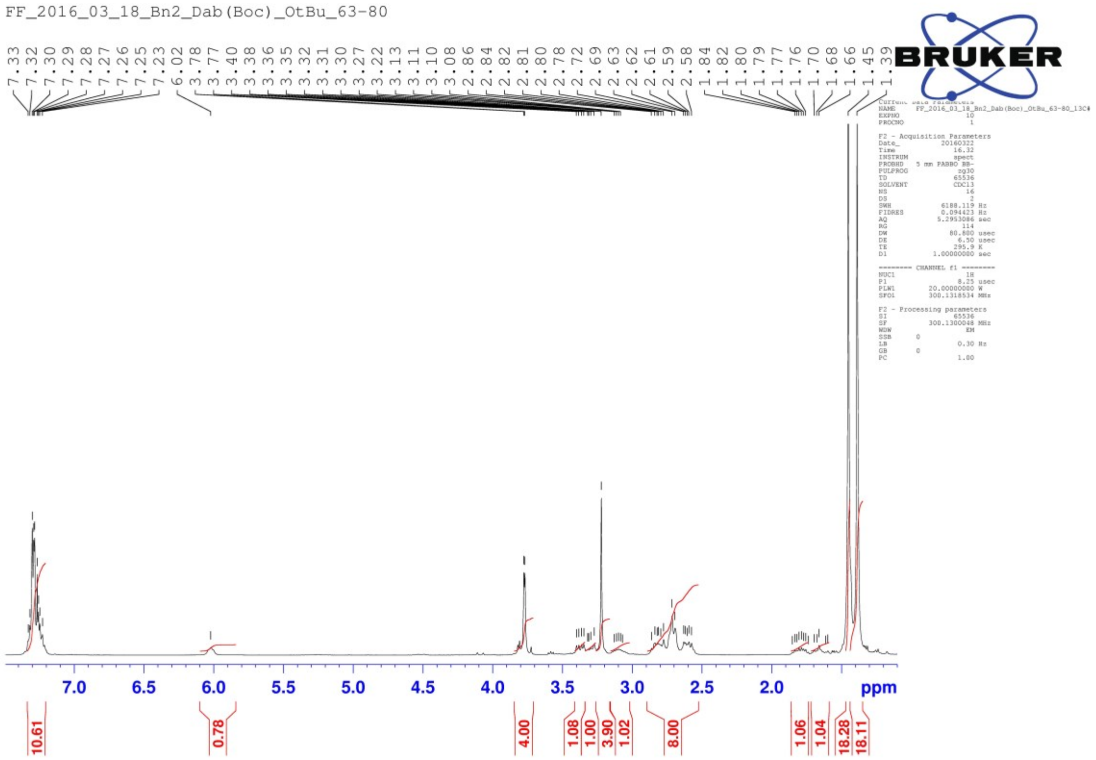

Figure S36: ^{1}H-NMR of compound **10**.

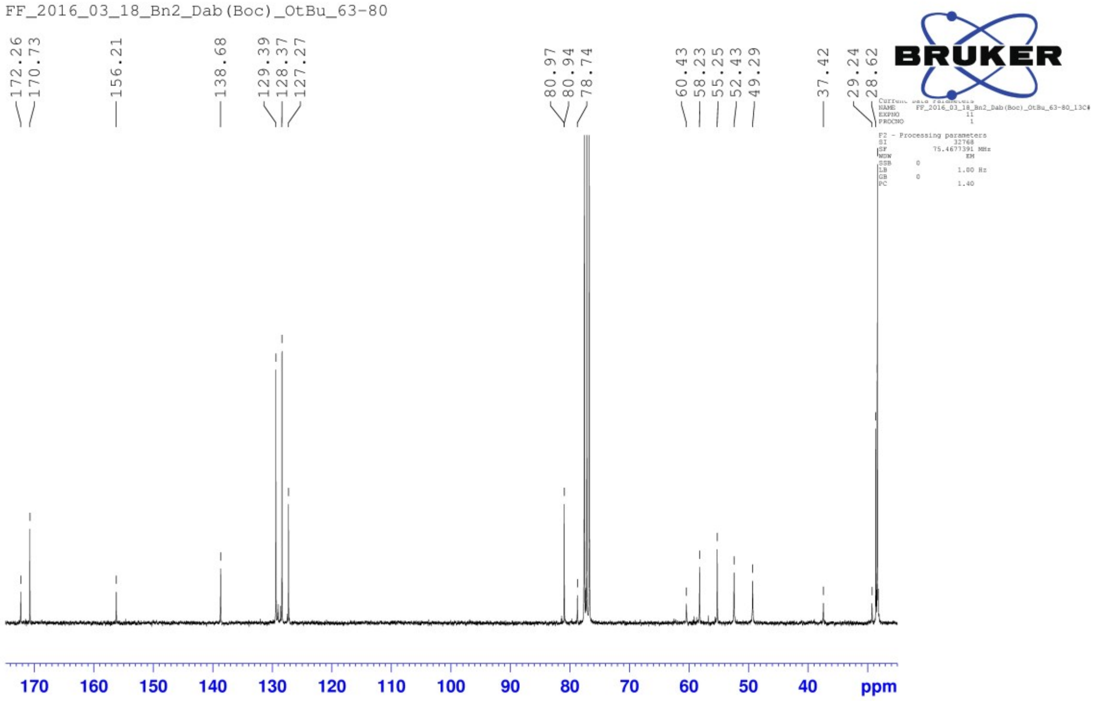

Figure S37: ^{13}C-NMR of compound **10**.

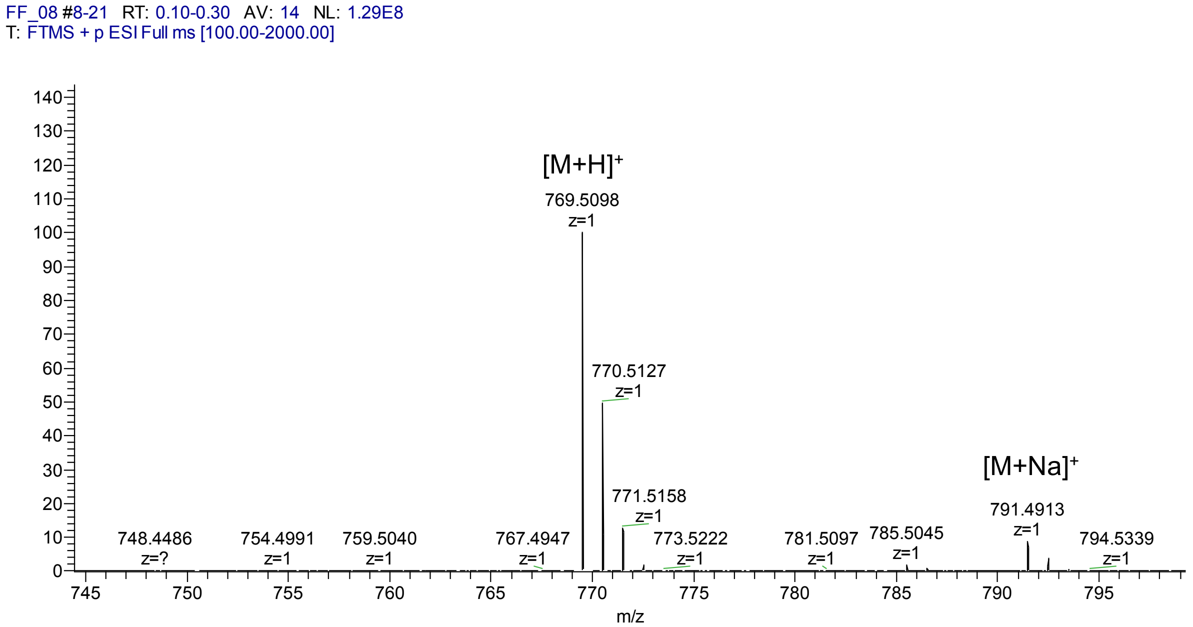

Figure S38: High resolution mass-spectrum of compound **10**.

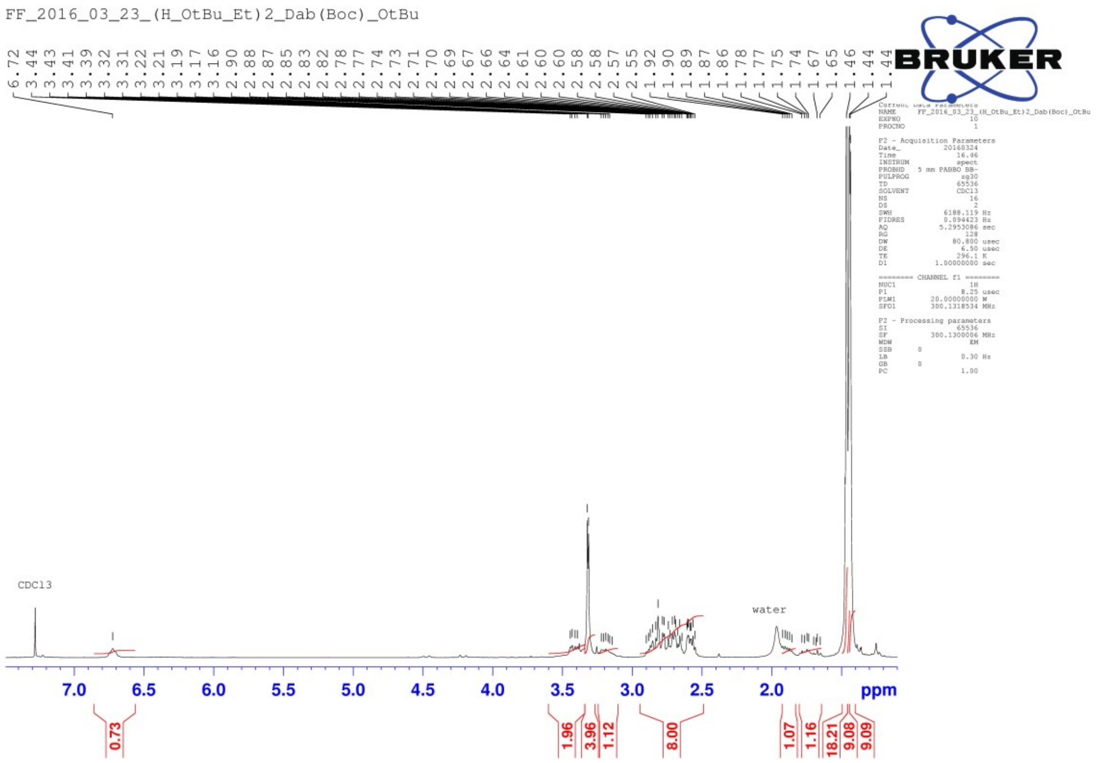

Figure S39: ^{1}H-NMR of compound **11**.

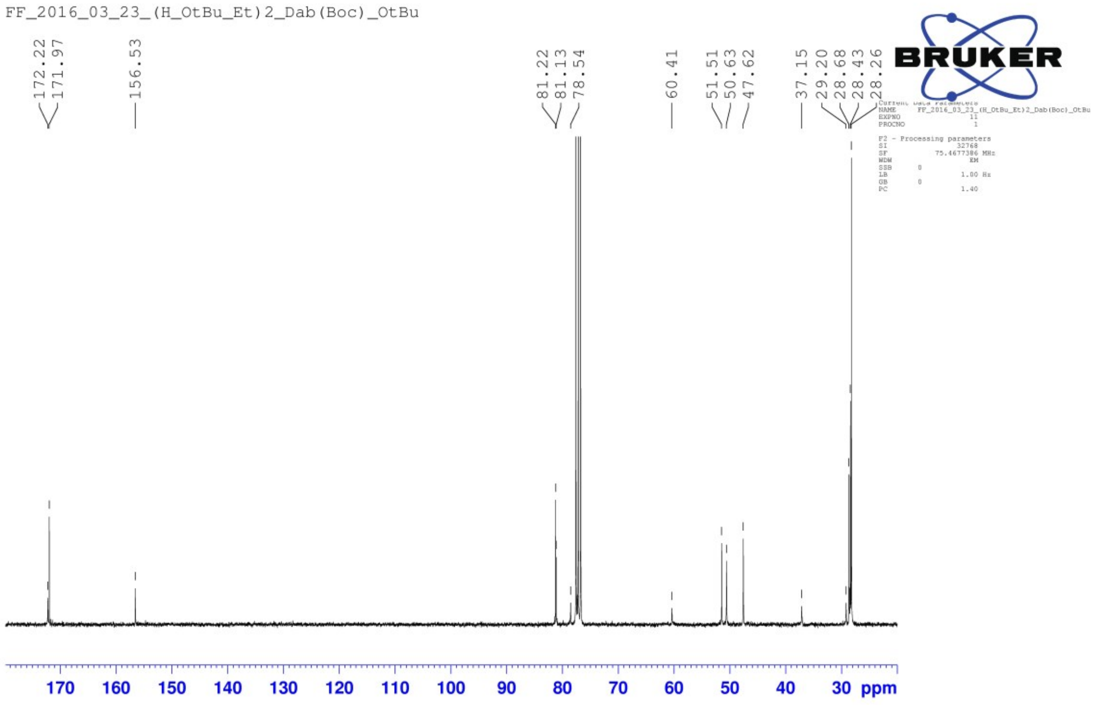

Figure S40: ^{13}C-NMR of compound **11**.

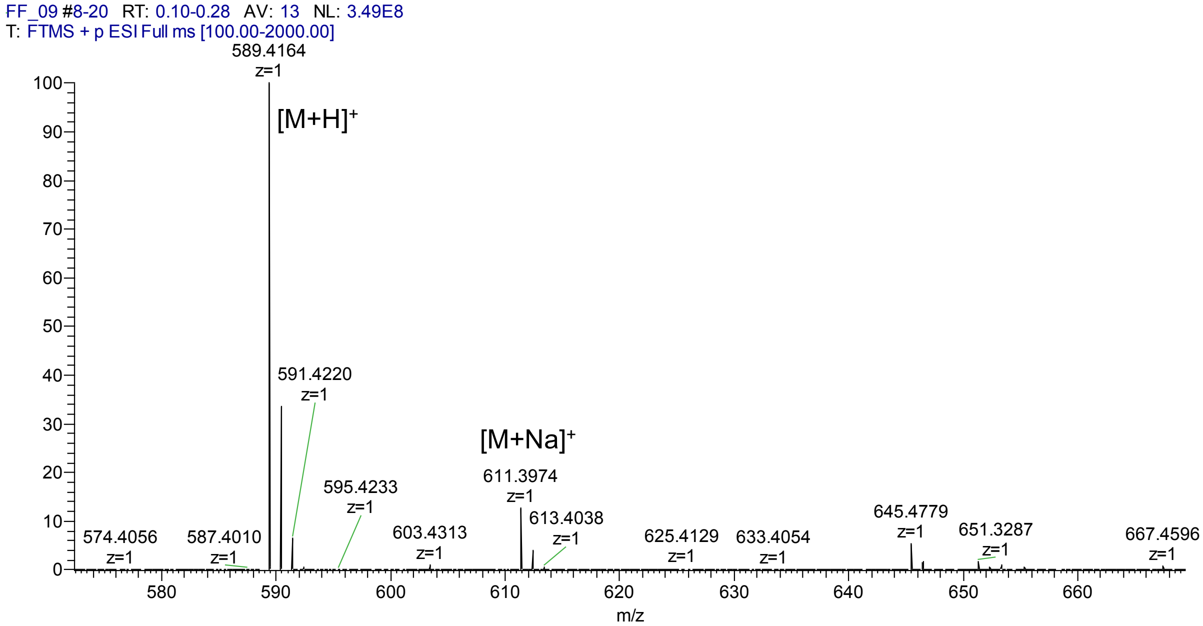

Figure S41: High resolution mass-spectrum of compound **11**.

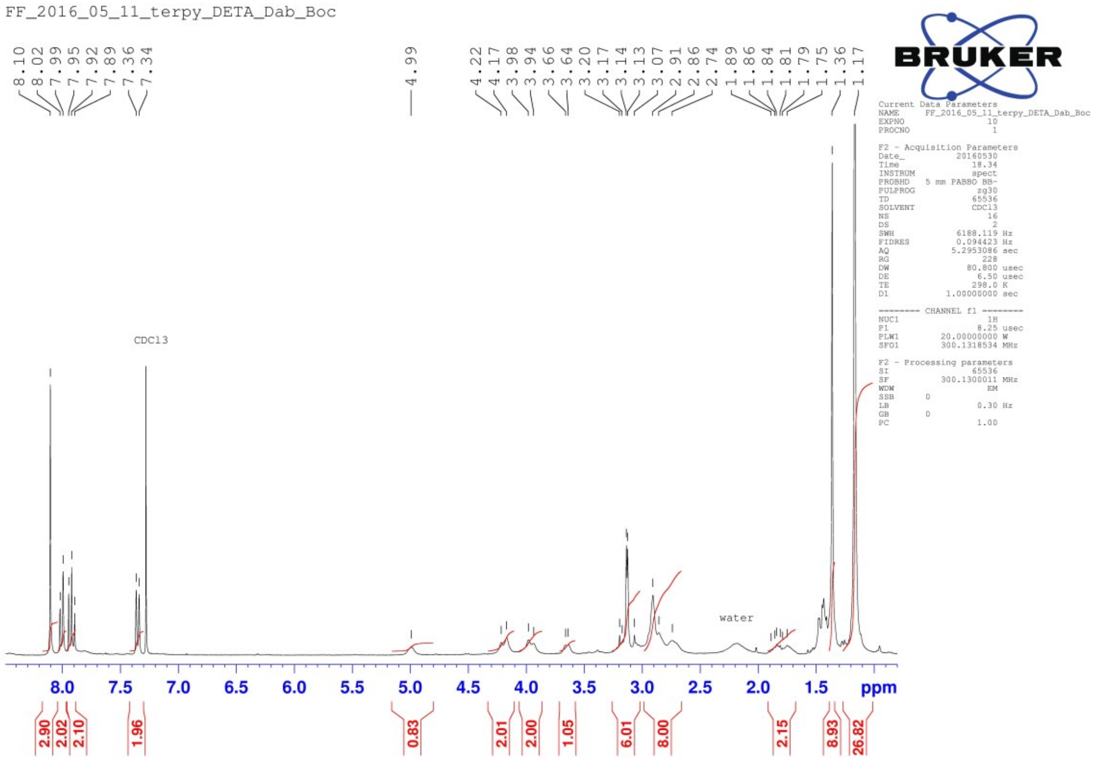

Figure S42: ^{1}H-NMR of compound **12**.

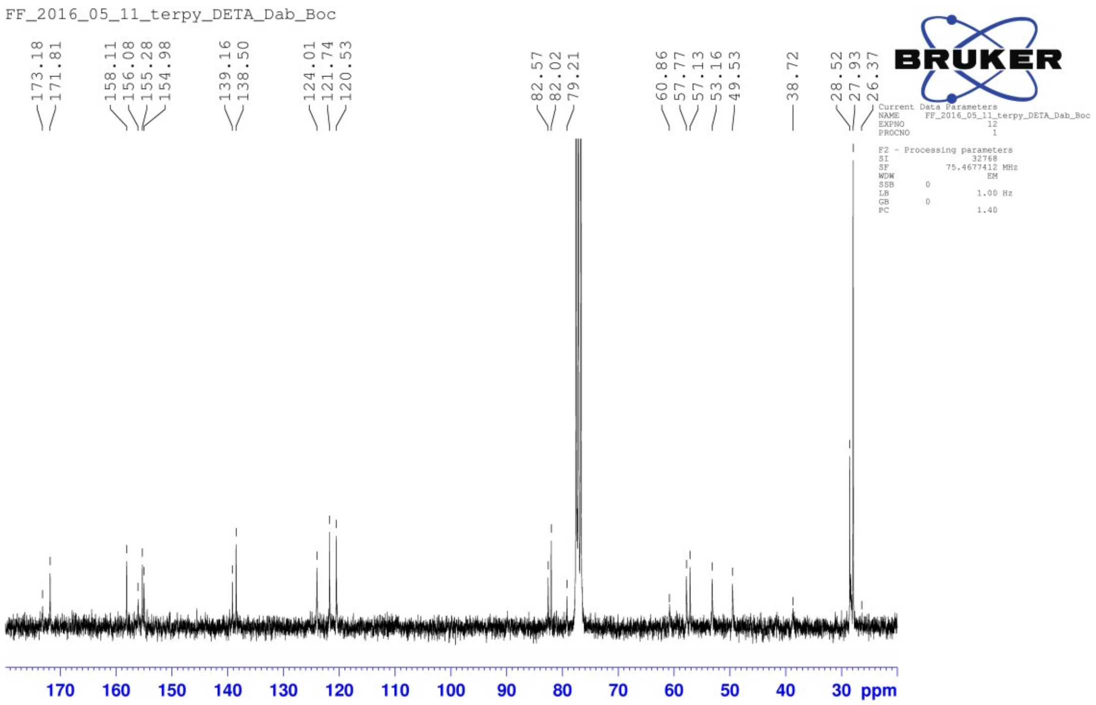

Figure S43: ^{13}C-NMR of compound **12**.

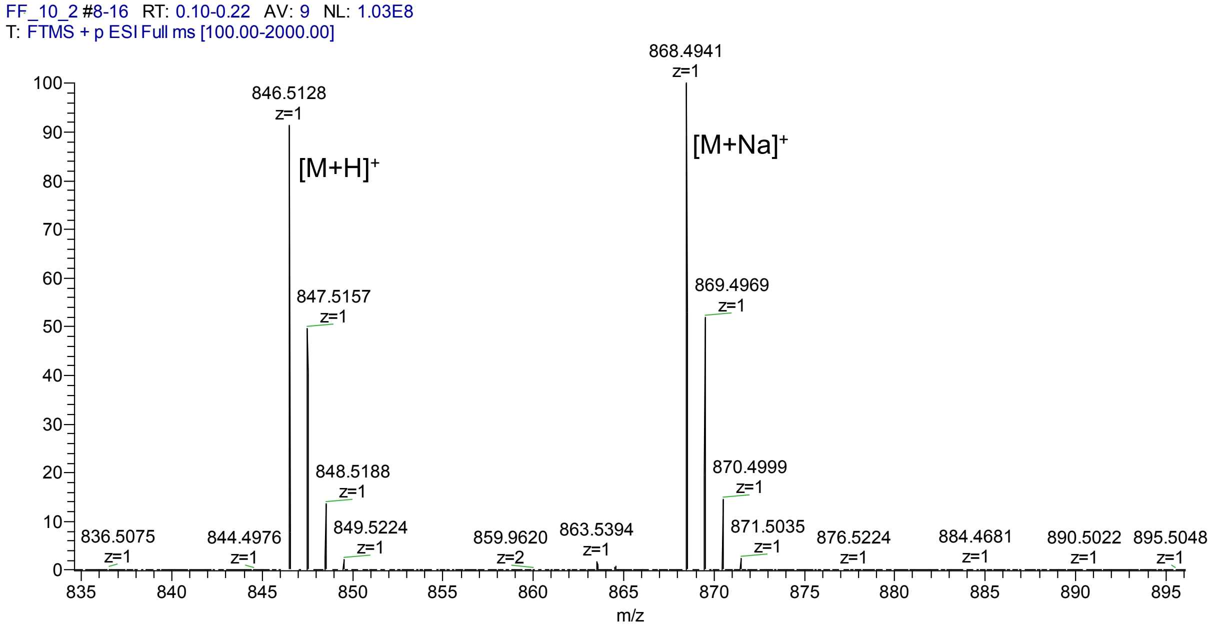

Figure S44: High resolution mass-spectrum of compound **12**.

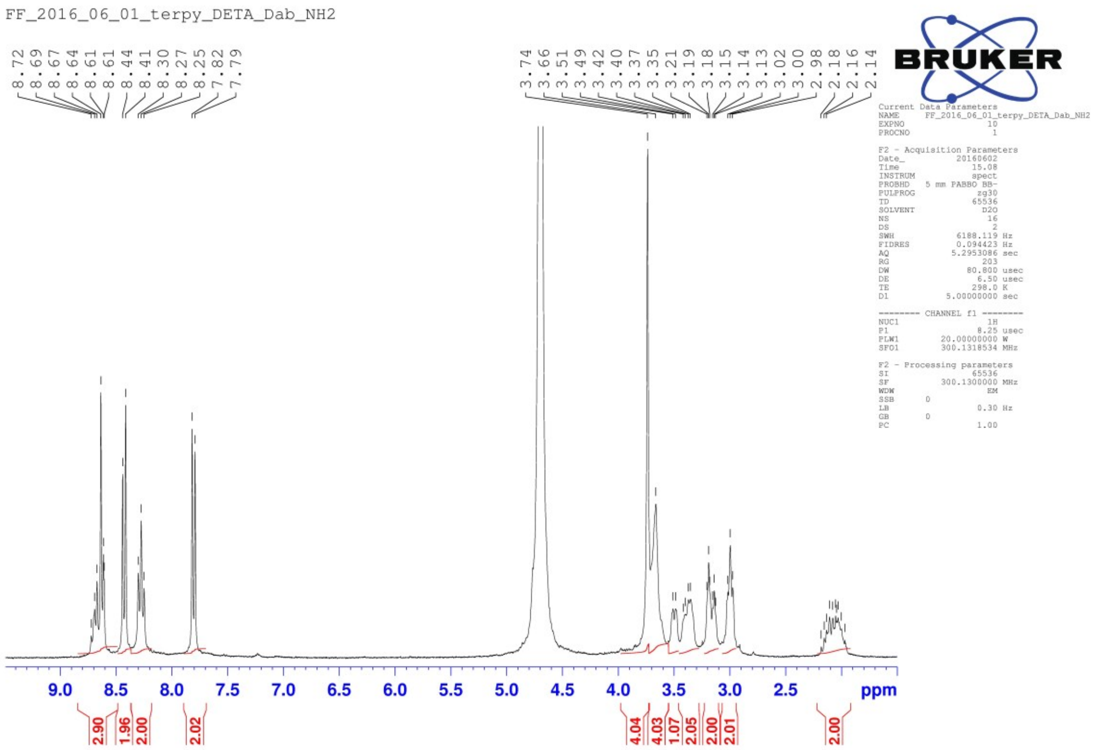

Figure S45: ^{1}H-NMR of compound **13**.

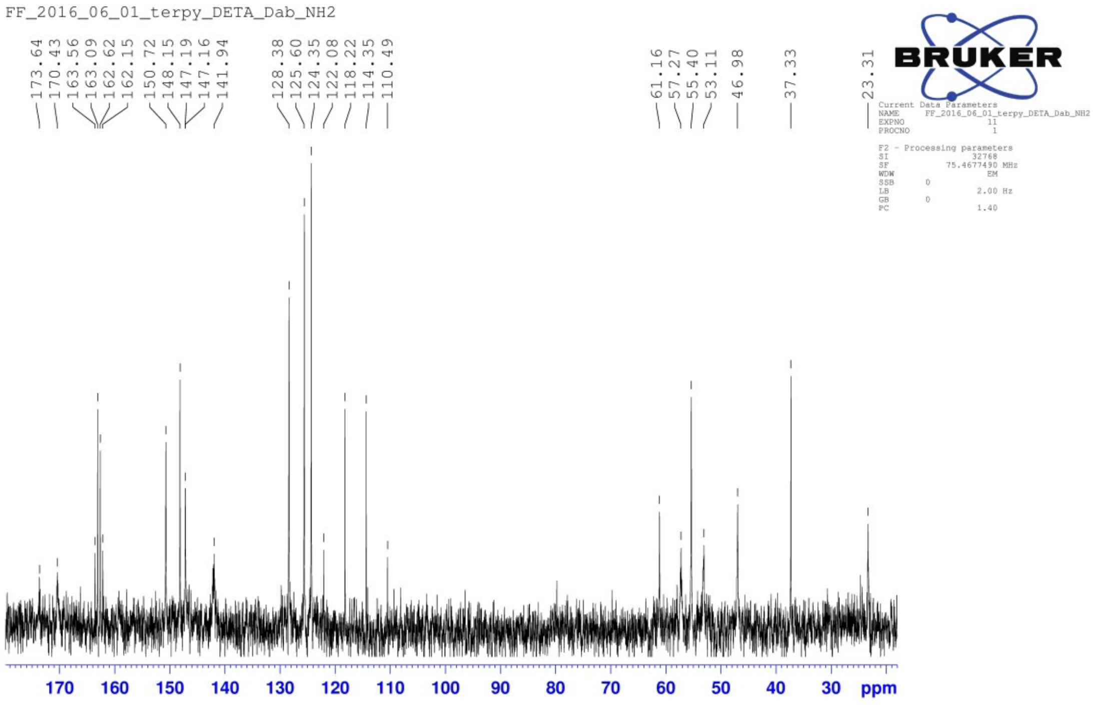

Figure S46: ^{13}C-NMR of compound **13**.

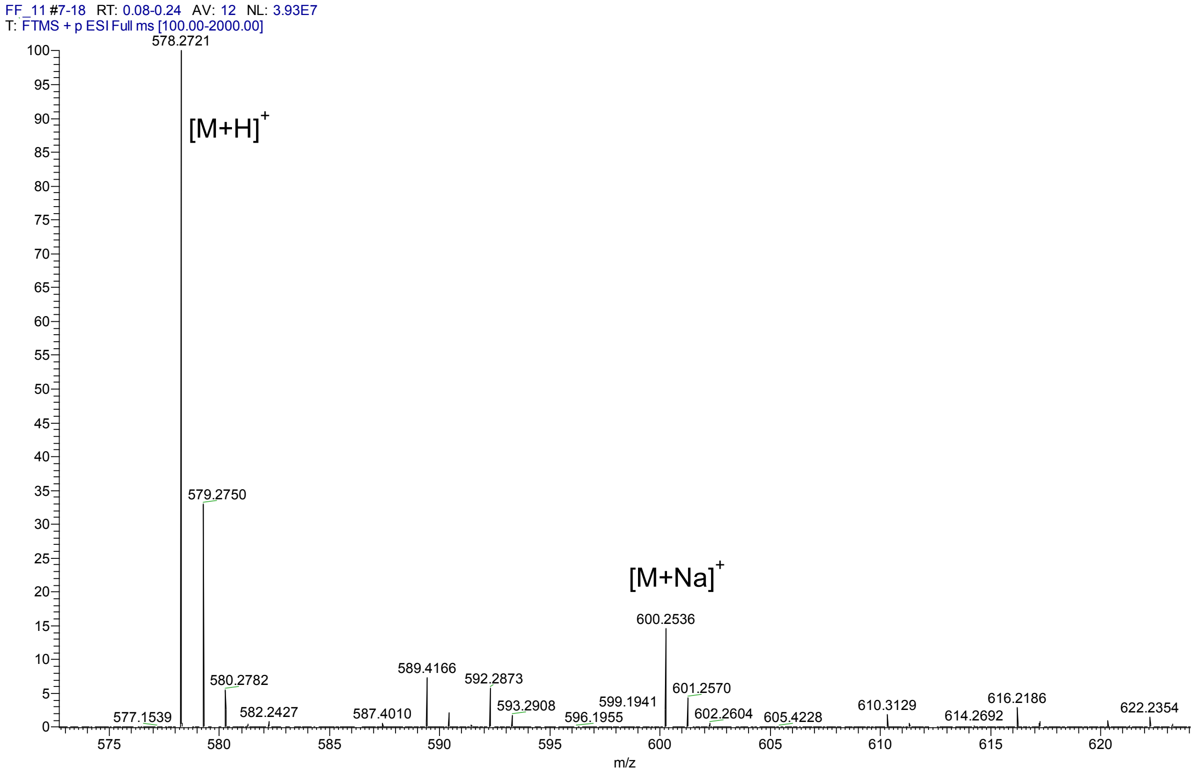

Figure S47: High resolution mass-spectrum of compound **13**.

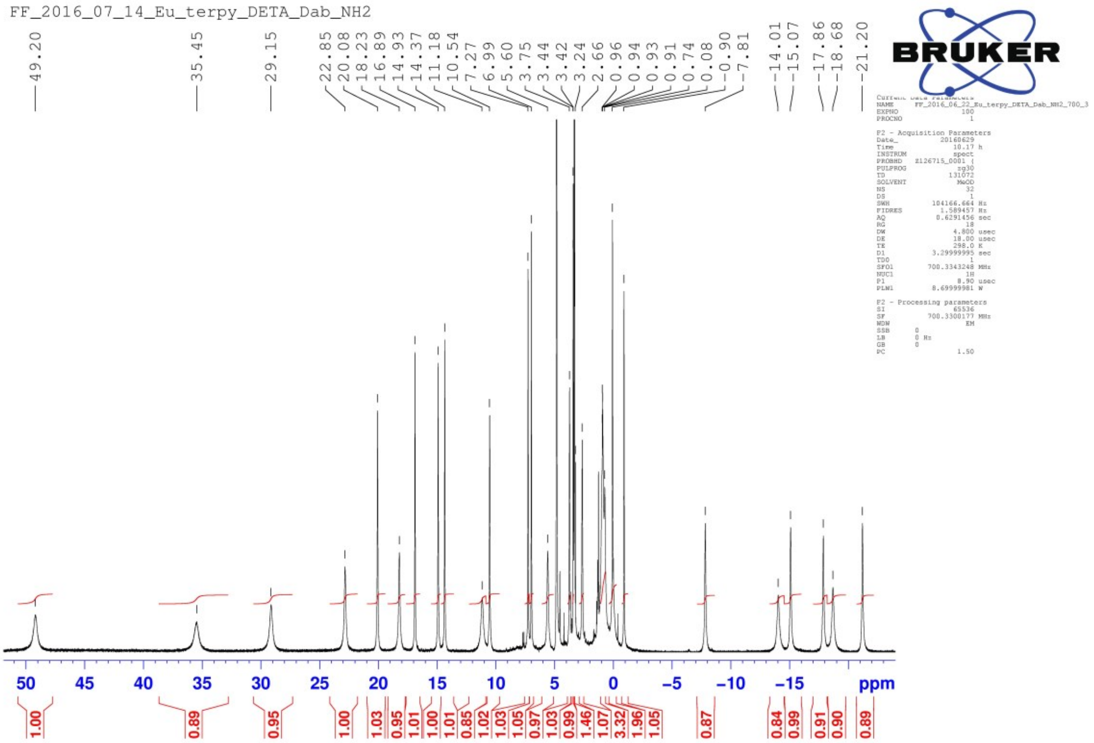

Figure S48: ^{1}H-NMR of compound **13-Eu**.

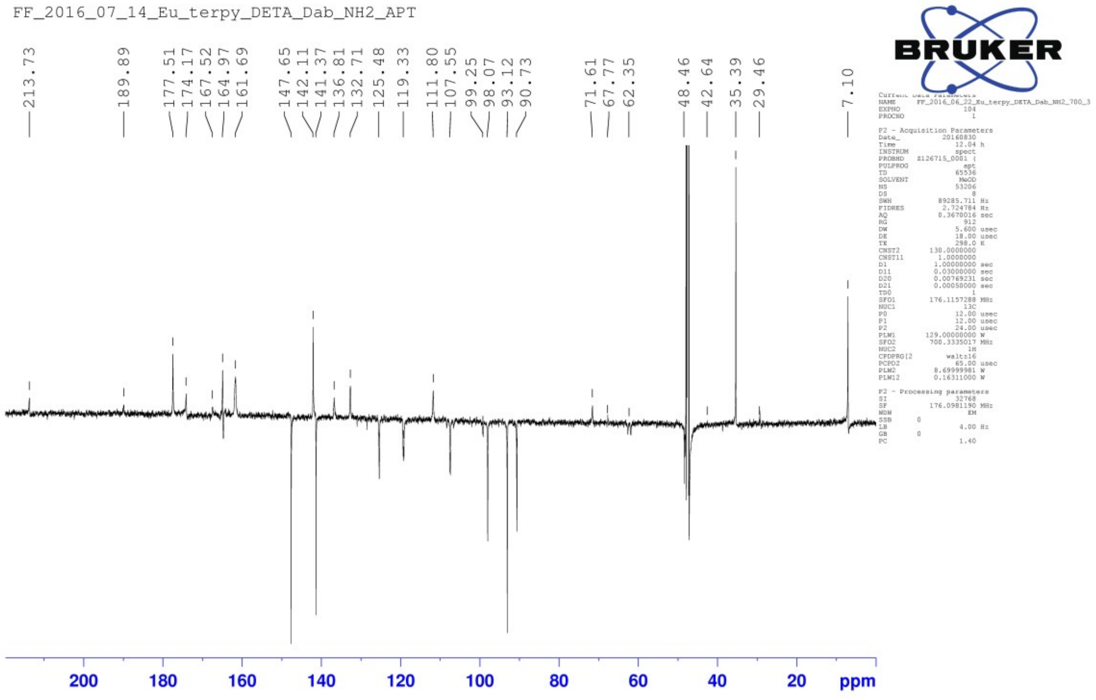

Figure S49: ^{13}C-NMR (APT) of compound **13-Eu**.

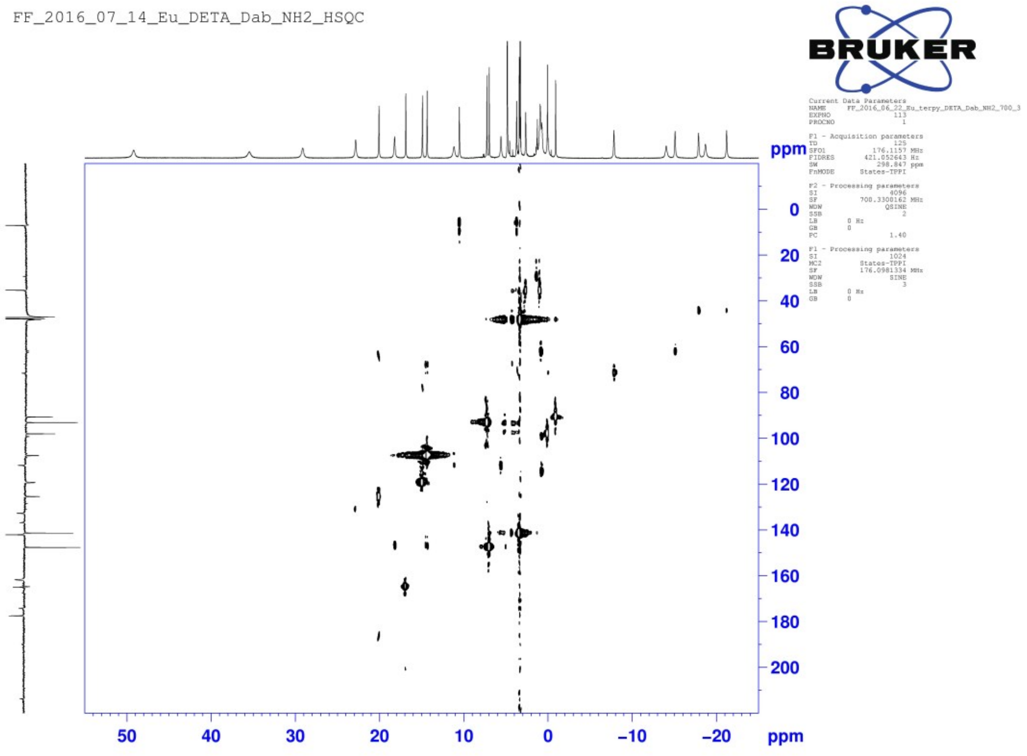

Figure S50: HSQC-NMR of compound **13-Eu**.

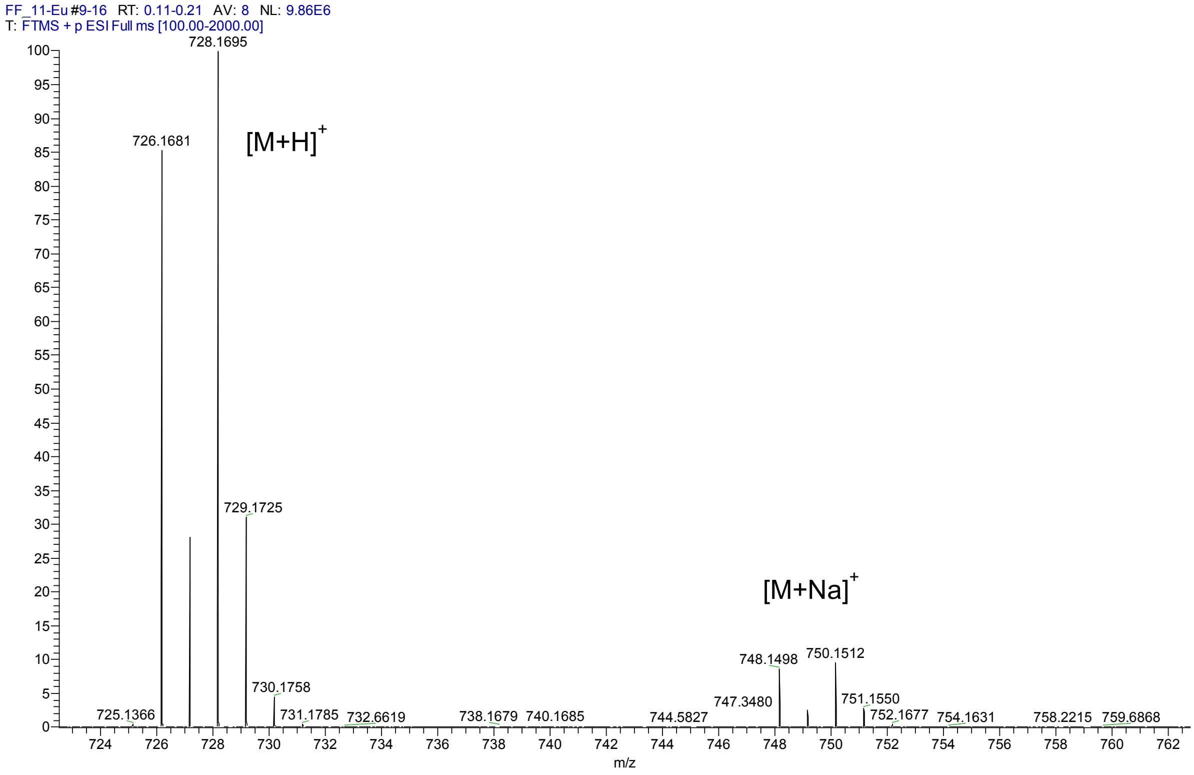

Figure S51: High resolution mass-spectrum of compound **13-Eu**.

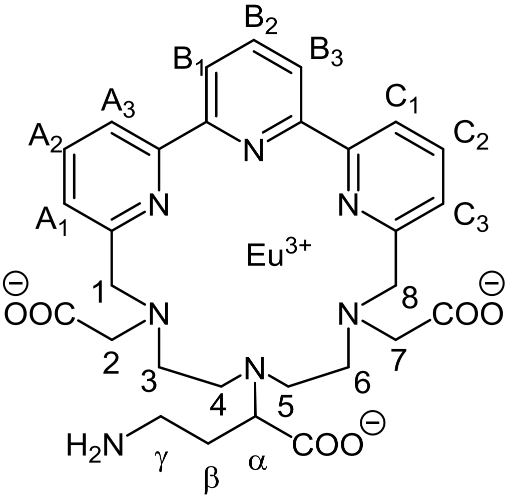

Figure S52: Representation of **13-Eu** with denotation for NMR-signal assignment.

Table S3: NMR-signal assignment.

Sign.	^{1}H-NMR (700 MHz, CD_3OD) δ / ppm	^{1}H-discription	^{13}C-NMR (176 MHz, CD_3OD) δ / ppm	notes
	49.20			[d]
	35.45			[d]
	29.15			[d]
	22.85			[d]
A_x	20.08	(1H, s, CH of A_1 or A_2 or A_3)	125.4	[a]
	18.23			[d]
A_x	16.89	(1H, s, CH of A_1 or A_2 or A_3)	164.9	[a]
A_x	14.93	(1H, s, CH of A_1 or A_2 or A_3)	119.1	[a]
B_x	14.37	(1H, s, CH of B_1 or B_2 or B_3)	107.3	[a]
1	11.18	(1H, s, CH_2 of 1 or 1’)	111.8	[b]
γ	10.54	(1H, s, CH_2 of γ)	7.1	[c]
C_x	7.27	(1H, s, CH of C_1 or C_2 or C_3)	93.1	[a]
B_x	6.99	(1H, s, CH of B_1 or B_2 or B_3)	147.7	[a]
1’	5.60	(1H, s, CH_2 of 1 or 1’)	111.8	[b]
γ	3.75	(1H, s, CH_2 of γ)	7.1	[c]
C_x	3.42	(1H, s, CH of C_1 or C_2 or C_3)	141.4	[a]
α	3.30	(1H, s, CH of α)	48.5	[c]
β	2.66	(1H, s, CH_2 of β)	35.4	[c]
β	0.93	(1H, s, CH_2 of β)	35.4	[c]
2	0.74	(1H, s, CH_2 of 2 or 2’)	62.3	[b]
B_x	0.08	(1H, s, CH of B_1 or B_2 or B_3)	98.2	[a]
3	0.08	(1H, s, CH of 3 or 3’)	71.6	[b]
C_x	-0.90	(1H, s, CH of C_1 or C_2 or C_3)	90.7	[a]
3’	-7.81	(1H, s, CH_2 of 3 or 3’)	71.6	[b]
	-14.01			[d]
2’	-15.07	(1H, s, CH_2 of 2 or 2’)	62.3	[b]
4	-17.86	(1H, s, CH_2 of 4 or 4’)	42.6	[b]
	-18.68			[d]
4’	-21.20	(1H, s, CH_2 of 4 or 4’)	42.6	[b]
			29.5	[d]
			67.8	[d]
			132.7	[d]
			136.8	[d]
			142.1	[d]
			161.7	[d]
			165.0	[d]
			167.5	[d]
			174.2	[d]
			177.5	[d]
			189.9	[d]
			213.7	[d]

[a] The aromatic signals originates from the terpyridine unit, but it cannot be distinguished between the different pyridine rings. With a moderately COSY respectively TOCSY it was possible to assign each three protons which are belonging to the same pyridine ring. [b] The numbered signals are CH_2-groupes which could not be distinguished from each other (They were continuously numbered without further relation to the chemical structure). Due to the paramagnetism of the europium, each proton of one CH_2-group has a different shift and the signals with the same number (distinguished by an apostrophe) belong to the same CH_2-group. [c] With a COSY and TOCSY experiment the signals α, β and γ could be assigned. [d] Most of these signals are quaternary carbons, but some of them are CH_2-groups which are exposed to an outstanding high paramagnetism and therefore the relaxation is too fast to detect any C-H or H-H correlations (e.g. in HSQC or COSY).

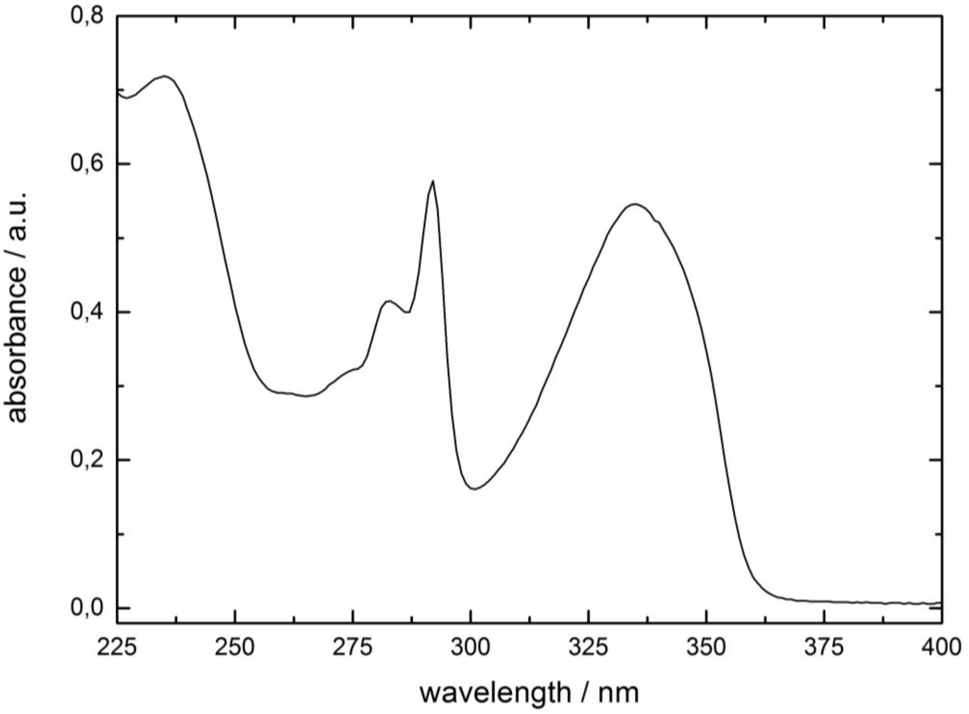

Figure S53: UV-VIS spectrum of compound **13-Eu** in water.

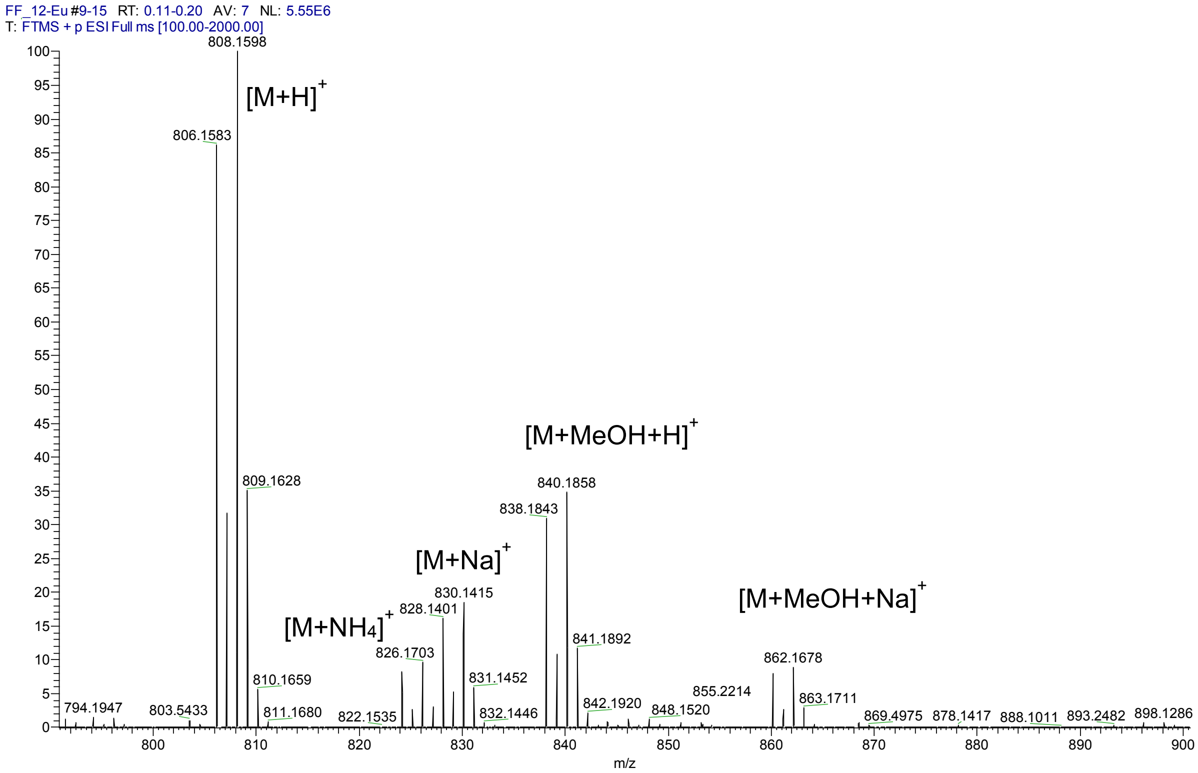

Figure S54: High resolution mass-spectrum of compound **14-Eu**.

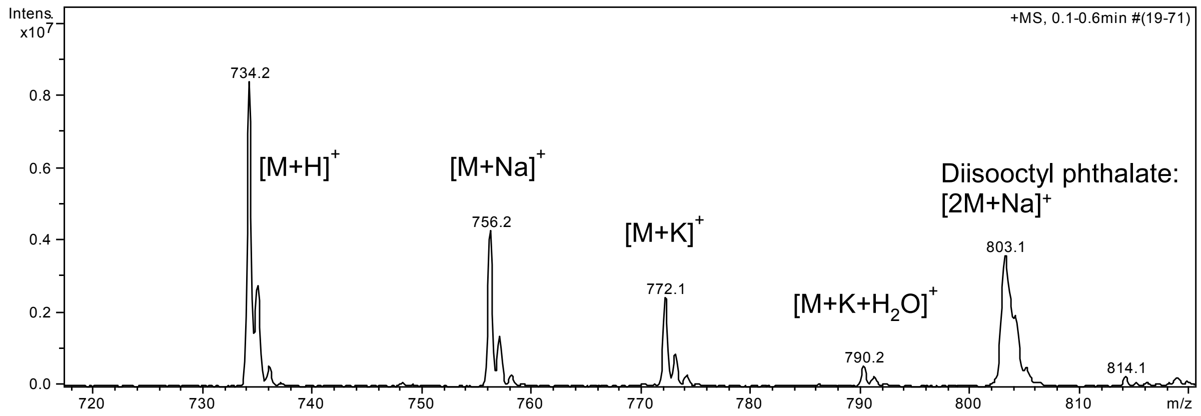

Figure S55: Mass spectrum of compound **13-Tb**.

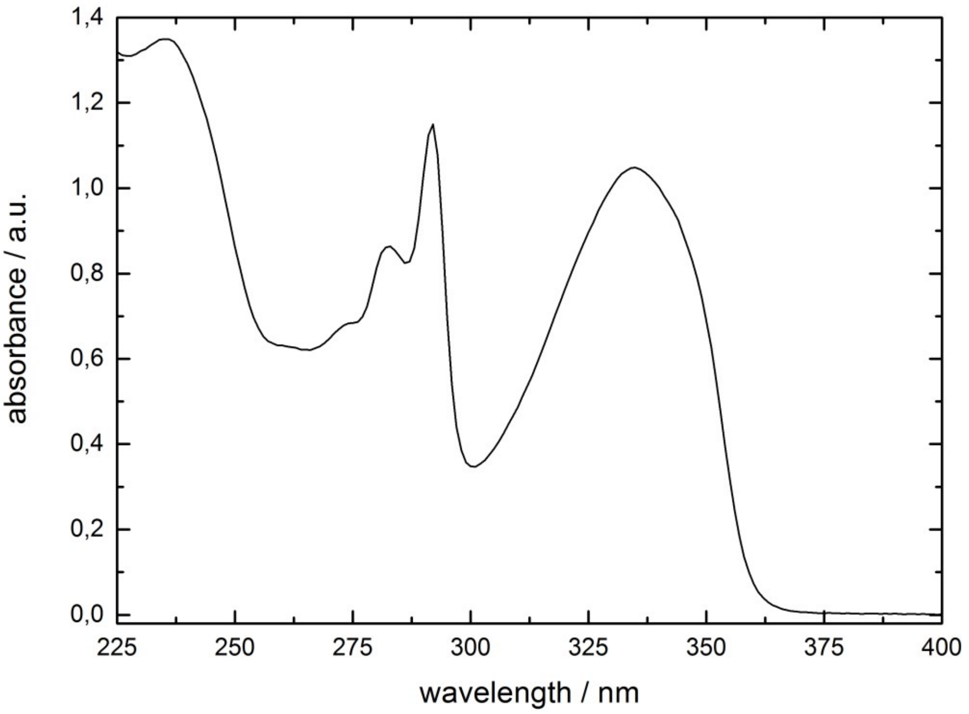

Figure S56: UV-VIS spectrum of compound **13-Tb** in water.

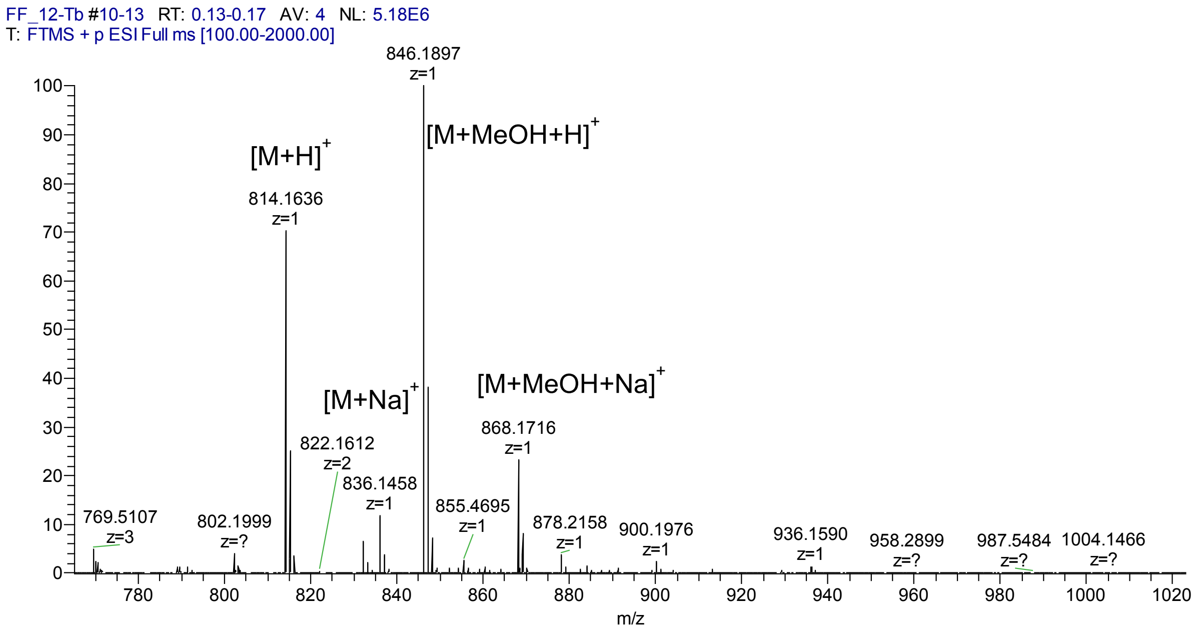

Figure S57: High resolution mass-spectrum of compound **14-Tb**.

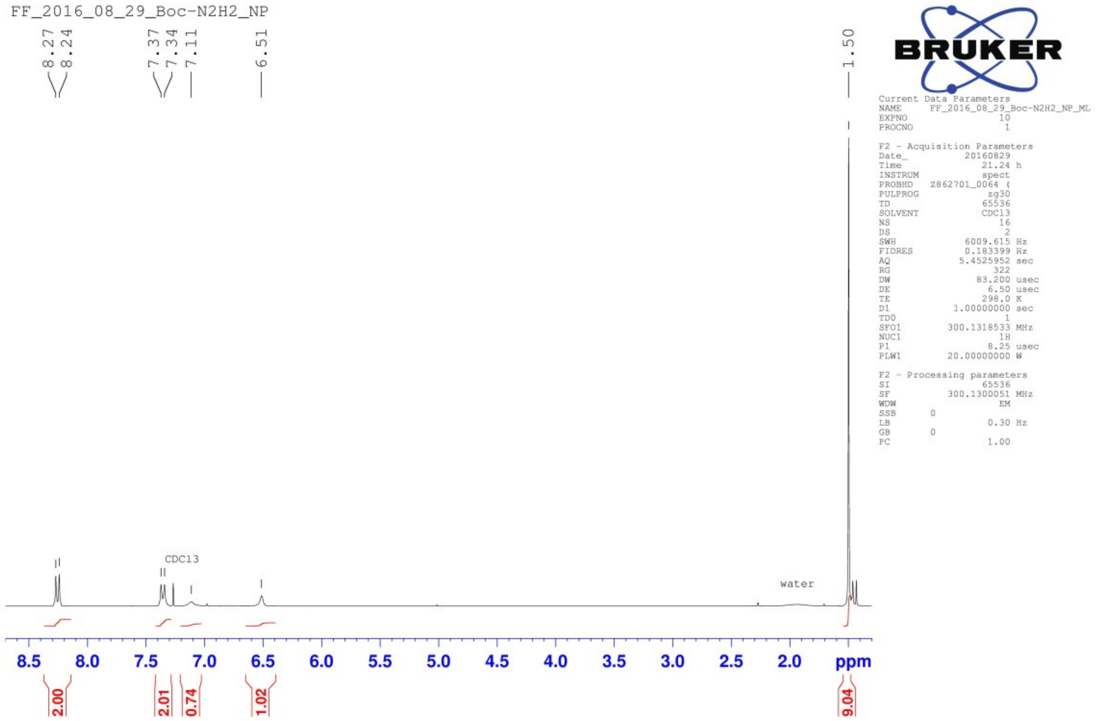

Figure S58: ^{1}H-NMR of compound **15**.

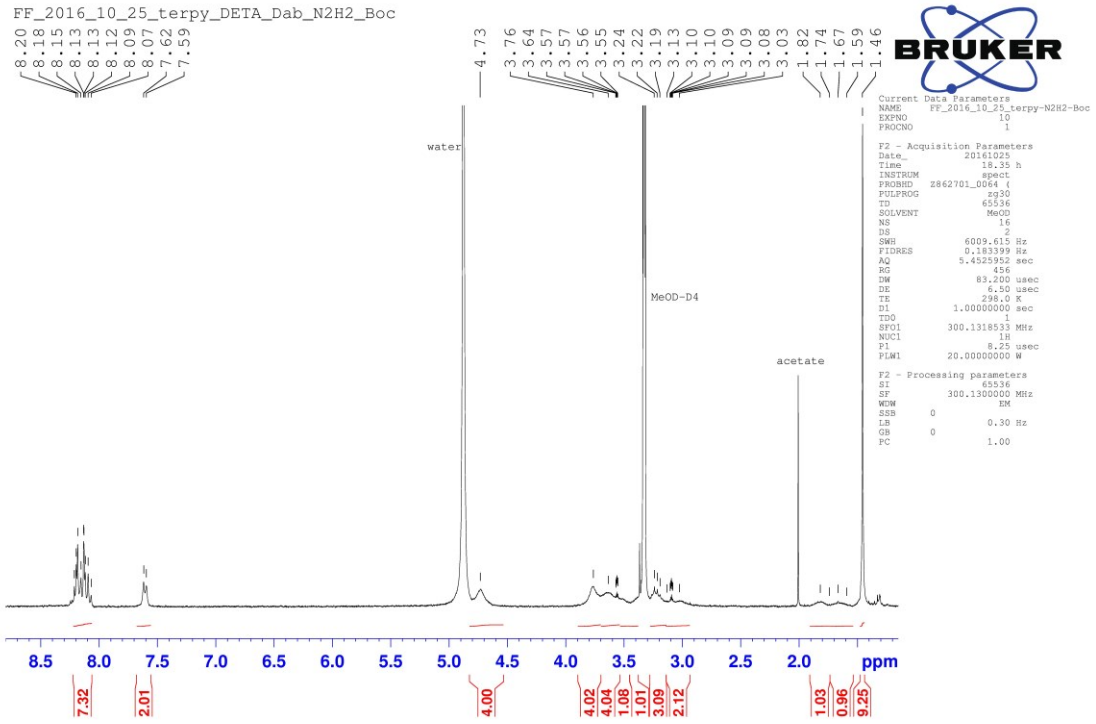

Figure S59: ^{1}H-NMR of compound **16**.

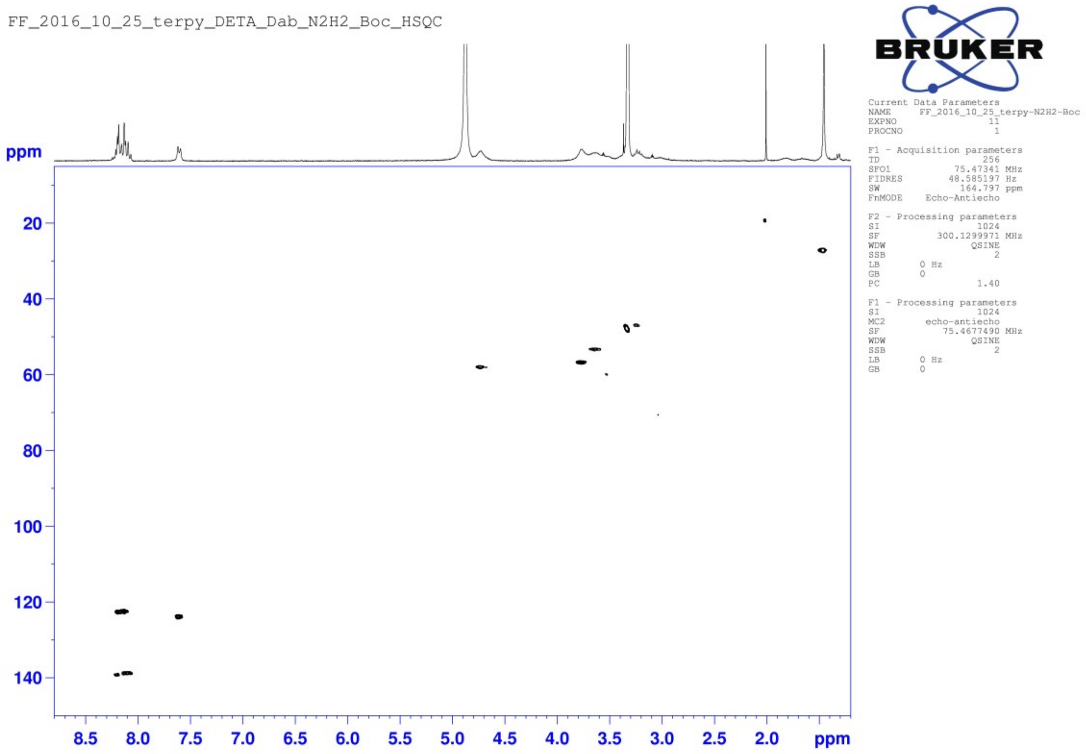

Figure S60: HSQC-NMR of compound **16**.

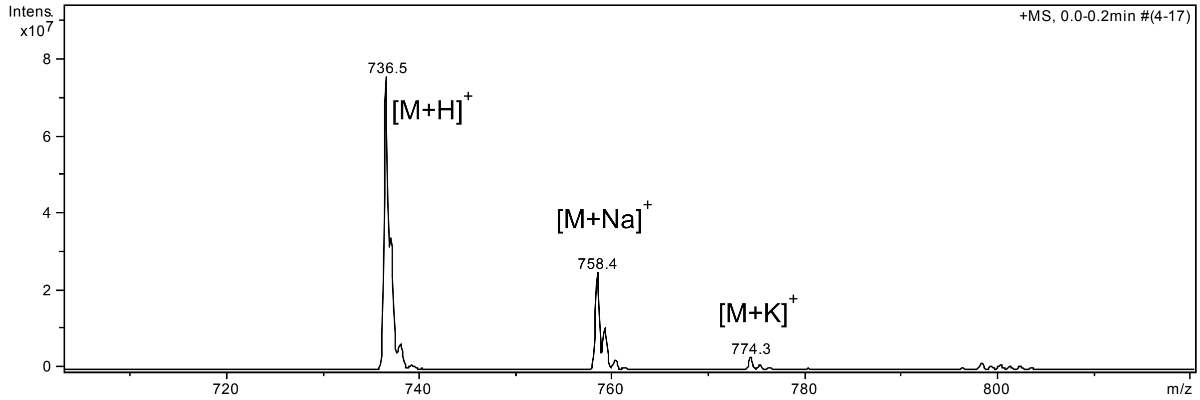

Figure S61: Mass spectrum of compound **16** (positive mode).

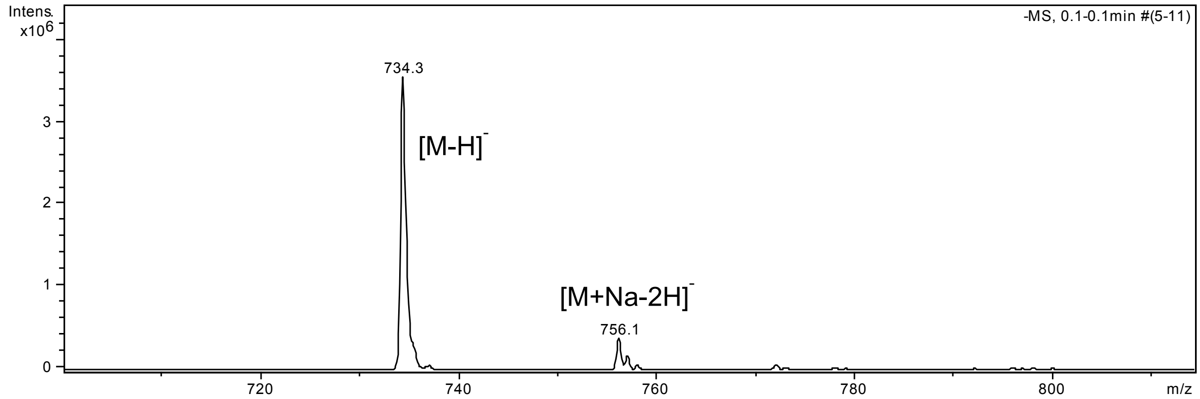

Figure S62: Mass spectrum of compound **16** (negative mode).

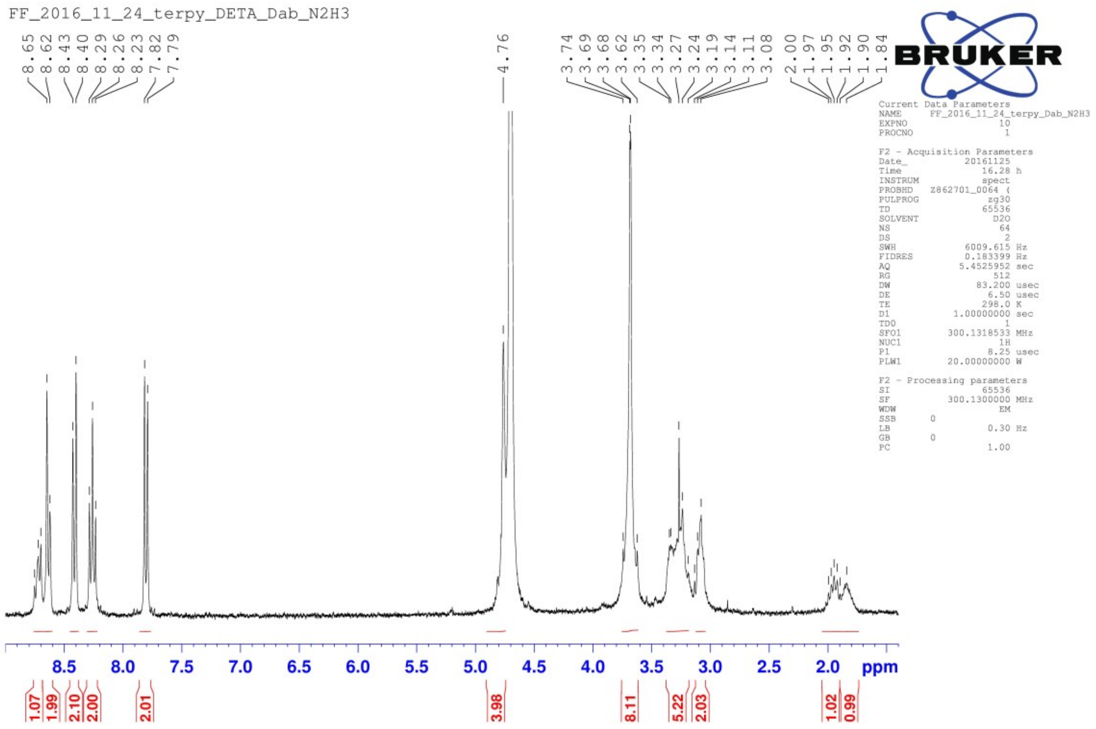

Figure S63: ^{1}H-NMR of compound **17**.

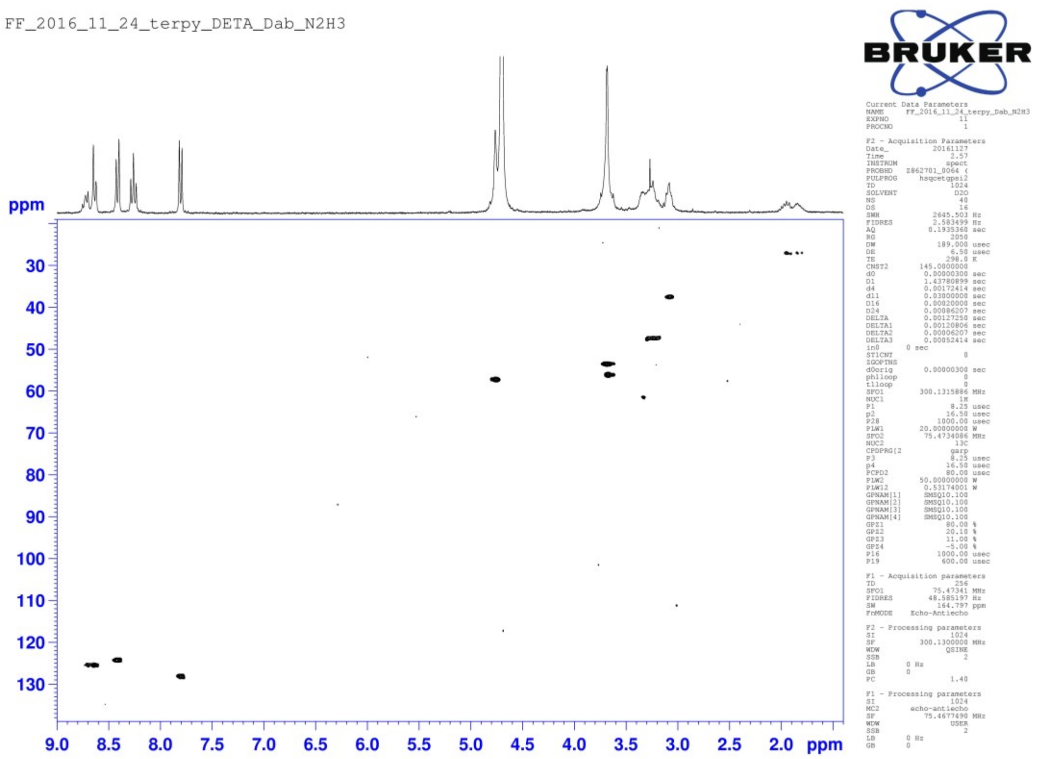

Figure S64: HSQC-NMR of compound **17**.

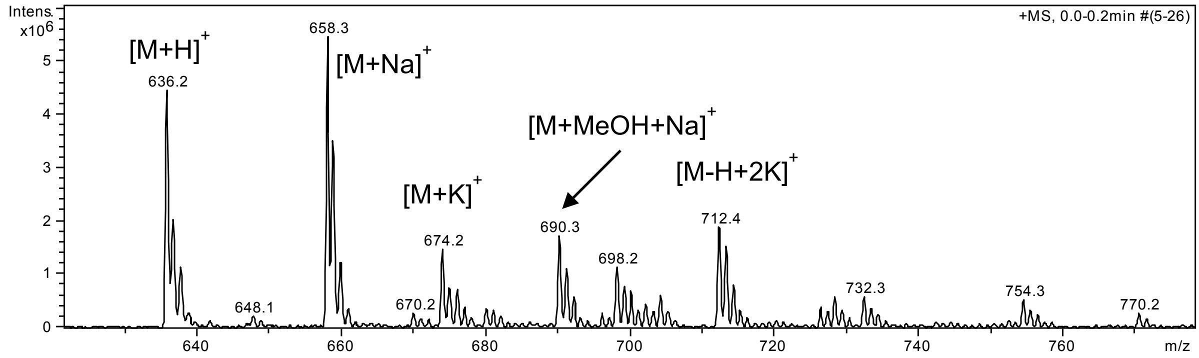

Figure S65: Mass spectrum of compound **17**.

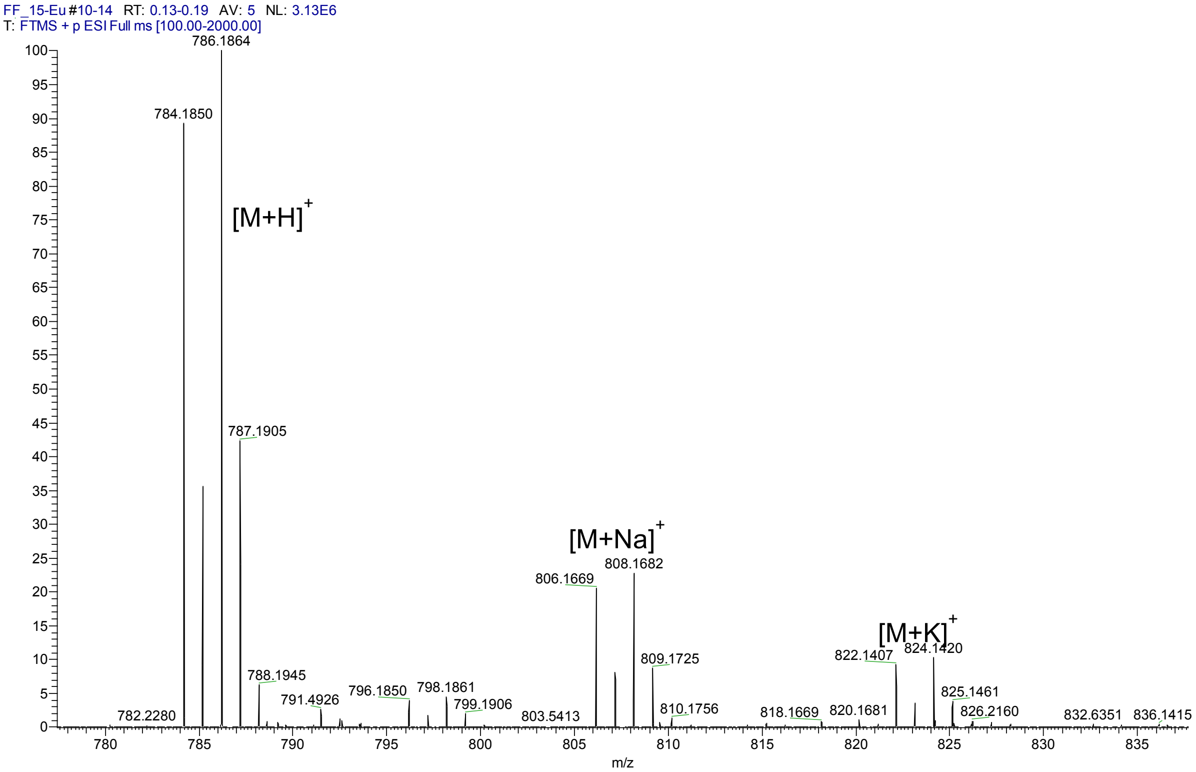

Figure S66: High resolution mass-spectrum of compound **17-Eu**.

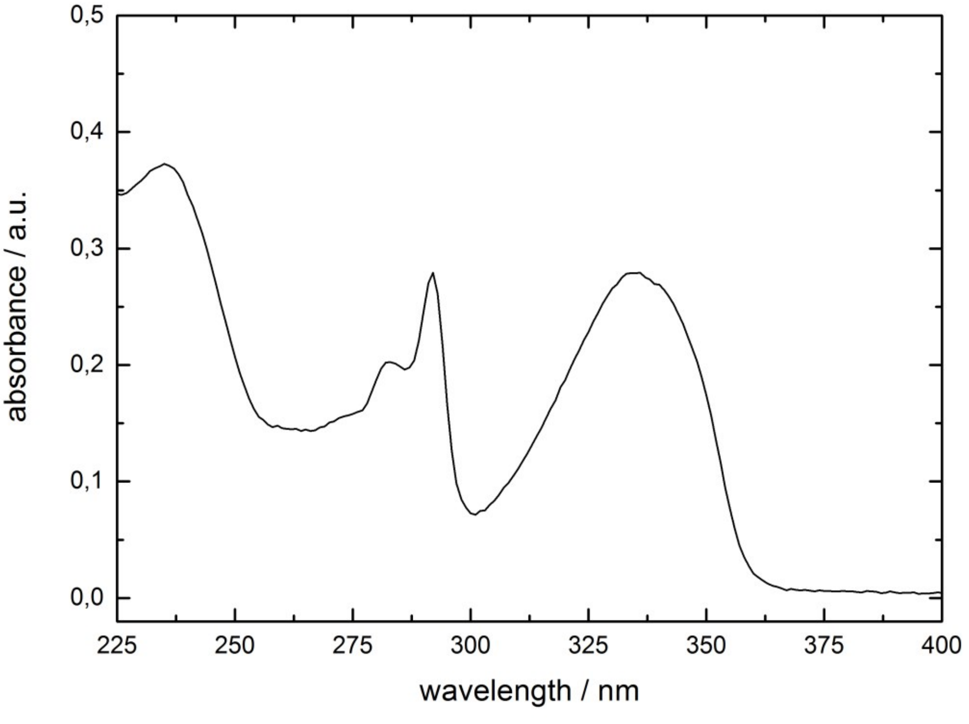

Figure S67: UV-VIS spectrum of compound **Eu-17** in HBS 7.3 buffer.

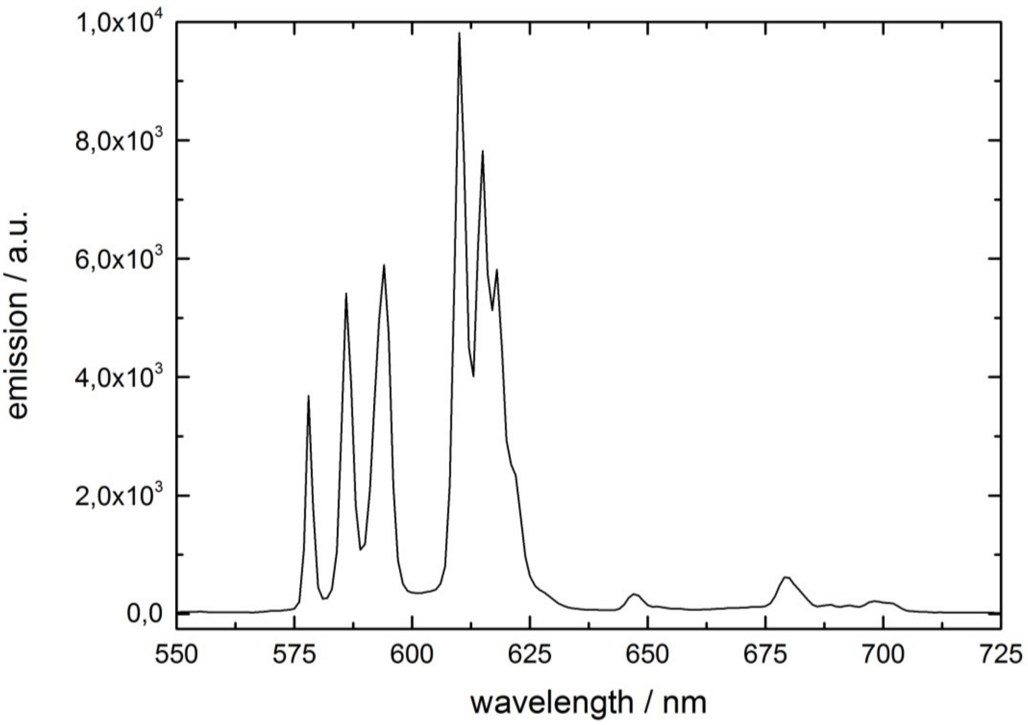

Figure S68: Emission spectrum of compound **Eu-17** in HBS 7.3 buffer, excitation at 335 nm.

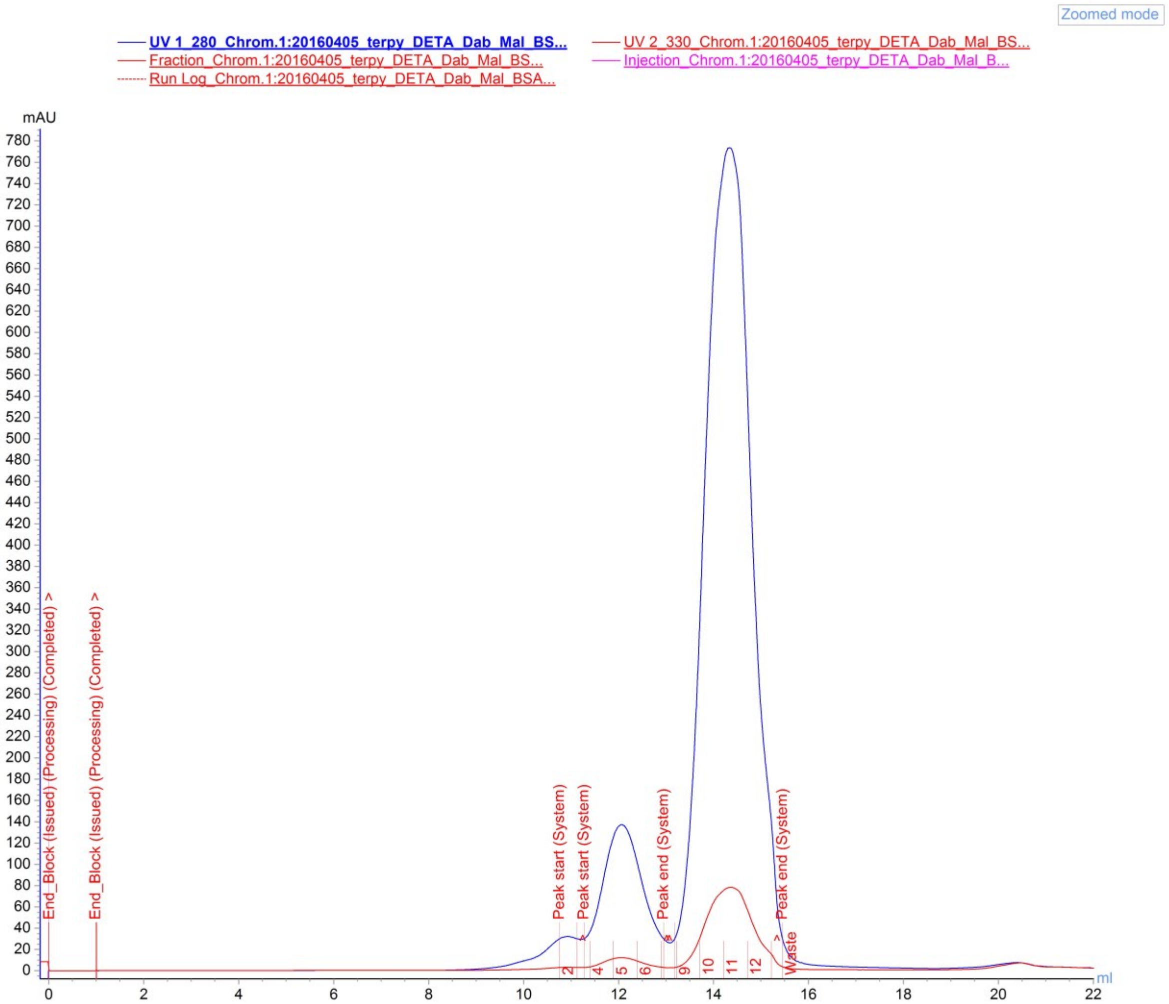

Figure S69: Size-exclusion chromatogram of **14-Eu**-labeled BSA, detecting at 280 and 330 nm absorbance. Fractions 9 – 12 containing the labeled BSA were collected, while the other fractions were discarded.
